# Vibrational Stabilization in Cyclacene Carbon Nanobelts

**DOI:** 10.1021/acs.jpca.5c04863

**Published:** 2025-09-04

**Authors:** Magnus W. D. Hanson-Heine

**Affiliations:** School of Chemistry and Chemical Engineering, University of Southampton, Highfield, Southampton SO17 1BE, U.K.

## Abstract

Cyclacene carbon nanobelts are predicted to be more stable in certain vibrational states. Vibrational simulations using hybrid thermally assisted-occupation density functional theory (TAO–DFT) predict small but consistent singlet–triplet electronic excitation energy changes at the classical harmonic vibrational turning points of the smaller belts. Geometric and vibrational properties are also compared between hybrid Kohn–Sham DFT and TAO–DFT for [n]­cyclacene (*n* = 6–14), where TAO–DFT is found to shorten the carbon–carbon bonds bridging between the two annulene ribbons and causes qualitative changes in the calculated infrared spectra. These geometric changes lower the singlet–triplet transition energies and introduce greater ring strain, while individual vibrational modes are observed to shift by over 200 cm^–1^. These findings indicate that including static correlation is important for describing both the geometric and vibrational properties of cyclacenes accurately.

## Graphical abstract

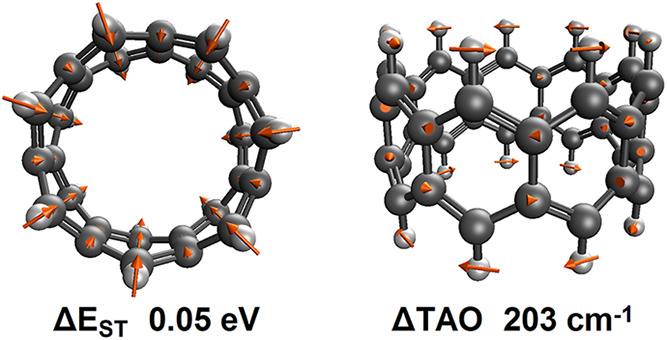

## Introduction

Cyclacene carbon nanobelts were first reported by Heilbronner in 1954.^1,2^ They are the shortest possible hydrogen-capped zigzag carbon nanotubes and the last minimal building blocks of carbon nanotubes to remain unsynthesized. Cyclacenes can be considered as either a fused loop of benzene rings (see Figure 1), or as two fused trans-polyene ribbons,^3^ with odd numbers of fused benzene rings showing lower relative stabilities due to cryptoannulenic effects.^4^ Cyclacenes can provide insights into carbon nanotube properties, where they have been used as finite-length models,^5,6^ and may also act as templates for bottom-up nanotube synthesis with opportunities for early structural diversification and functionalization compared to traditional approaches.^7^ Cyclacenes are predicted to have tunable nonlinear optical properties, making them good candidates for nanoscale circuitry and integration into semiconductors and transistors,^8^ and they have been proposed as originators of the unidentified diffuse infrared bands in the interstellar medium.^9,10^ Möbius cyclacenes are also been predicted to have nonlinear optical properties,^11,12^ to act as spin current rotors,^13,14^ to possess torus screw rotation symmetry,^15^ and to act as chiral discriminators for amino acids.^16^

**Figure 1:**
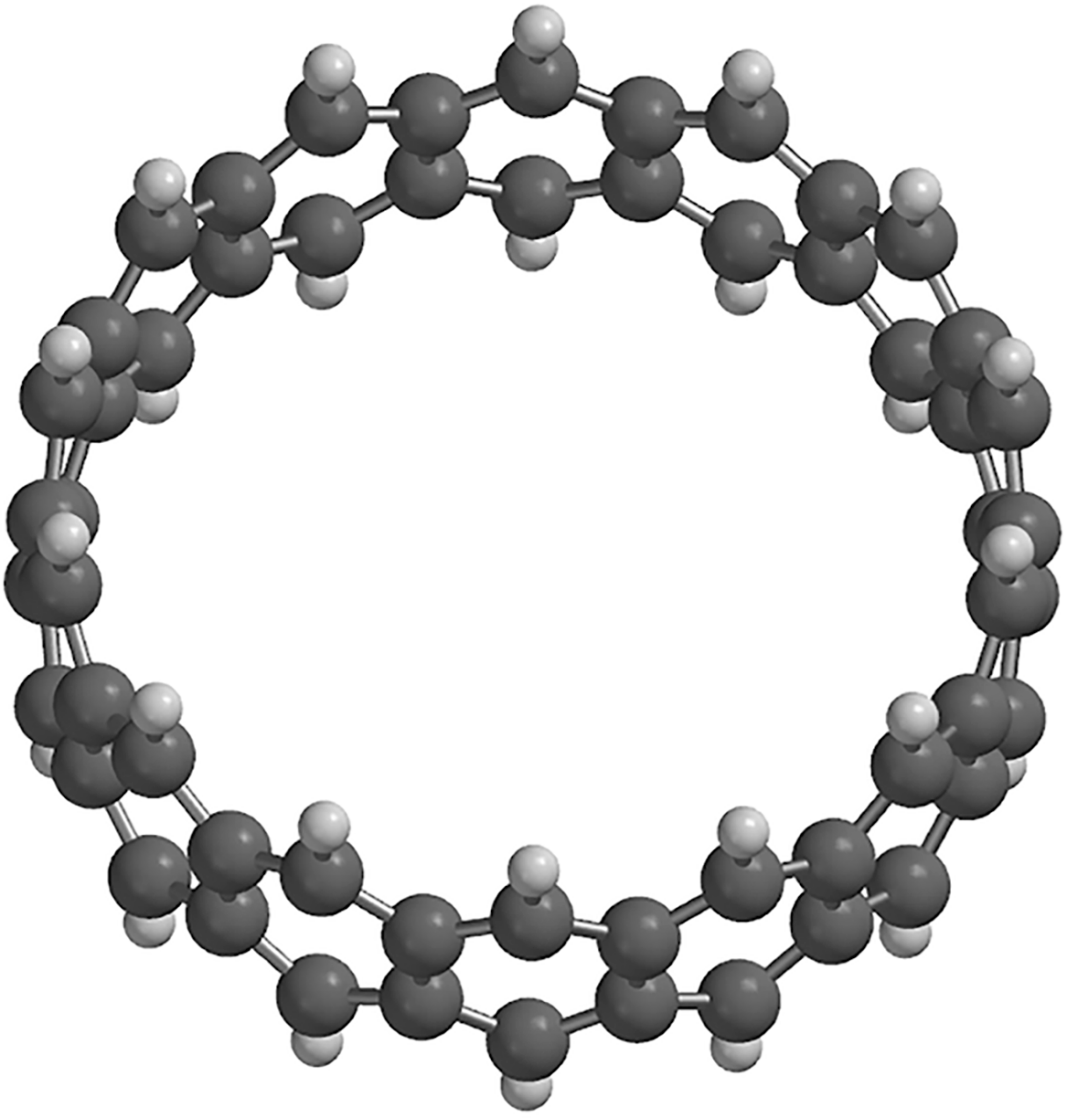
Geometry of [14]­cyclacene calculated using TAO–DFT.

Synthetic attempts at making cyclacenes have been unsuccessful to date,^17–21^ with the synthetic difficulties attributed to a combination of high ring strains,^10,22–24^ low singlet–triplet excitation energies, and significant open-shell polyradical character in their electronic ground states.^23–29^ Instabilities of this kind are predicted to persist under both guest–host interactions^30^ and crystal formation,^31^ while reactions forming fused cyclacene dimers are expected to be highly exothermic.^32,33^ Cyclacene isomers containing fused Dewar benzene within the belt structure are also predicted to be considerably more stable than the purely arenoid cyclacene belt isomers at smaller sizes,^34,35^ and similar Dewar benzene containing belt structures are indicated as metastable in related cyclophenacene belt isomers.^36^

Cyclacenes can also be challenging from a theoretical standpoint, as their high polyradical character makes them strongly open-shell multireference systems. Using a single set of electron spin orbitals neglects the significant “static electron correlation” component of the electronic energy, and as Kohn–Sham density functional theory (KS-DFT)^37^ suffers from a neglect of static correlation, this can lead to qualitative errors in calculated cyclacene properties.^23,28,38,39^ In extreme cases these errors have led researchers to incorrectly assign the cyclacene electronic ground states as having triplet multiplicity.^39^ One of the more efficient methods for overcoming the limitations in describing static correlation is thermally assisted-occupation density functional theory (TAO–DFT),^40^ which makes use of fractional orbital occupations. However, while hybrid exchange-correlation functionals with exact orbital exchange are well established in KS-DFT, they are relatively new to TAO–DFT.^41^ Consequently, the majority of cyclacene research has made use of either fully KS-DFT,^5,6,8,23,31,38,39^ or nonhybrid TAO–DFT energy calculations performed at hybrid KS-DFT nuclear geometries.^30,32,34,35^ However, molecular vibrational frequencies are known to be significantly affected by both static correlation in multireference systems,^42,43^ and requiring hybrid quality energy surfaces in order to describe them properly.^44^ The aim of this study is therefore to characterize how the use of hybrid TAO–DFT can affect cyclacene geometries and vibrational properties, as well as the energies that are associated with them.

## Computational Details

KS-DFT and TAO–DFT calculations have been performed using the Q-Chem 6 quantum chemical software.^45^ KS-DFT calculations were carried out using the unrestricted formulation, with α-spin and β-spin orbitals treated separately. Initial nuclear geometries were optimized to minimum energy structures for [n]­cyclacene (*n* = 6–14), and these geometry minima were confirmed through the absence of imaginary harmonic nuclear vibrational frequencies. The harmonic vibrational frequencies and associated normal mode coordinates were calculated within the nuclear harmonic approximation by diagonalizing the mass-weighted Hessian matrix of each molecule.^46^ Hessian matrices were calculated from finite differences of the analytical nuclear energy gradients with respect to displacements using a finite difference step-size of 1.88973 × 10^–5^ a_0_. Infrared (IR) transition intensities and Raman intensities were calculated for these vibrational modes within the standard Kohn–Sham implementation of the double harmonic approximation,^46,47^ and the classical turning points for the oscillators were calculated by setting the Q-Chem input flag *NHO_CTP = TRUE*. The vibrational normal modes have been numbered in order of ascending energy. IR spectra were plotted using the IQMol software^48^ with ca. 47 cm^–1^ full width at half-maximum Gaussian broadening in the main text and ca. 17 cm^–1^ broadening in the Supporting Information, and a vibrational scaling factor of 0.968 was also applied to the IR and Raman spectral transition frequencies.^49^

The lowest electronic states of both singlet and triplet multiplicity were calculated at several nuclear geometries, and vertical singlet–triplet state excitation energies were calculated from the energy differences between the electronic self-consistent field (SCF) solutions for each spin multiplicity. Adiabatic excitation energies were calculated from differences between the singlet and triplet energies at nuclear geometries optimized within each electronic state. Ring strain energies were calculated using the extrapolation scheme described in the text (*vide infra*). All of the single-point electronic energies reported have been calculated using only TAO–DFT at nuclear geometries optimized using both KS-DFT and TAO–DFT in order to isolate nuclear structural effects. Electronic orbitals were plotted using the IQMol software at a 0.02 e/Å^3^ isosurface, and standalone nuclear geometries were plotted with the Spartan ’14 software package.^50^

Electronic structures, energies, and energy gradients were calculated using the Kohn–Sham B3LYP exchange-correlation functional,^51,52^ which was combined with the temperature-dependent energy functional and the exact exchange fictitious temperature-dependent energy functional developed by Chai in the TAO–DFT case,^40,41,53^ both at a fictitious temperature of 18.349 mE_h_.^54^ Grimme DFT-D3 empirical dispersion corrections were added to the DFT energies and gradients using the Modified Becke-Johnson form,^55^ denoted B3LYP-D3M­(BJ). This dispersion correction was previously parametrized for Kohn–Sham calculations using both equilibrium and nonequilibrium structures,^55^ and the default KS-B3LYP-D3M­(BJ) dispersion parameters were used for the TAO-B3LYP-D3M­(BJ) calculations without further modification. Both the exchange-correlation functionals and the dispersion correction have been shown to be particularly accurate for simulating molecular vibrational frequencies.^44,56^ Geometry optimizations and harmonic frequency calculations were carried out using the triple-ζ Pople 6–311G­(d,p) electronic basis set to represent the wave function and electron density,^57^ while the single-point energy calculations were carried out using the larger Dunning aug-cc-pVTZ basis set. All electronic structure calculations were all carried out using the SG-1 numerical integration grid.^58^

## Results and Discussion

### Vertical Singlet–Triplet Excitation Comparison

The TAO–DFT calculations shown here have accounted for static electron correlation, hybrid dynamic electron correlation, and empirical dispersion in the electronic configurations, nuclear geometries, and molecular vibrations. In the first instance these calculations are compared with the author’s previous predictions at the TAO-PBE/6–311+G­(2df,2p)//KS-B3LYP/6–31G­(d) level of theory,^34^ which were calculated prior to hybrid TAO–DFT being implemented in commercially available software. The singlet–triplet vertical excitation energies of these cyclacenes are shown in Table 1, and are found to be higher than previously reported for the odd-numbered cyclacenes and lower than previously reported for the even-numbered cyclacenes. The highest excitation energy is again seen for [8]­cyclacene at 0.34 eV. However, this transition is now ca. 0.11 eV lower than previously reported. The [6]­cyclacene transition energy is also lowered by ca. 0.05 eV, and now falls roughly equal to the [8]­cyclacene transition to within ca. 0.0005 eV. This finding contrasts with previous reports that predict the [8]­cyclacene singlet state to be significantly more stable. However, this prediction should be treated as tentative due to the approximate nature of the methods involved. The frontier molecular orbitals for [8]­cyclacene are shown in Figure 2 for the TAO–DFT orbitals corresponding to the highest occupied molecular orbital (HOMO) and the lowest unoccupied molecular orbital (LUMO) in the absence of thermally assisted-occupation. Both of these orbitals are localized on the outermost carbons atoms of the peripheral annulene ribbons, consistent with previous reports at the 0.01 e/Å^3^ isosurface.^28^

**Figure 2:**
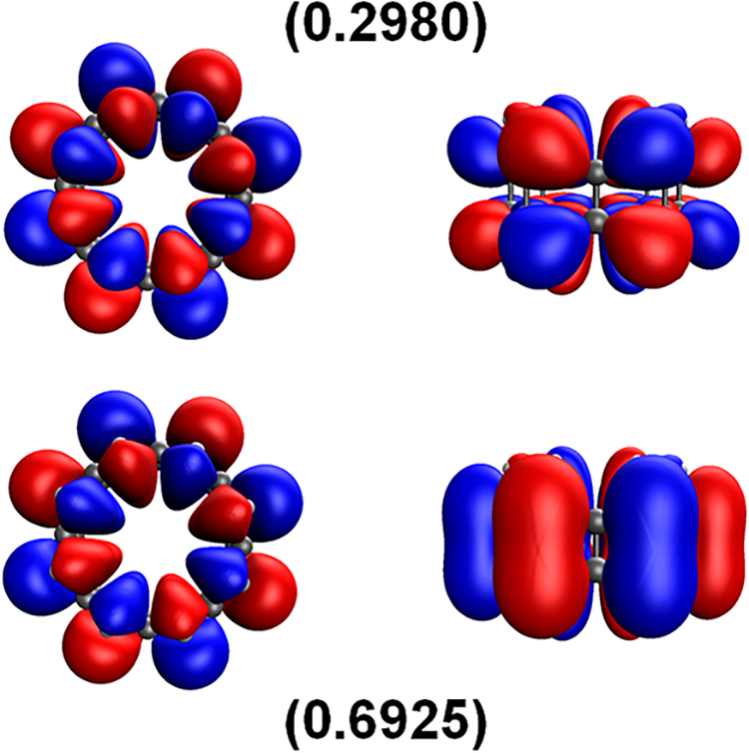
HOMO (bottom) and LUMO (top) α-spin TAO–DFT molecular orbitals for [8]­cyclacene at the 0.02 e/Å^3^ isosurface. The orbitals are shown from the top view (left) and side view (right), and the occupation numbers are given in parentheses. The HOMO is defined here as the (*N*/2)­th α-orbital and the LUMO is defined as the (*N*/2 + 1)­th α-orbital, where *N* is the total number of electrons.

**Table 1:** Vertical Singlet-Triplet Excitation Energies for the [*n*]­Cyclacenes (in eV)

belt size	hybrid-TAO–DFT	GGA-TAO–DFT^a^
[6]cyclacene	0.34	0.39
[7]cyclacene	0.22	0.22
[8]cyclacene	0.34	0.45
[9]cyclacene	0.13	0.08
[10]cyclacene	0.25	0.36
[11]cyclacene	0.12	0.07
[12]cyclacene	0.18	0.25
[13]cyclacene	0.12	0.09
[14]cyclacene	0.13	0.15

a TAO-PBE/6–311+G­(2df,2p)//KS-B3LYP/6–31G­(d). ref 34

### Nuclear Geometric Changes

The principle aim of this study is to analyze the nuclear geometric and vibrational changes that are induced by the inclusion of hybrid TAO–DFT static correlation. In order to do this, cyclacene geometries have been calculated and compared using KS-DFT and the equivalent TAO–DFT functional during geometry optimization. The ΔTAO approach has been used to measure changes between the two methods,^43^ and is defined for energies and bond lengths in eqs 1 and 2. These ΔTAO values are expected to approximate the degree of multireference character, or static electron correlation, present in each property as

1 $$\Delta {\mathrm{TAO}}_{E}=E_{\mathrm{TAO}-\mathrm{DFT}}-E_{\mathrm{KS}-\mathrm{DFT}}$$

and

2 $$\Delta {\mathrm{TAO}}_{R}=R_{\mathrm{TAO}-\mathrm{DFT}}-R_{\mathrm{KS}-\mathrm{DFT}}$$

where *E* and *R* represent energies and bond lengths, respectively, and where the “KS-DFT” and “TAO–DFT” subscripts refer to the method used when calculating the nuclear geometries.

Calculated bond lengths for the [n]­cyclacenes are given in Table 2. These data show that the C–H bonds and the C–C bonds in the outer annulene ribbons remain relatively unaffected by the use of TAO–DFT and change by just ca. 0.001 Å on average. The main exceptions are for [11]­cyclacene and [13]­cyclacene, as these belts suffer from an artificial symmetry lowering in the KS-DFT geometry calculations, and their symmetries are restored in the TAO–DFT geometries. The bond lengths of these symmetry broken belts follow the same trends as the other belt sizes when averaged across the molecules, however, with the annulene ribbon bond lengths alternating between 1.309 and 1.418 Å for [11]­cyclacene and between 1.381 and 1.427 Å for [13]­cyclacene. The largest TAO–DFT effects are concentrated in the C–C bonds that bridge between the two annulene ribbons, which contract almost uniformly by ca. 0.012 Å for the odd numbered cyclacenes (excepting [13]­cyclacene), and by a smaller but increasing amount for the even numbered belts of larger sizes. The principal effect of introducing TAO–DFT static correlation into these cyclacene geometries is therefore to make the belts thinner in the direction perpendicular to their circumference.

**Table 2:** Bond Lengths (in Å) for the [*n*]­Cyclacenes Calculated Using KS-DFT and TAO–DFT

	KS-DFT geometry			TAO–DFT geometry			ΔTAO geometry		
belt size	annulene C–C	bridging C–C	C–H	annulene C–C	bridging C–C	C–H	annulene C–C	bridging C–C	C–H
[6]cyclacene	1.414	1.449	1.085	1.415	1.447	1.083	+0.001	–0.002	–0.002
[7]cyclacene	1.408	1.469	1.085	1.410	1.457	1.083	+0.002	–0.012	–0.002
[8]cyclacene	1.407	1.456	1.085	1.404	1.455	1.084	–0.003	–0.001	–0.001
[9]cyclacene	1.406	1.467	1.085	1.407	1.455	1.084	+0.001	–0.012	–0.001
[10]cyclacene	1.404	1.460	1.085	1.404	1.456	1.084	0.000	–0.004	–0.001
[11]cyclacene	1.404^a^	1.467	1.085	1.404	1.455	1.084	0.000	–0.012	–0.001
[12]cyclacene	1.402	1.462	1.085	1.403	1.457	1.084	+0.001	–0.005	–0.001
[13]cyclacene	1.404^a^	1.460	1.085	1.403	1.456	1.084	–0.001	–0.004	–0.001
[14]cyclacene	1.403	1.464	1.085	1.403	1.457	1.084	0.000	–0.007	–0.001

a These bond lengths are averages due to lowered symmetry.

While these geometric changes are relatively small on atomic length scales, or when compared to the belt diameters shown in Figure 3, they can still induce significant changes in the electronic energies, which have been isolated and reported in Table 3. Because all of the energies here have been calculated using TAO–DFT at the same level of theory, any differences in these energies can be attributed directly to changes in the nuclear geometry. The vertical singlet–triplet excitation energies are predicted to be uniformly smaller using when TAO–DFT geometries instead of the equivalent KS-DFT geometries, and these energy changes are consistently larger for the triplet excited state energy components compared to the singlet ground state components. This indicates that static correlation becomes even more important when describing the triplet excited states. The largest energy changes when excluding [11]­cyclacene and [13]­cyclacene are seen for the triplet states of [7]­cyclacene and [9]­cyclacene, which change by ca. 0.05 eV.

**Figure 3:**
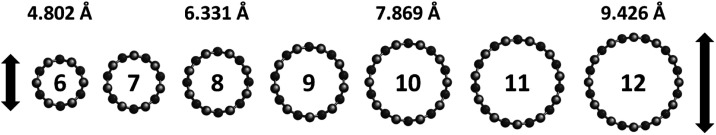
Calculated [*n*]­cyclacene TAO–DFT geometries with select belt diameters.

**Table 3:** Vertical Singlet-Triplet TAO–DFT Excitation and State Energy Changes (in eV) for the [*n*]­Cyclacenes with KS-DFT and TAO–DFT Geometries Optimized in the Ground State

belt size	KS-DFT geometry excitations	TAO–DFT geometry excitations	ΔTAO geometry excitations	ΔTAO S_0_ state energies	ΔTAO T_1_ state energies
[6]cyclacene	0.36	0.34	–0.02	–0.02	–0.04
[7]cyclacene	0.24	0.22	–0.02	–0.03	–0.05
[8]cyclacene	0.35	0.34	–0.02	0.00	–0.01
[9]cyclacene	0.14	0.13	–0.01	–0.04	–0.05
[10]cyclacene	0.26	0.25	–0.01	0.00	–0.01
[11]cyclacene	0.13	0.12	–0.02	–0.13	–0.15
[12]cyclacene	0.20	0.18	–0.02	–0.03	–0.04
[13]cyclacene	0.14	0.12	–0.02	–0.30	–0.32
[14]cyclacene	0.14	0.13	–0.01	–0.01	–0.02

### Strain Energy Changes

The energy changes induced when using these different geometries are expected to arise in part from the differing ring strain energies predicted by TAO–DFT and KS-DFT. As ring strain is a possible cause of cyclacene instability under experimental conditions, these ring strain energies have been quantified using the extrapolation scheme proposed by Hopf and co-workers.^10,22,23,59^ This method works due to the bending strain energy of a perfect ring being proportional to the inverse square of the ring’s radius, and as the radii of the cyclacene belts are proportional to the number of benzenoid fragments that make up the belts. The electronic energy of the [n]­cyclacenes divided by *n* is therefore an approximately linear function of *n* ^–2^, and the energy at the “y-intercept” of this linear function, i.e., where *n* ^–2^ = 0, represents the energy of a hypothetical strain-free C_4_H_2_ benzenoid unit in an infinitely large belt. The strain energy of each cyclacene can then be approximated from the difference between the total [n]­cyclacene energy and the energy of *n* strain-free fragments as

3 $$\Delta E_{\mathrm{strain}}=E_{\left[n\right]\mathrm{cyclacene}}-nE_{C_{4}H_{2}}$$

Using this method, Gupta et al. determined the energy of the strain-free benzenoid fragment to be ca. −96,287 kcal/mol at the TAO-PBE/6–31G­(d)//TAO-LDA/6–31G­(d) level of theory.^10^ Here the strain-free fragment energy is found to be ca. −96,442.1 and −96,442.3 kcal/mol for the TAO–DFT and KS-DFT geometries, respectively. The associated ground state strain energies are shown in Table 4, and the graphs used for the linear fitting are available in the Supporting Information. TAO–DFT geometries lead to larger ring strains for all belt sizes (except the symmetry lowered cases), and larger strain energy changes are seen for the even numbered belt sizes compared to the odd numbered belts. [6]­cyclacene has a ring strain energy ca. 53.8 kcal/mol larger than [8]­cyclacene, and can be considered ca. 35% more strained. Ring strain energy also drops rapidly for the larger belts, and the [12]­cyclacene belt has less than half the strain energy of [6]­cyclacene. These results show that using TAO–DFT for the geometry calculations increases the ring strain while also lowering the belts singlet–triplet gaps, revealing the cyclacenes to be less stable on both fronts. Strain energies have also been calculated for the cyclacenes in their vertical T_1_ triplet states, shown in Table 5. While the triplet state ΔTAO strain energy changes are smaller than in the ground state, the absolute strain energies are higher by up to ca. 8.0 kcal/mol for [8]­cyclacene and by ca. 7.9 kcal/mol for [6]­cyclacene. The triplet state strain-free fragment energies match the ground state strain-free energies to within one decimal place, and the higher finite belt strain energies indicate that triplet electronic excitations can further destabilize cyclacenes by inducing an even more strained electronic configuration.

**Table 4:** Ground State (S_0_) TAO–DFT Strain Energies for [*n*]­Cyclacenes

	KS-DFT geometry strain energy		TAO–DFT geometry strain energy		ΔTAO geometry strain energy	
belt size	(kcal/mol)	(eV)	(kcal/mol)	(eV)	(kcal/mol)	(eV)
[6]cyclacene	206.7	8.96	207.7	9.01	1.0	0.04
[7]cyclacene	185.3	8.03	186.1	8.07	0.8	0.04
[8]cyclacene	152.1	6.59	153.9	6.67	1.8	0.08
[9]cyclacene	142.3	6.17	143.4	6.22	1.1	0.05
[10]cyclacene	121.9	5.29	124.0	5.38	2.1	0.09
[11]cyclacene	116.3	5.04	115.6	5.01	–0.6	–0.03
[12]cyclacene	101.6	4.40	103.6	4.49	2.1	0.09
[13]cyclacene	100.9	4.38	96.8	4.20	–4.1	–0.18
[14]cyclacene	86.5	3.75	89.4	3.87	2.8	0.12

**Table 5:** Vertical Triplet State (T_1_) TAO–DFT Strain Energies for [*n*]­Cyclacenes

	KS-DFT geometry strain energy		TAO–DFT geometry strain energy		ΔTAO geometry strain energy	
belt size	(kcal/mol)	(eV)	(kcal/mol)	(eV)	(kcal/mol)	(eV)
[6]cyclacene	215.2	9.33	215.6	9.35	0.4	0.02
[7]cyclacene	190.9	8.28	191.2	8.29	0.4	0.02
[8]cyclacene	160.4	6.96	161.9	7.02	1.5	0.06
[9]cyclacene	145.7	6.32	146.6	6.36	0.9	0.04
[10]cyclacene	128.2	5.56	130.1	5.64	1.9	0.08
[11]cyclacene	119.6	5.19	118.6	5.14	–1.0	–0.04
[12]cyclacene	106.4	4.61	108.1	4.69	1.7	0.07
[13]cyclacene	104.4	4.53	99.9	4.33	–4.6	–0.20
[14]cyclacene	90.1	3.91	92.8	4.03	2.7	0.12

### Adiabatic Excitations and Zero-Point Energies

One advantage of using TAO–DFT to calculate cyclacene geometries and vibrational frequencies is that vibrational zero-point energy (ZPE) calculations also include static electron correlation effects that are otherwise absent from KS-DFT calculations.^35^ Harmonic ZPEs and ΔTAO values are shown for the ground state and the lowest triplet electronic state configurations in Table 6. These ZPEs include contributions from all of the molecules vibrational frequencies, as they include summations of harmonic frequencies

4 $$\mathrm{ZPE}=\frac{1}{2}\sum_{i=1}^{m}\omega_{i}$$

where the summation runs over all the harmonic vibrational frequencies associated with each cyclacene molecule. These ZPEs change in an approximately linear fashion with belt size, due to being dominated by the greater number of atoms and vibrational degrees of freedom that are present in each of the larger molecules. However, vibrational cryptoannulenic effects are still present in these vibrational energies, and the fluctuations between the odd and even belt sizes can be seen more clearly when comparing the ZPEs per benzenoid subunit in Figure 4. The singlet state TAO–DFT ZPEs show a smoother increasing pattern than the equivalent KS-DFT ZPEs, leading to erratic ΔTAO shifts in the ZPE ranging between −1.638 and +0.315 kcal/mol for [6]­cyclacene and [9]­cyclacene, respectively. In contrast to this, the TAO–DFT changes become monotonically increasing for the triplet states. However, comparison with KS-DFT is severally limited in the triplet state by the prevalence of symmetry breaking in the geometries of all of the odd belt sizes.

**Figure 4:**
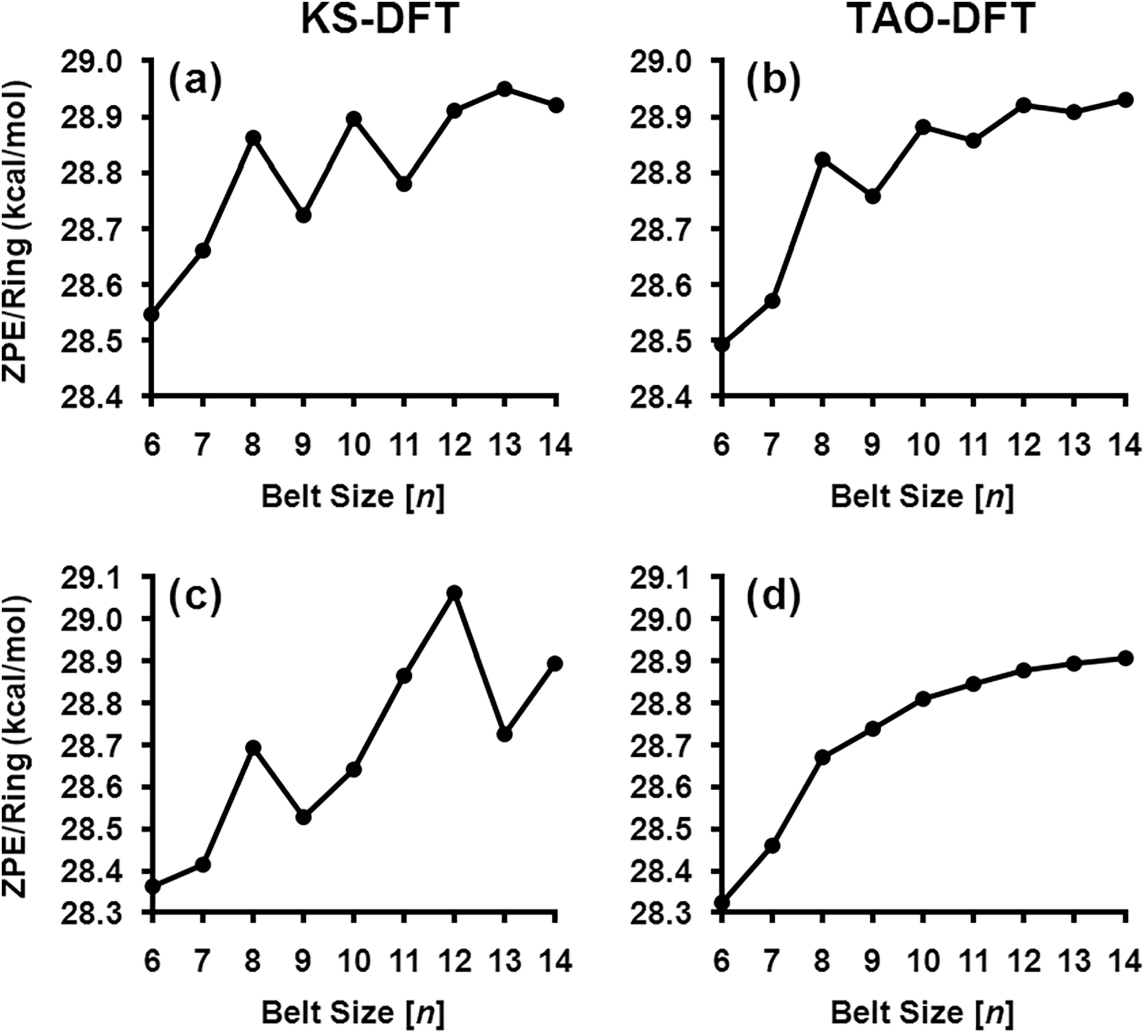
Cyclacene vibrational zero-point energies per benzenoid in their (a) KS-DFT ground states, (b) TAO–DFT ground states, (c) KS-DFT triplet states, and (d) TAO–DFT triplet states.

**Table 6:** Vibrational Zero-Point Energies (in kcal/mol) for the Calculated [*n*]­Cyclacene Geometries in Both Their Singlet Electronic Ground States and Lowest Triplet Excited States

	ground state vibrational ZPE			triplet state vibrational ZPE		
belt size	KS-DFT ZPE	TAO–DFT ZPE	ΔTAO ZPE	KS-DFT ZPE	TAO–DFT ZPE	ΔTAO ZPE
[6]cyclacene	171.282	170.961	–0.321	170.178	169.936	–0.242
[7]cyclacene	200.629	200.006	–0.623	198.902	199.216	0.314
[8]cyclacene	230.910	230.598	–0.312	229.553	229.375	–0.178
[9]cyclacene	258.520	258.835	+0.315	256.755	258.635	1.880
[10]cyclacene	288.957	288.814	–0.143	286.407	288.111	1.704
[11]cyclacene	316.580	317.447	+0.867	317.503	317.290	–0.213
[12]cyclacene	346.927	347.055	+0.128	348.741	346.533	–2.208
[13]cyclacene	376.341	375.814	–0.527	373.428	375.621	2.193
[14]cyclacene	404.886	405.026	+0.140	404.515	404.704	0.189

Changes in the calculated vibrational frequencies also lead to corresponding changes in the adiabatic singlet–triplet excitation energies shown in Table 7. TAO–DFT ZPE corrections are found to consistently reduce the adiabatic excitation energies, with the largest reductions seen for the [6]­cyclacene and [8]­cyclacene belts, which are lowered by ca. 0.04 and 0.05 eV, respectively. ZPE corrections also get progressively smaller for the larger belts, which can be rationalized as being due to the smaller singlet–triplet gaps indicating smaller electronic structure changes that in turn lead to smaller geometric and vibrational changes. The KS-DFT adiabatic excitations are so distorted by lowered geometric symmetry that the [13]­cyclacene belt is predicted to have a triplet adiabatic ground state. This is due to both the singlet and triplet KS-DFT geometries suffering from significant distortions, and calculations using TAO–DFT geometries show the expected singlet ground state with annulene bonds of equal length in both states. TAO–DFT also predicts a singlet state energy that is both ca. 0.11–0.12 eV below the lowest triplet state and ca. 0.38 eV lower than predicted using KS-DFT. The [8]­cyclacene and [6]­cyclacene belts again have the largest singlet–triplet excitation energies overall at 0.31 and 0.30 eV, respectively.

**Table 7:** TAO–DFT Adiabatic Singlet-Triplet Excitation Energies (in eV) for the [*n*]­Cyclacenes Including and Excluding Vibrational Zero-Point Energy Corrections

	adiabatic excitations (excl. ZPE)			adiabatic excitations (incl. ZPE)		
belt size	KS geometry	TAO geometry	ΔTAO geometry	KS geometry	TAO geometry	ΔTAO geometry
[6]cyclacene	0.36	0.34	–0.02	0.31	0.30	–0.02
[7]cyclacene	0.33	0.21	–0.11	0.25	0.18	–0.07
[8]cyclacene	0.36	0.36	0.00	0.30	0.31	+0.01
[9]cyclacene	0.22	0.13	–0.09	0.14	0.12	–0.02
[10]cyclacene	0.28	0.25	–0.03	0.17	0.22	+0.06
[11]cyclacene	0.03	0.12	+0.09	0.07	0.11	+0.04
[12]cyclacene	0.20	0.19	–0.01	0.28	0.17	–0.11
[13]cyclacene	–0.14	0.12	+0.26	–0.27	0.11	+0.38
[14]cyclacene	0.16	0.13	–0.03	0.15	0.12	–0.03

### Individual Vibrational Shifts

One method for quantifying the degree of static electron correlation present in each vibrational mode is to extend the ΔTAO method to directly compare the changes between the calculated KS-DFT and TAO–DFT vibrational frequencies^42,43^

5 $$\Delta {\mathrm{TAO}}_{\omega }=\omega_{\mathrm{TAO}-\mathrm{DFT}}-\omega_{\mathrm{KS}-\mathrm{DFT}}$$

This approach is relatively simple, and has been shown to correlate well with more advanced wave function based approaches for quantifying the vibrational effects of static correlation, such as measuring the second derivatives of the symmetrized von Neumann entropy with respect to normal mode displacements.^43^ However, ΔTAO frequency shifts represent distinct methodological differences in nuclear potential energy surface curvature, nuclear geometries, and vibrational normal mode coordinates, all of which contribute to vibrational changes. Vibrational frequencies have been compared between the KS-DFT and TAO–DFT methods, and ΔTAO shifts are found to be significant for many of the cyclacene vibrational modes, with absolute shifts ranging between ca. 7 and 11 cm^–1^ per mode at the different belt sizes (see Supporting Information). These ΔTAO values were calculated by matching the mode frequencies based on their energy ordering, and the ΔTAO shifts may be larger when accounting for reordering. Key modes with particularly high ΔTAO shifts are shown in Table 8 and Figure 5, and the first vibrational modes with a frequency above 1000 cm^–1^ in each belt are found to have particularly large ΔTAO shifts. These modes involve combinations of in-phase C–H wagging motion parallel to the ring system with C–C bending motion within the ring system. The vibrational frequencies of these modes are shifted by ca. 70, 136, 65, 203, 81, 215, 109, 32, and 162 cm^–1^ for the increasing belt sizes, respectively, which corresponds to a change of between ca. 6% and 22% of the total mode energy when excluding [13]­cyclacene. In the case of [13]­cyclacene the corresponding KS-DFT mode is ca. 32 cm^–1^ higher in energy than the TAO–DFT mode, and the corresponding normal mode coordinate is found to be qualitatively different in the KS-DFT case. The next three highest frequency modes of all of the belts involve similar combinations of C–H wagging and C–C bending components, but with smaller ΔTAO shifts. The largest of these is seen for the next highest energy mode in [9]­cyclacene, with a ΔTAO shift of ca. 121 cm^–1^, or ca. 12% of the total transition energy. The differences in ΔTAO shifts seen for the displacement of similar atoms in Figure 5 (representative of both methods) indicate that these vibrational changes are likely due to more subtle variations in the energy rather than qualitative changes in the normal coordinates. The magnitude of the ΔTAO shifts show that static electron correlation plays an important role in cyclacene dynamics and that it is necessary to account for static correlation in order to properly describe the vibrational dynamics and associated properties of cyclacenes.

**Figure 5:**
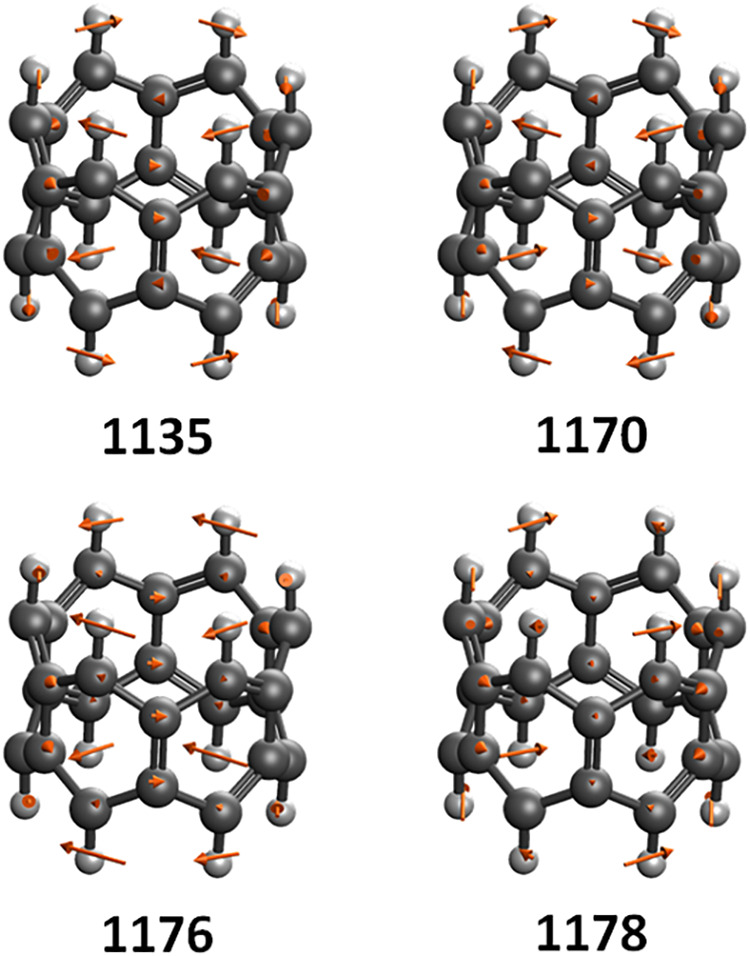
Representative TAO–DFT vibrational normal mode arrow vectors for the first four vibrational modes of [6]­cyclacene above 1000 cm^–1^. Frequencies are given below in cm^–1^.

**Table 8:** Harmonic Vibrational Frequencies of the First [*n*]­Cyclacene Modes above 1000 cm^–1^

belt size	KS-DFT (cm^–1^)	TAO–DFT (cm^–1^)	ΔTAO (cm^–1^)	ΔTAO (%)
[6]cyclacene	1064.43	1134.87	+70.44	+6.6
	1175.46	1169.92	–5.54	–0.5
	1176.20	1175.53	–0.67	–0.1
	1189.21	1178.36	–10.85	–0.9
[7]cyclacene	971.13	1107.11	+135.98	+14.0
	1038.35	1127.94	+89.59	+8.6
	1190.23	1150.59	–39.64	–3.3
	1195.39	1155.34	–40.05	–3.4
[8]cyclacene	1085.46	1150.45	+64.99	+6.0
	1167.18	1175.94	+8.76	+0.8
	1167.26	1175.97	+8.71	+0.7
	1202.07	1178.23	–23.84	–2.0
[9]cyclacene	923.42	1126.19	+202.77	+22.0
	1026.33	1147.55	+121.22	+11.8
	1170.64	1148.06	–22.58	–1.9
	1171.60	1157.39	–14.21	–1.2
[10]cyclacene	1065.88	1146.70	+80.82	+7.6
	1138.53	1164.68	+26.15	+2.3
	1139.91	1166.81	+26.90	+2.4
	1194.49	1172.97	–21.52	–1.8
[11]cyclacene	923.64	1138.43	+214.79	+23.3
	1092.70	1156.81	+64.11	+5.9
	1159.50	1157.32	–2.18	–0.2
	1159.70	1169.23	+9.53	+0.8
[12]cyclacene	1035.51	1144.86	+109.35	+10.6
	1114.37	1162.89	+48.52	+4.4
	1114.84	1162.94	+48.10	+4.3
	1190.00	1171.84	–18.16	–1.5
[13]cyclacene	1175.87	1144.37	–31.50	–2.7
	1176.01	1163.63	–12.38	–1.1
	1179.11	1163.85	–15.26	–1.3
	1182.47	1174.02	–8.45	–0.7
[14]cyclacene	984.29	1146.37	+162.08	+16.5
	1085.80	1164.66	+78.86	+7.3
	1087.09	1166.07	+78.98	+7.3
	1179.96	1173.99	–5.97	–0.5

### Vibrational Stabilization of Electronic Excitations

While ΔTAO frequency shifts have been shown to correlate with significant changes in the electron radical character along the normal coordinates of smaller molecules,^43^ larger frequency shifts can also be due to geometric changes in both the origin of the normal coordinates and the coordinates themselves. Given the impact that the singlet–triplet excitation energies are expected to have on cyclacene stabilities under experimental conditions, the extent to which these vibrations can stabilize or destabilize the electronic transitions has been investigated. After all, molecules are not localized at their classical equilibrium geometries, but instead show quantum mechanical nuclear behavior, with wave functions extending along the normal coordinates. Even in vibrational ground states, the expectation values of the nuclei are shifted away from the equilibrium positions due to vibrational anharmonicity and associated asymmetry in their nuclear wave functions.^60^ Changes in electronic stabilities can also be reasonably expected to amplify with vibrational excitations, and these effects differ from the vibrational zero-point energy corrections discussed previously. One way of approximating these nuclear wave function effects is to consider the electronic excitations at the classical turning points (CTPs) of the harmonic oscillators that define them. The CTPs of a quantum harmonic oscillator are the coordinates at which the quantum vibrational energy would become entirely potential energy if the oscillators were classical instead of quantum mechanical. Vertical singlet–triplet electronic excitation energies have been calculated for the four smallest [*n*]­cyclacenes (*n* = 6–9) at both the positive and negative CTP displacements (+CTP/-CTP), and in both the vibrational ground state and the first excited vibrational states of each oscillator. This has been done for all modes showing an absolute ΔTAO frequency shift greater than 10 cm^–1^ and an absolute frequency less than 3000 cm^–1^. Vibrational displacements inducing singlet–triplet transition energy shifts greater than 0.01 eV are shown in Table 9, and a complete set of energies is given in the Supporting Information.

**Table 9:** Singlet-Triplet Vertical Transition Energy Changes for [6]­Cyclacene to [9]­Cyclacene at the Classical Vibrational Turning Points of Select Modes

			ground state (v_0_) Δ*E*_ST_ (eV)			excited state (v_1_) Δ*E*_ST_ (eV)		
belt size	mode	frequency (cm^–1^)	+CTP	–CTP	mean	+CTP	–CTP	mean
[6]cyclacene	55	1134.87	0.00	0.00	0.00	+0.01	+0.01	+0.01
	80	1409.03	+0.01	+0.01	+0.01	+0.02	+0.02	+0.02
	86	1511.16	+0.02	–0.02	0.00	+0.03	–0.03	0.00
	89	1521.34	–0.01	–0.01	–0.01	–0.02	–0.02	–0.02
	90	1521.58	–0.01	–0.01	–0.01	–0.02	–0.02	–0.02
[7]cyclacene	16	403.35	+0.01	–0.01	0.00	+0.03	–0.02	0.00
	65	1107.11	+0.01	+0.01	+0.01	+0.02	+0.02	+0.02
	66	1127.94	0.00	0.00	0.00	+0.01	+0.01	+0.01
	67	1150.59	0.00	0.00	0.00	–0.01	–0.01	–0.01
	68	1155.34	0.00	0.00	0.00	–0.01	–0.01	–0.01
	90	1372.66	0.00	0.00	0.00	–0.01	–0.01	–0.01
	93	1384.21	+0.01	+0.01	+0.01	+0.02	+0.02	+0.02
[8]cyclacene	75	1150.45	+0.01	+0.01	+0.01	+0.01	+0.01	+0.01
	117	1541.19	–0.01	–0.01	–0.01	–0.02	–0.02	–0.02
	118	1541.20	–0.01	–0.01	–0.01	–0.02	–0.02	–0.02
[9]cyclacene	85	1126.19	0.00	0.00	0.00	+0.01	+0.01	+0.01

The results indicate that only a handful of vibrational modes cause significant changes in the electronic excitations, and that these modes appear more prevalent at the smaller belt sizes. However, out of the vibrations that cause significant changes in the electronic excitation energies, shifts of up to ±0.01 eV persist in the vibrational ground state. Despite these more prominent shifts, the average excitation energy change over all of the modes still nets out to roughly zero in the vibrational ground states of all four belts, and vibrationally excited states that allow individual oscillator modes to be excited are therefore of more interest. Modes involving predominantly C–C ring stretching motion in the spectral region between 1000 and 1600 cm^–1^ have the most consistent impact on the cyclacene excitations, which is also consistent with the HOMO and LUMO being localized on the outer annulene carbon atoms. These C–C ring stretching modes induce electronic shifts of up to ca. 0.02 eV at the classical turning points.

The vibrational modes at ca. 1511 cm^–1^ in [6]­cyclacene and at ca. 403 cm^–1^ in [7]­cyclacene induce significant singlet–triplet excitation energy changes of up to ± 0.02 and ± 0.03 eV, respectively. However, these modes both stabilize and destabilize the transitions depending on which way the atoms are displaced, and the average transition energy change is roughly zero. Anharmonic vibrational wave function analysis is therefore necessary to properly account for the vibrational asymmetry in these types of mode and assess their impact. The mode with a frequency of ca. 403 cm^–1^ in [7]­cyclacene is an exception to this, however, as it represents the symmetric ring breathing mode. This breathing mode has a ca. 21 cm^–1^ absolute ΔTAO shift, which can be compared to 2, 1, and 3 cm^–1^ shifts for the symmetric breathing modes of the *n* = 6, 8, and 9 belts at ca. 454, 362, and 324 cm^–1^, respectively, indicating that the singlet–triplet transition in [7]­cyclacene is particularly sensitive to the ring breathing motion. The normal mode atomic displacements for this mode point inward such that the +CTP displacement causes the belt to contract while the −CTP displacement causes the belt to expand. Because the symmetric ring breathing mode directly expands and contracts the belt diameter in a uniform manner without introducing further distortions into the nuclear geometry, the ring strain energy changes for this mode can be calculated by substituting the electronic energies at the CTPs into eq 3. Both positive and negative displacements increase the overall ring strain by between ca. 0.2 and 1.0 kcal/mol in the ground state, and by between ca. 1.0 and 2.4 kcal/mol in the first vibrationally excited state. However, the belt contraction increases the strain by less than the belt expansion, and when expanding, the singlet state strain increases more than in the triplet state. The opposite is also true for the contraction. These strain energy changes match the electronic transition energy changes to within 0.01 eV, indicating that changes in the electronic transition energy are largely a function of the ring strain differences between the singlet and triplet states. Furthermore, as the ring strain is greater in the ground state at the −CTP relative to the +CTP, it is more likely that wave function anharmonicity will favor the higher electronic transition energies for this symmetric breathing mode.

### Infrared Spectroscopy Changes

The cyclacene vibrational modes discussed so far have not been significantly IR active. The IR spectra for these cyclacenes have been calculated using both KS-DFT and TAO–DFT, and are shown in Figure 7, 8, 9, 10 in addition to the Supporting Information. These spectra are dominated by two intense IR reporter bands in the spectral region between 500 and 1000 cm^–1^, particularly for the smaller [6]­cyclacene and [7]­cyclacene belts where the weaker of the two bands is still relatively prominent. The two reporter bands correspond to C–H wagging motions perpendicular to the ring, with the lower energy of the two consisting of a single vibrational mode that also involves significant carbon atom wagging motion, and with the higher frequency band being a combination of primarily three vibrational modes with more C–H wagging motion. All four of these reporter modes are shown for [6]­cyclacene in Figure 6. The lowest intensity of the four bands gets weaker at larger belt sizes to the point that only a single band with mostly C–H wagging dominates the midrange spectrum for [8]­cyclacene and above. A handful of other low intensity bands can be seen in this spectral region, but not much is visible before the stretching region of the spectrum above 3000 cm^–1^ (see Supporting Information). The IR frequencies, approximate transition intensities, and ΔTAO shifts of these reported modes are shown in Table 10, where the ΔTAO shifts have been calculated by visually matching between the normal coordinate displacements instead of relying on energy ordering. The key C–H wagging reporter IR band frequencies are found to have frequency shifts of up to ca. 34 cm^–1^. These differences lead to qualitative changes in the IR spectra for the odd belt sizes calculated using KS-DFT and TAO–DFT, with peak splitting observed when using KS-DFT even under significant Gaussian broadening. Static correlation is therefore recommended for accurately simulating the IR spectra of these cyclacene belts in addition to their other dynamic quantities. Raman spectra have also been calculated using both methods for completeness, and show more complex spectral shifts, which can be found in the Supporting Information.

**Figure 6:**
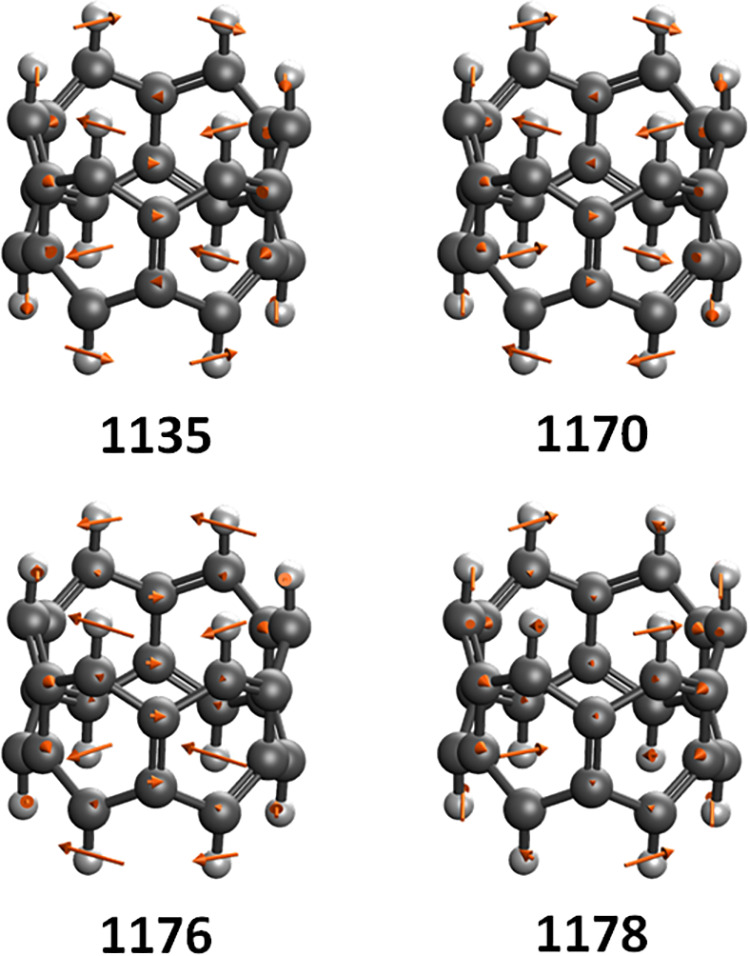
Representative TAO–DFT vibrational normal mode arrow vectors for the IR reporter modes of [6]­cyclacene. Mode frequencies are given below in cm^–1^.

**Figure 7:**
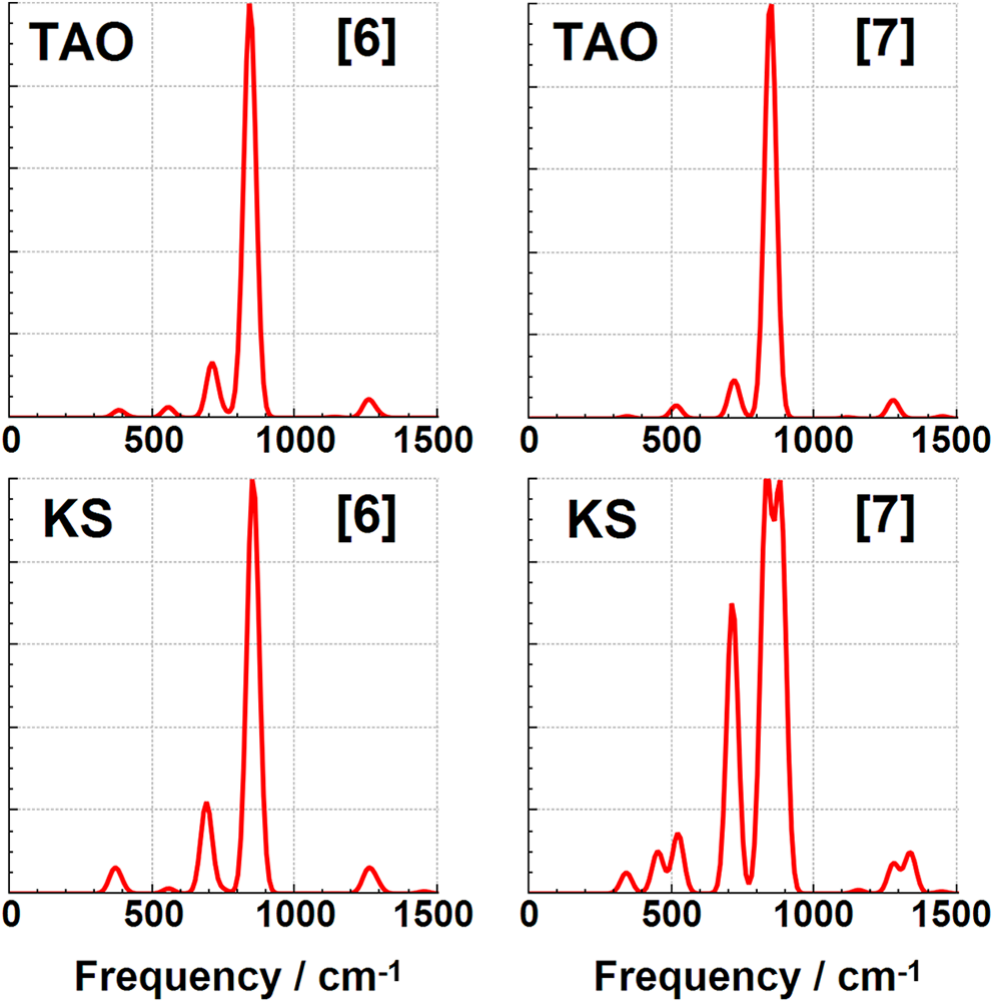
Infrared TAO–DFT and KS-DFT spectra for [6]­cyclacene and [7]­cyclacene.

**Figure 8:**
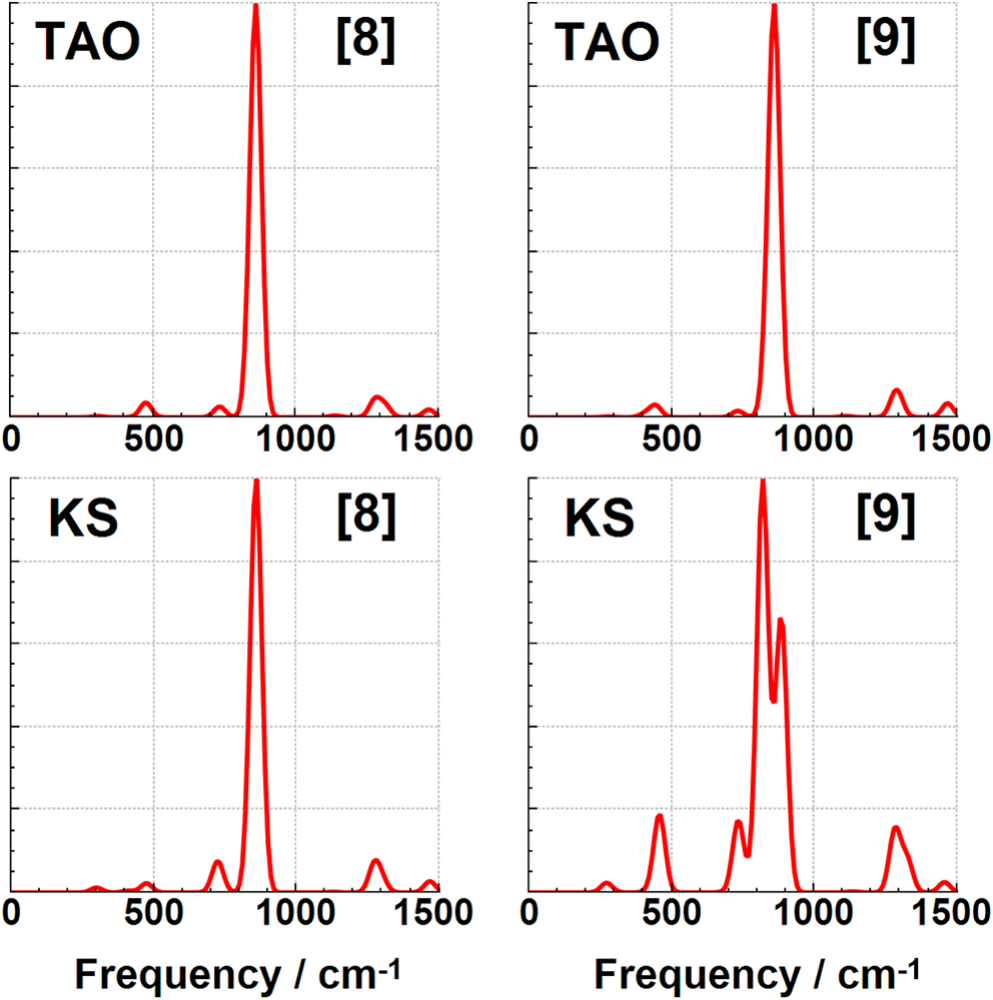
Infrared TAO–DFT and KS-DFT spectra for [8]­cyclacene and [9]­cyclacene.

**Figure 9:**
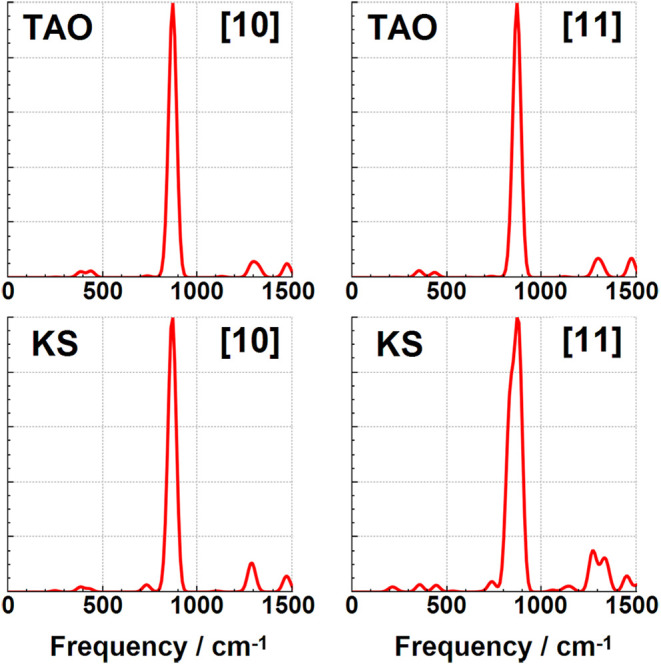
Infrared TAO–DFT and KS-DFT spectra for [10]­cyclacene and [11]­cyclacene.

**Figure 10:**
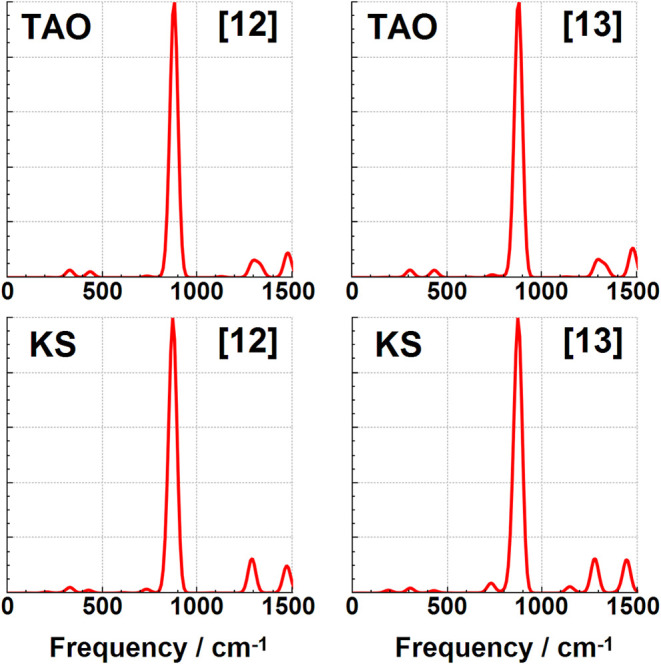
Infrared TAO–DFT and KS-DFT spectra for [12]­cyclacene and [13]­cyclacene.

**Table 10:** Select Harmonic IR Reporter Band Frequencies and Intensities for the Intense Vibrational Modes of the [*n*]­Cyclacenes between 500–1000 cm^–1^

	KS-DFT		TAO–DFT		ΔTAO		
belt size	frequency (cm^–1^)	intensity (km/mol)	frequency (cm^–1^)	intensity (km/mol)	frequency (cm^–1^)	intensity (km/mol)	frequency (%)
[6]cyclacene	712.27	390.992	732.24	178.427	+19.97	–212.565	+2.8
	874.24	649.284	860.57	516.892	–13.67	–132.392	–1.6
	879.01	664.765	872.67	491.347	–6.34	–173.418	–0.7
	890.81	308.270	877.40	408.932	–13.41	+100.662	–1.5
[7]cyclacene	736.26	375.066	741.89	136.404	+5.63	–238.662	+0.8
	858.71	507.851	868.32	434.610	+9.61	–73.241	+1.1
	906.51	184.449	872.18	572.591	–34.33	+388.142	–3.8
	914.63	244.877	880.21	566.347	–34.42	+321.470	–3.8
[8]cyclacene	747.42	167.261	757.24	43.808	+9.82	–123.453	+1.3
	878.51	662.929	879.85	454.617	+1.34	–208.312	+0.2
	889.30	805.213	890.26	660.572	+0.96	–144.641	+0.1
	889.51	799.124	890.34	652.448	+0.83	–146.676	+0.1
[9]cyclacene	758.00	148.501	755.76	20.447	–2.24	–128.054	–0.3
	845.71	822.362	870.43	301.304	+24.72	–521.058	+2.9
	912.82	169.685	889.26	730.268	–23.56	+560.583	–2.6
	914.56	256.206	892.18	758.749	–22.38	+502.543	–2.4
[10]cyclacene	755.24	65.035	759.85	9.544	+4.61	–55.491	+0.6
	880.67	559.683	884.81	346.643	+4.14	–213.040	+0.5
	897.74	1005.477	899.27	765.969	+1.53	–239.508	+0.2
	900.74	1002.410	902.34	766.073	+1.60	–236.337	+0.2
[11]cyclacene	762.28	21.645	758.04	4.383	–4.24	–17.262	–0.6
	856.08	669.290	881.09	217.382	+25.01	–451.908	+2.9
	907.66	810.097	901.12	856.977	–6.54	+46.88	–0.7
	908.70	708.463	902.07	793.126	–6.63	+84.663	–0.7
[12]cyclacene	754.88	29.682	758.86	2.703	+3.98	–26.979	+0.5
	880.35	624.277	890.75	202.766	+10.40	–421.511	+1.2
	903.32	1135.787	908.54	855.381	+5.22	–280.406	+0.6
	903.34	1153.933	908.64	835.682	+5.03	–318.251	+0.6
[13]cyclacene	750.68	68.992	756.84	1.135	+6.16	–67.857	+0.8
	879.66	811.795	887.25	204.495	+7.59	–607.300	+0.9
	903.50	884.771	907.37	942.996	+3.87	+58.225	+0.4
	904.87	1185.661	907.83	835.324	+2.96	–350.337	+0.3
[14]cyclacene	754.30	6.308	756.48	0.903	+2.18	–5.405	+0.3
	885.49	604.664	892.15	264.546	+6.66	–340.118	+0.8
	909.12	1404.153	908.88	988.348	–0.24	–415.805	–0.0
	912.06	1351.118	911.24	926.198	–0.82	–424.920	–0.1

## Conclusions

Using TAO–DFT to calculate cyclacene geometries leads to a reduction in the lowest cyclacene singlet–triplet excitation energies, and to an increase in their calculated ring strain energies compared to using KS-DFT geometries. These differences are due to geometric changes between the methods. Ring strain energies are higher in the triplet electronic states than in the singlet ground states, and TAO–DFT geometry changes induce energetic shifts in the adiabatic singlet–triplet excitation energies of up to ca. 41%. TAO–DFT shortens the C–C bonds that bridge between the two annulene ribbons of the odd numbered cyclacenes and makes the belts thinner in the direction perpendicular to their circumference. Overall, [8]­cyclacene is still predicted to have the largest vertical and adiabatic singlet–triplet excitation energy of the molecules tested when using TAO–DFT throughout, and to be the most stable cyclacene in this respect. However, [6]­cyclacene is predicted to have a roughly equivalent vertical excitation energy within 0.0005 eV, and an adiabatic transition ca. 0.01 eV lower than [8]­cyclacene. Qualitative vibrational changes are observed in the infrared spectra of the odd sized belts, with vibrational peak splitting seen when using KS-DFT. Individual vibrational modes are also shifted by up to 203 cm^–1^ in the TAO–DFT calculations, indicating that static correlation is important for accurately simulating the vibrational and dynamic properties of cyclacenes. Classical turning point analysis indicates that vibrational mode extensions can stabilize and destabilize the singlet–triplet excitations along certain vibrational modes, particularly for the ring carbon stretching vibrations of [6]­cyclacene and [7]­cyclacene, and the [7]­cyclacene singlet–triplet transition is found to be particularly affected by ring strain changes during the symmetric ring breathing mode. These results suggest that selective vibrational quenching and excitation may enhance cyclacene stability under experimental conditions.

## Supplementary Material



## Notes

Calculated absolute energies, vibrational frequencies, vibrational zero-point energies, infrared spectra, and atomic Cartesian coordinates (PDF)
