# Supplementary material for: Vibrational Stabilization in Cyclacene Carbon Nanobelts
Source: J Phys Chem A. 2025 Sep 4;129(37):8601–12. doi: 10.1021/acs.jpca.5c04863 (PMC12451658; doi:10.1021/acs.jpca.5c04863)
Supplement: Supplementary file 1 [file jp5c04863_si_001.pdf]

Vibrational Stabilization in Cyclacene Carbon Nanobelts: Supporting Information

Magnus W. D. Hanson-Heine*

School of Chemistry and Chemical Engineering, University of Southampton, Highfield, Southampton SO17 1BE, UK.

**magnus.hansonheine@soton.ac.uk*

Contents

1. **Absolute Energy Calculations** 2
2. **Strain Energy Calculations** 3
3. **Classical Vibrational Turning Point Energies** 4
4. **Infrared Spectroscopy Simulations** 12
5. **Raman Spectroscopy Simulations** 18
6. **Nuclear Vibrational Frequencies** 24
7. **Atomic Cartesian Coordinates** 59
   - **7.1 TAO-DFT Ground States** 59
   - **7.2 KS-DFT Ground States** 83
   - **7.3 TAO-DFT Triplet States** 106
   - **7.4 KS-DFT Triplet States** 130

1. Absolute Energy Calculations

Table S1. TAO-B3LYP-D3M(BJ)/aug-cc-pVTZ energies in E_h for the [n]cyclacene nanobelt geometries optimized using TAO-B3LYP-D3M(BJ)/6-311G(d,p) in the S_0 electronic state.

Belt Size	S_0 Ground State	T_1 Excited State
[6]cyclacene	-921.826243	-921.813867
[7]cyclacene	-1075.553475	-1075.545566
[8]cyclacene	-1229.297677	-1229.285284
[9]cyclacene	-1383.007253	-1383.002582
[10]cyclacene	-1536.730974	-1536.721698
[11]cyclacene	-1690.437203	-1690.432897
[12]cyclacene	-1844.149227	-1844.142615
[13]cyclacene	-1997.852953	-1997.848590
[14]cyclacene	-2151.557688	-2151.552738

Table S2. TAO-B3LYP-D3M(BJ)/aug-cc-pVTZ energies in E_h for the [n]cyclacene nanobelt geometries optimized using KS-B3LYP-D3M(BJ)/6-311G(d,p) in the S_0 electronic state.

Belt Size	S_0 Ground State	T_1 Excited State
[6]cyclacene	-921.825669	-768.041481
[7]cyclacene	-1075.552337	-921.812391
[8]cyclacene	-1229.297747	-1075.543639
[9]cyclacene	-1383.005833	-1229.284772
[10]cyclacene	-1536.730833	-1383.000774
[11]cyclacene	-1690.432344	-1536.721148
[12]cyclacene	-1844.148262	-1690.427450
[13]cyclacene	-1997.841832	-1844.141091
[14]cyclacene	-2151.557224	-1997.836677

Table S3. TAO-B3LYP-D3M(BJ)/aug-cc-pVTZ triplet state (T_1) energies in E_h for the [n]cyclacene nanobelt geometries optimized using TAO-B3LYP-D3M(BJ)/6-311G(d,p) and KS-B3LYP-D3M(BJ)/6-311G(d,p) in the T_1 electronic state.

Belt Size	KS-DFT Geometry	TAO-DFT Geometry
[6]cyclacene	-921.812424	-921.813688
[7]cyclacene	-1075.540348	-1075.545699
[8]cyclacene	-1229.284403	-1229.284269
[9]cyclacene	-1382.997805	-1383.002591
[10]cyclacene	-1536.720704	-1536.721766
[11]cyclacene	-1690.431291	-1690.432882
[12]cyclacene	-1844.140846	-1844.142122
[13]cyclacene	-1997.847077	-1997.848627
[14]cyclacene	-2151.551265	-2151.552727

2. Strain Energy Calculations

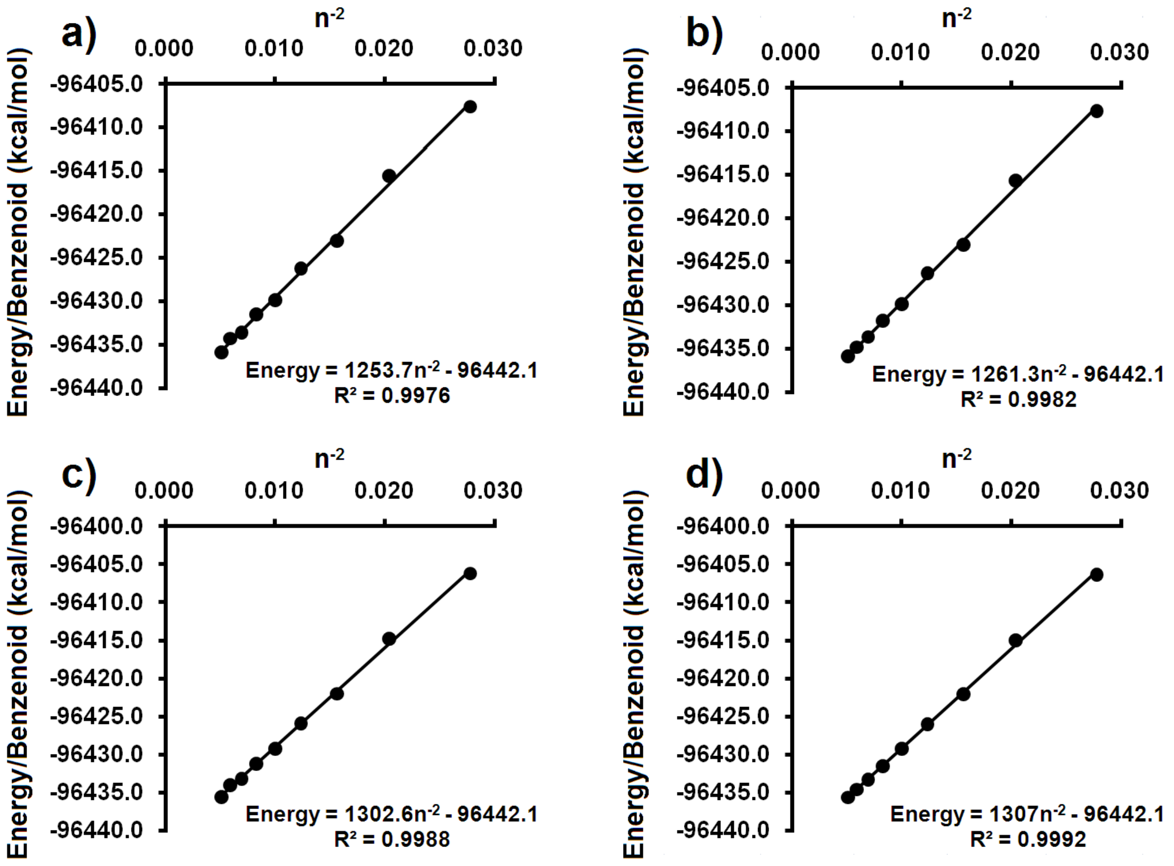

Figure S1. Strain energy linear fitting graphs showing the total electronic energy per C_4H_2 subunit plotted against n^{-2} for the [n]cyclacenes for a) the KS-DFT singlet S_0 states, b) the TAO-DFT singlet S_0 states, c) the KS-DFT triplet T_1 states, and d) the TAO-DFT triplet T_1 states.

3. Classical Vibrational Turning Point Energies

Table S4. TAO-B3LYP-D3M(BJ)/aug-cc-pVTZ ground state (S_0) and lowest triplet state (T_1) energies in E_h, calculated at the classical turning points of select TAO-B3LYP-D3M(BJ)/6-311G(d,p) [6]cyclacene vibrational normal mode oscillators in their vibrational ground states.

	Oscillator State v_0 Electronic State S_0		Oscillator State v_0 Electronic State T_1	
Mode	+CTP	-CTP	+CTP	-CTP
5	-921.825598	-921.825598	-921.813269	-921.813269
6	-921.825953	-921.825953	-921.813639	-921.813639
7	-921.825318	-921.825318	-921.812928	-921.812928
9	-921.825465	-921.825465	-921.813139	-921.813139
10	-921.825330	-921.825330	-921.812867	-921.812867
13	-921.825271	-921.825271	-921.812870	-921.812870
14	-921.825266	-921.825266	-921.812943	-921.812943
17	-921.825243	-921.825243	-921.812821	-921.812821
31	-921.824417	-921.824417	-921.811904	-921.811904
37	-921.824416	-921.824416	-921.812092	-921.812092
38	-921.824271	-921.824271	-921.811936	-921.811936
39	-921.824341	-921.824341	-921.812005	-921.812005
40	-921.824196	-921.824196	-921.811859	-921.811859
41	-921.824381	-921.824035	-921.812011	-921.811669
42	-921.824058	-921.824935	-921.811675	-921.812564
44	-921.824212	-921.824212	-921.811844	-921.811844
48	-921.824066	-921.823935	-921.811684	-921.811554
49	-921.824148	-921.824148	-921.811818	-921.811818
51	-921.824127	-921.824127	-921.811794	-921.811794
52	-921.824168	-921.824168	-921.811790	-921.811790
53	-921.823611	-921.824587	-921.811099	-921.812275
54	-921.824153	-921.824153	-921.811779	-921.811779
55	-921.823674	-921.823674	-921.811119	-921.811119
58	-921.823547	-921.823547	-921.811224	-921.811224
60	-921.823501	-921.823501	-921.811178	-921.811178
64	-921.823351	-921.823570	-921.811018	-921.811240
76	-921.823026	-921.823522	-921.810741	-921.811230
77	-921.823147	-921.823147	-921.810839	-921.810839
80	-921.823059	-921.823059	-921.810468	-921.810468
81	-921.822941	-921.822941	-921.810566	-921.810566
86	-921.823166	-921.822535	-921.810154	-921.810821
89	-921.822832	-921.822832	-921.810653	-921.810653
90	-921.822830	-921.822830	-921.810649	-921.810649

Table S5. TAO-B3LYP-D3M(BJ)/aug-cc-pVTZ ground state (S_0) and lowest triplet state (T_1) energies in E_h calculated at the classical turning points of select TAO-B3LYP-D3M(BJ)/6-311G(d,p) [6]cyclacene vibrational normal mode oscillators in their first vibrationally excited states.

	Oscillator State v_1 Electronic State S_0		Oscillator State v_1 Electronic State T_1	
Mode	+CTP	-CTP	+CTP	-CTP
5	-921.824283	-921.824283	-921.812176	-921.812176
6	-921.824539	-921.824539	-921.812377	-921.812377
7	-921.823793	-921.823793	-921.811622	-921.811622
9	-921.823903	-921.823903	-921.811635	-921.811635
10	-921.823499	-921.823499	-921.810879	-921.810879
13	-921.823320	-921.823320	-921.811108	-921.811108
14	-921.823309	-921.823309	-921.811057	-921.811057
17	-921.823241	-921.823241	-921.810736	-921.810736
31	-921.820780	-921.820780	-921.808056	-921.808056
37	-921.820721	-921.820721	-921.808515	-921.808515
38	-921.820249	-921.820249	-921.808004	-921.808004
39	-921.820496	-921.820496	-921.808245	-921.808245
40	-921.819959	-921.819959	-921.807713	-921.807713
41	-921.820230	-921.819817	-921.807922	-921.807523
42	-921.819949	-921.820318	-921.807617	-921.808008
44	-921.820109	-921.820109	-921.807767	-921.807767
48	-921.820018	-921.819843	-921.807682	-921.807511
49	-921.819847	-921.819847	-921.807617	-921.807617
51	-921.819787	-921.819787	-921.807555	-921.807555
52	-921.819985	-921.819985	-921.807612	-921.807612
53	-921.818830	-921.820762	-921.806344	-921.808539
54	-921.819960	-921.819960	-921.807599	-921.807599
55	-921.818525	-921.818525	-921.805621	-921.805621
58	-921.818154	-921.818154	-921.805957	-921.805957
60	-921.818010	-921.818010	-921.805843	-921.805843
64	-921.818075	-921.817739	-921.805860	-921.805544
76	-921.817780	-921.816974	-921.805676	-921.804864
77	-921.816944	-921.816944	-921.804783	-921.804783
80	-921.816682	-921.816682	-921.803669	-921.803669
81	-921.816324	-921.816324	-921.803953	-921.803953
86	-921.816847	-921.815314	-921.803396	-921.804110
89	-921.816011	-921.816011	-921.804217	-921.804217
90	-921.816005	-921.816005	-921.804207	-921.804207

Table S6. TAO-B3LYP-D3M(BJ)/aug-cc-pVTZ ground state (S_0) and lowest triplet state (T_1) energies in E_h calculated at the classical turning points of select TAO-B3LYP-D3M(BJ)/6-311G(d,p) [7]cyclacene vibrational normal mode oscillators in their vibrational ground states.

	Oscillator State v_0 Electronic State S_0		Oscillator State v_0 Electronic State T_1	
Mode	+CTP	-CTP	+CTP	-CTP
5	-1075.552867	-1075.552854	-1075.545042	-1075.545022
6	-1075.552854	-1075.552854	-1075.545019	-1075.545039
14	-1075.552437	-1075.552620	-1075.544638	-1075.544832
15	-1075.552510	-1075.552546	-1075.544716	-1075.544754
16	-1075.553190	-1075.551874	-1075.544744	-1075.544492
18	-1075.552433	-1075.552428	-1075.544568	-1075.544569
19	-1075.552406	-1075.552457	-1075.544597	-1075.544628
20	-1075.552403	-1075.552439	-1075.544541	-1075.544571
21	-1075.552465	-1075.552465	-1075.544503	-1075.544503
28	-1075.552058	-1075.552076	-1075.544180	-1075.544190
58	-1075.551385	-1075.551385	-1075.543470	-1075.543470
59	-1075.551349	-1075.551374	-1075.543470	-1075.543481
61	-1075.551374	-1075.551374	-1075.543495	-1075.543495
65	-1075.550970	-1075.550970	-1075.542802	-1075.542802
66	-1075.550914	-1075.550910	-1075.542881	-1075.542877
67	-1075.550884	-1075.550870	-1075.543074	-1075.543057
68	-1075.550841	-1075.550897	-1075.543032	-1075.543068
81	-1075.550594	-1075.550551	-1075.542750	-1075.542699
82	-1075.550579	-1075.550558	-1075.542724	-1075.542708
88	-1075.550381	-1075.550376	-1075.542368	-1075.542363
90	-1075.550399	-1075.550459	-1075.542625	-1075.542673
91	-1075.550439	-1075.550396	-1075.542627	-1075.542593
92	-1075.550427	-1075.550421	-1075.542609	-1075.542597
93	-1075.550315	-1075.550315	-1075.542217	-1075.542217
103	-1075.550042	-1075.550042	-1075.542243	-1075.542243
104	-1075.550032	-1075.550032	-1075.542232	-1075.542232
105	-1075.549958	-1075.549958	-1075.542082	-1075.542082
106	-1075.549962	-1075.549962	-1075.542083	-1075.542083

Table S7. TAO-B3LYP-D3M(BJ)/aug-cc-pVTZ ground state (S_0) and lowest triplet state (T_1) energies in E_h calculated at the classical turning points of select TAO-B3LYP-D3M(BJ)/6-311G(d,p) [7]cyclacene vibrational normal mode oscillators in their first vibrationally excited states.

	Oscillator State v_1 Electronic State S_0		Oscillator State v_1 Electronic State T_1	
Mode	+CTP	-CTP	+CTP	-CTP
5	-1075.551655	-1075.551639	-1075.543981	-1075.543970
6	-1075.551631	-1075.551644	-1075.543948	-1075.543973
14	-1075.550544	-1075.550837	-1075.542918	-1075.543231
15	-1075.550654	-1075.550725	-1075.543035	-1075.543107
16	-1075.551802	-1075.549584	-1075.542954	-1075.542579
18	-1075.550310	-1075.550310	-1075.542578	-1075.542592
19	-1075.550298	-1075.550378	-1075.542702	-1075.542756
20	-1075.550258	-1075.550337	-1075.542530	-1075.542615
21	-1075.550492	-1075.550492	-1075.542420	-1075.542420
28	-1075.549257	-1075.549285	-1075.541448	-1075.541459
58	-1075.547115	-1075.547115	-1075.539184	-1075.539184
59	-1075.547064	-1075.547101	-1075.539242	-1075.539264
61	-1075.547197	-1075.547197	-1075.539334	-1075.539334
65	-1075.545985	-1075.545985	-1075.537302	-1075.537302
66	-1075.545845	-1075.545837	-1075.537501	-1075.537476
67	-1075.545714	-1075.545689	-1075.538186	-1075.538157
68	-1075.545626	-1075.545706	-1075.538104	-1075.538158
81	-1075.544834	-1075.544753	-1075.537078	-1075.537000
82	-1075.544801	-1075.544762	-1075.537041	-1075.537014
88	-1075.544224	-1075.544216	-1075.535996	-1075.536005
90	-1075.544236	-1075.544401	-1075.536749	-1075.536900
91	-1075.544361	-1075.544273	-1075.536710	-1075.536639
92	-1075.544330	-1075.544322	-1075.536695	-1075.536673
93	-1075.544024	-1075.544024	-1075.535505	-1075.535505
103	-1075.543176	-1075.543176	-1075.535589	-1075.535589
104	-1075.543143	-1075.543143	-1075.535559	-1075.535559
105	-1075.542942	-1075.542942	-1075.535138	-1075.535138
106	-1075.542937	-1075.542937	-1075.535130	-1075.535130

Table S8. TAO-B3LYP-D3M(BJ)/aug-cc-pVTZ ground state (S_0) and lowest triplet state (T_1) energies in E_h calculated at the classical turning points of select TAO-B3LYP-D3M(BJ)/6-311G(d,p) [8]cyclacene vibrational normal mode oscillators in their vibrational ground states.

	Oscillator State v_0 Electronic State S_0		Oscillator State v_0 Electronic State T_1	
Mode	+CTP	-CTP	+CTP	-CTP
7	-1229.297569	-1229.285084	-1229.297569	-1229.285084
8	-1229.297561	-1229.285076	-1229.297561	-1229.285076
12	-1229.295959	-1229.283541	-1229.297306	-1229.284969
16	-1229.297156	-1229.284703	-1229.297156	-1229.284703
56	-1229.295587	-1229.283119	-1229.295157	-1229.282687
57	-1229.295490	-1229.283043	-1229.295501	-1229.283053
58	-1229.295350	-1229.282904	-1229.295366	-1229.282920
71	-1229.294618	-1229.282169	-1229.294614	-1229.282165
72	-1229.294635	-1229.282185	-1229.294637	-1229.282186
75	-1229.294537	-1229.281865	-1229.294537	-1229.281865
78	-1229.294806	-1229.282424	-1229.293999	-1229.281622
80	-1229.294212	-1229.281754	-1229.294212	-1229.281754
98	-1229.294978	-1229.282497	-1229.294983	-1229.282502
100	-1229.294970	-1229.282630	-1229.294972	-1229.282632
101	-1229.294984	-1229.282641	-1229.294984	-1229.282641
102	-1229.294312	-1229.281966	-1229.294637	-1229.282287
104	-1229.295358	-1229.282966	-1229.295358	-1229.282966
109	-1229.294573	-1229.282126	-1229.293309	-1229.280844
117	-1229.294366	-1229.282188	-1229.294366	-1229.282188
118	-1229.293698	-1229.281520	-1229.293698	-1229.281520
119	-1229.293957	-1229.281490	-1229.293957	-1229.281490
120	-1229.293598	-1229.281131	-1229.293598	-1229.281131
121	-1229.293537	-1229.281061	-1229.293537	-1229.281061
122	-1229.294517	-1229.282047	-1229.294517	-1229.282047

Table S9. TAO-B3LYP-D3M(BJ)/aug-cc-pVTZ ground state (S_0) and lowest triplet state (T_1) energies in E_h calculated at the classical turning points of select TAO-B3LYP-D3M(BJ)/6-311G(d,p) [8]cyclacene vibrational normal mode oscillators in their first vibrationally excited states.

	Oscillator State v_1 Electronic State S_0		Oscillator State v_1 Electronic State T_1	
Mode	+CTP	-CTP	+CTP	-CTP
7	-1229.296314	-1229.296314	-1229.283881	-1229.283881
8	-1229.296315	-1229.296315	-1229.283881	-1229.283881
12	-1229.294876	-1229.295473	-1229.282561	-1229.283157
16	-1229.295355	-1229.295355	-1229.282985	-1229.282985
56	-1229.291606	-1229.291051	-1229.279191	-1229.278633
57	-1229.291336	-1229.291343	-1229.279009	-1229.279017
58	-1229.291301	-1229.291293	-1229.278975	-1229.278967
71	-1229.290538	-1229.290538	-1229.278098	-1229.278099
72	-1229.290544	-1229.290542	-1229.278104	-1229.278103
75	-1229.288878	-1229.288878	-1229.275962	-1229.275962
78	-1229.289121	-1229.288691	-1229.276655	-1229.276219
80	-1229.288737	-1229.288737	-1229.276392	-1229.276392
98	-1229.288640	-1229.288642	-1229.276230	-1229.276233
100	-1229.288772	-1229.288774	-1229.276703	-1229.276705
101	-1229.288789	-1229.288789	-1229.276706	-1229.276706
102	-1229.289079	-1229.287943	-1229.276743	-1229.275599
104	-1229.289066	-1229.289066	-1229.276799	-1229.276799
109	-1229.288078	-1229.286709	-1229.275620	-1229.274222
117	-1229.287205	-1229.287205	-1229.275433	-1229.275433
118	-1229.287073	-1229.287073	-1229.275301	-1229.275301
119	-1229.286427	-1229.286427	-1229.274018	-1229.274018
120	-1229.287657	-1229.287657	-1229.275248	-1229.275248
121	-1229.287233	-1229.287233	-1229.274900	-1229.274900
122	-1229.287324	-1229.287324	-1229.275001	-1229.275001

Table S10. TAO-B3LYP-D3M(BJ)/aug-cc-pVTZ ground state (S_0) and lowest triplet state (T_1) energies in E_h calculated at the classical turning points of select TAO-B3LYP-D3M(BJ)/6-311G(d,p) [9]cyclacene vibrational normal mode oscillators in their vibrational ground states.

	Oscillator State v_0 Electronic State S_0		Oscillator State v_0 Electronic State T_1	
Mode	+CTP	-CTP	+CTP	-CTP
14	-1383.006553	-1383.006553	-1383.001876	-1383.001876
15	-1383.006552	-1383.006552	-1383.001876	-1383.001876
19	-1383.006290	-1383.006290	-1383.001631	-1383.001641
22	-1383.006300	-1383.006300	-1383.001627	-1383.001627
24	-1383.006252	-1383.006244	-1383.001582	-1383.001571
26	-1383.006197	-1383.006184	-1383.001544	-1383.001528
27	-1383.006175	-1383.006187	-1383.001524	-1383.001530
28	-1383.006175	-1383.006162	-1383.001530	-1383.001516
29	-1383.006243	-1383.006243	-1383.001533	-1383.001533
30	-1383.006166	-1383.006154	-1383.001487	-1383.001475
31	-1383.006149	-1383.006172	-1383.001450	-1383.001502
32	-1383.006202	-1383.006053	-1383.001576	-1383.001336
61	-1383.005269	-1383.005275	-1383.000584	-1383.000591
62	-1383.005282	-1383.005261	-1383.000599	-1383.000577
67	-1383.005245	-1383.005245	-1383.000576	-1383.000576
68	-1383.005251	-1383.005251	-1383.000576	-1383.000576
81	-1383.005180	-1383.005180	-1383.000505	-1383.000505
85	-1383.004709	-1383.004709	-1382.999868	-1382.999868
86	-1383.004671	-1383.004652	-1382.999954	-1382.999932
87	-1383.004660	-1383.004656	-1382.999942	-1382.999936
88	-1383.004630	-1383.004631	-1382.999892	-1382.999891
107	-1383.004290	-1383.004286	-1382.999631	-1382.999633
108	-1383.004286	-1383.004287	-1382.999632	-1382.999633
116	-1383.004107	-1383.004110	-1382.999368	-1382.999371
117	-1383.004137	-1383.004137	-1382.999492	-1382.999492
118	-1383.004135	-1383.004136	-1382.999493	-1382.999486
119	-1383.004140	-1383.004137	-1382.999491	-1382.999489
120	-1383.004142	-1383.004130	-1382.999500	-1382.999480
133	-1383.003824	-1383.003824	-1382.999212	-1382.999212
134	-1383.003817	-1383.003817	-1382.999215	-1382.999215
137	-1383.003667	-1383.003667	-1382.999029	-1382.999029
138	-1383.003668	-1383.003668	-1382.999028	-1382.999028

Table S11. TAO-B3LYP-D3M(BJ)/aug-cc-pVTZ ground state (S_0) and lowest triplet state (T_1) energies in E_h calculated at the classical turning points of select TAO-B3LYP-D3M(BJ)/6-311G(d,p) [9]cyclacene vibrational normal mode oscillators in their first vibrationally excited states.

	Oscillator State v_0 Electronic State S_0		Oscillator State v_0 Electronic State T_1	
Mode	+CTP	-CTP	+CTP	-CTP
14	-1383.005165	-1383.005165	-1383.000492	-1383.000492
15	-1383.005164	-1383.005164	-1383.000491	-1383.000491
19	-1383.004376	-1383.004383	-1382.999766	-1382.999780
22	-1383.004410	-1383.004410	-1382.999743	-1382.999743
24	-1383.004264	-1383.004251	-1382.999599	-1382.999584
26	-1383.004085	-1383.004043	-1382.999477	-1382.999436
27	-1383.004001	-1383.004023	-1382.999417	-1382.999430
28	-1383.004019	-1383.003996	-1382.999430	-1382.999407
29	-1383.004246	-1383.004246	-1382.999468	-1382.999468
30	-1383.004000	-1383.003983	-1382.999312	-1382.999293
31	-1383.003959	-1383.004009	-1382.999245	-1382.999337
32	-1383.004006	-1383.003755	-1382.999418	-1382.999006
61	-1383.001192	-1383.001205	-1382.996502	-1382.996516
62	-1383.001218	-1383.001181	-1382.996529	-1382.996491
67	-1383.001239	-1383.001239	-1382.996583	-1382.996583
68	-1383.001239	-1383.001239	-1382.996577	-1382.996577
81	-1383.001052	-1383.001052	-1382.996384	-1382.996384
85	-1382.999636	-1382.999636	-1382.994462	-1382.994462
86	-1382.999506	-1382.999472	-1382.994717	-1382.994666
87	-1382.999479	-1382.999474	-1382.994694	-1382.994692
88	-1382.999372	-1382.999373	-1382.994536	-1382.994535
107	-1382.998369	-1382.998369	-1382.993750	-1382.993746
108	-1382.998364	-1382.998367	-1382.993745	-1382.993748
116	-1382.997834	-1382.997840	-1382.992960	-1382.992965
117	-1382.997898	-1382.997907	-1382.993324	-1382.993326
118	-1382.997910	-1382.997896	-1382.993331	-1382.993307
119	-1382.997912	-1382.997900	-1382.993327	-1382.993316
120	-1382.997936	-1382.997859	-1382.993364	-1382.993280
133	-1382.996956	-1382.996956	-1382.992551	-1382.992551
134	-1382.996959	-1382.996959	-1382.992556	-1382.992556
137	-1382.996508	-1382.996508	-1382.991949	-1382.991949
138	-1382.996505	-1382.996505	-1382.991943	-1382.991943

4. Infrared Spectroscopy Simulations

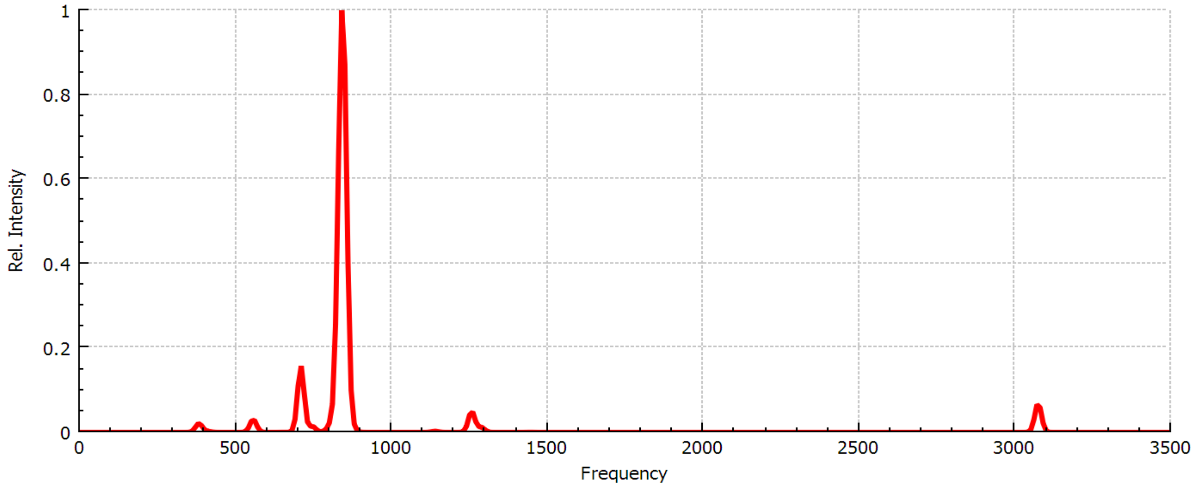

Figure S2. The harmonic TAO-DFT IR spectrum of [6]cyclacene. The X-axis is in units of cm^{-1}.

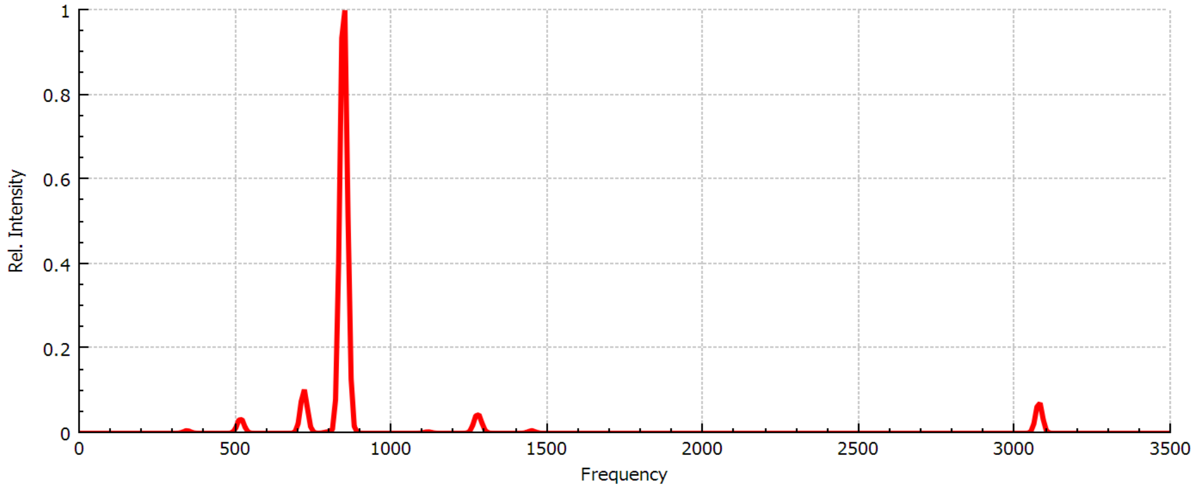

Figure S3. The harmonic TAO-DFT IR spectrum of [7]cyclacene. The X-axis is in units of cm^{-1}.

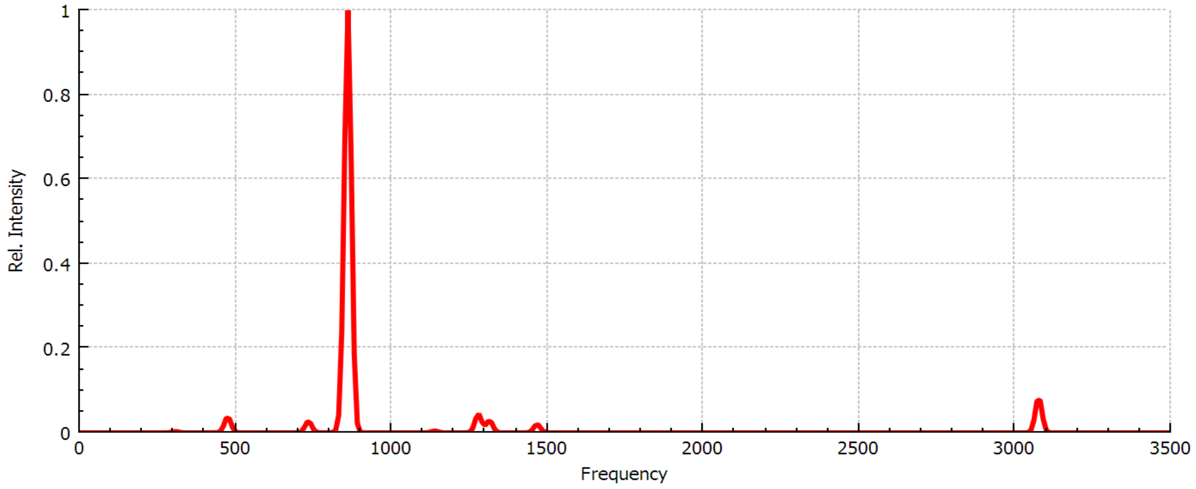

Figure S4. The harmonic TAO-DFT IR spectrum of [8]cyclacene. The X-axis is in units of cm^{-1}.

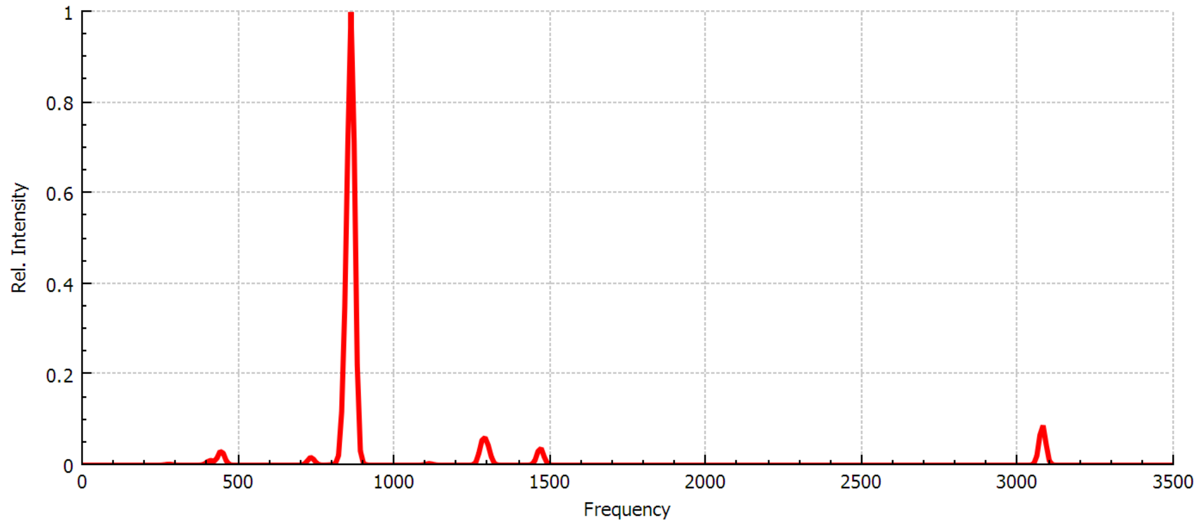

Figure S5. The harmonic TAO-DFT IR spectrum of [9]cyclacene. The X-axis is in units of cm^{-1}.

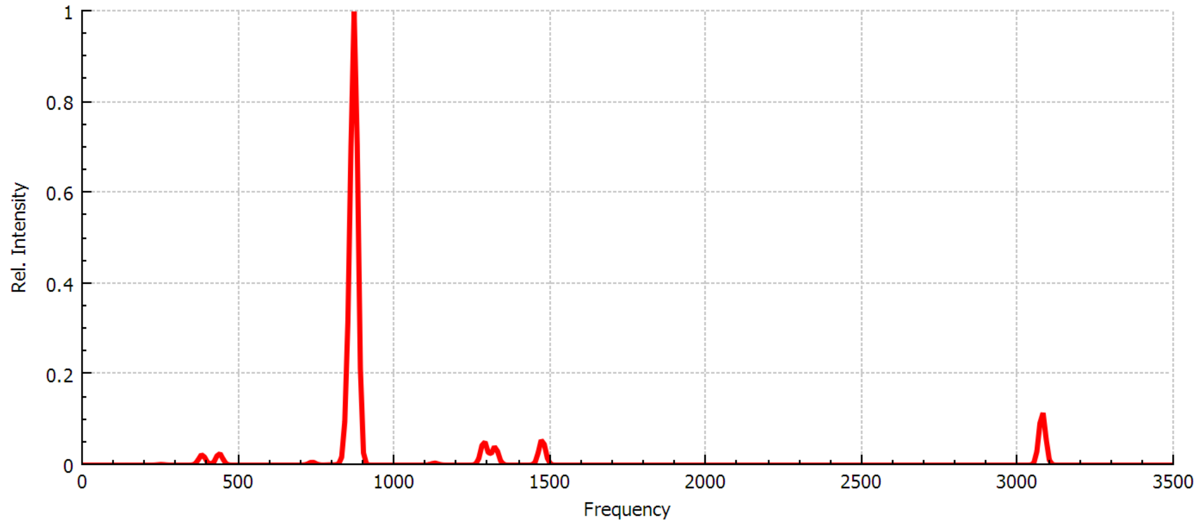

Figure S6. The harmonic TAO-DFT IR spectrum of [10]cyclacene. The X-axis is in units of cm^{-1}.

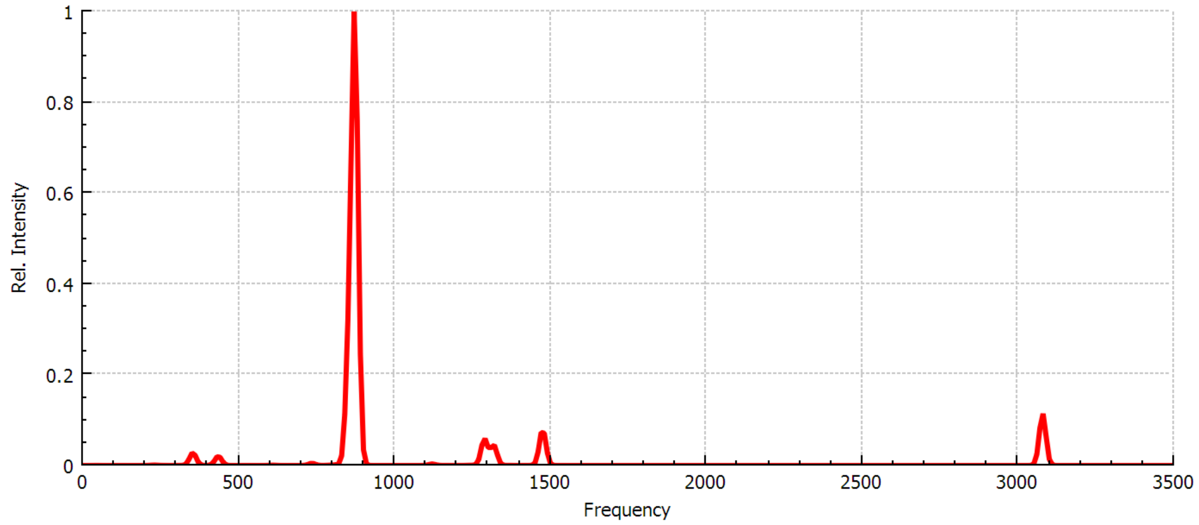

Figure S7. The harmonic TAO-DFT IR spectrum of [11]cyclacene. The X-axis is in units of cm^{-1}.

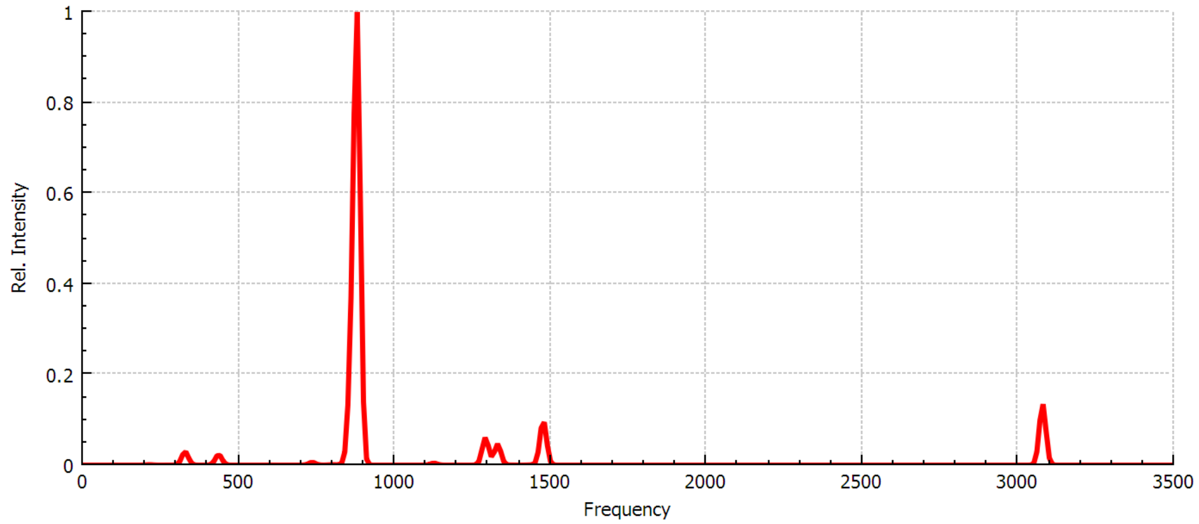

Figure S8. The harmonic TAO-DFT IR spectrum of [12]cyclacene. The X-axis is in units of cm^{-1}.

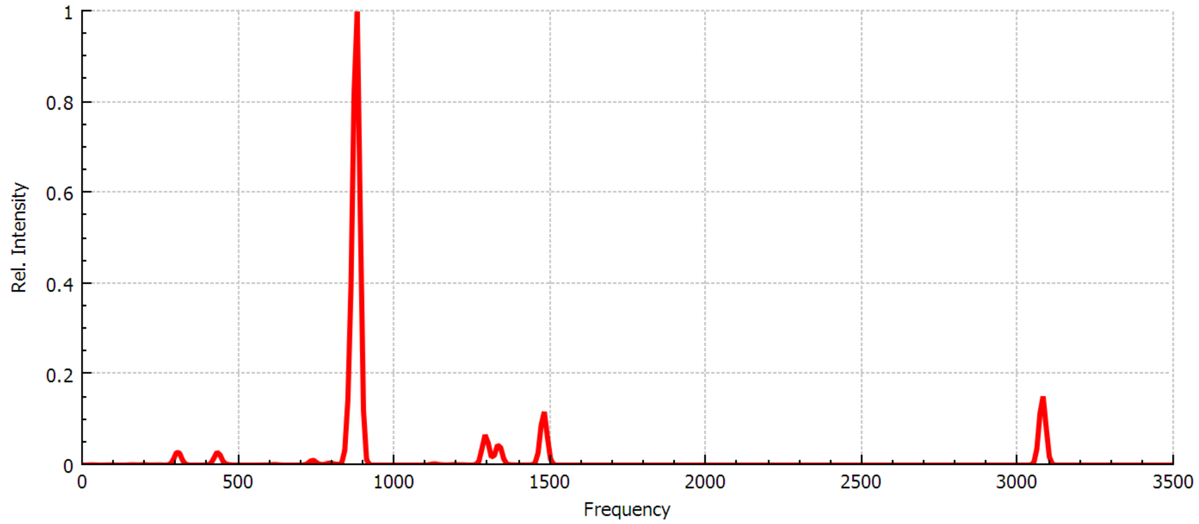

Figure S9. The harmonic TAO-DFT IR spectrum of [13]cyclacene. The X-axis is in units of cm^{-1}.

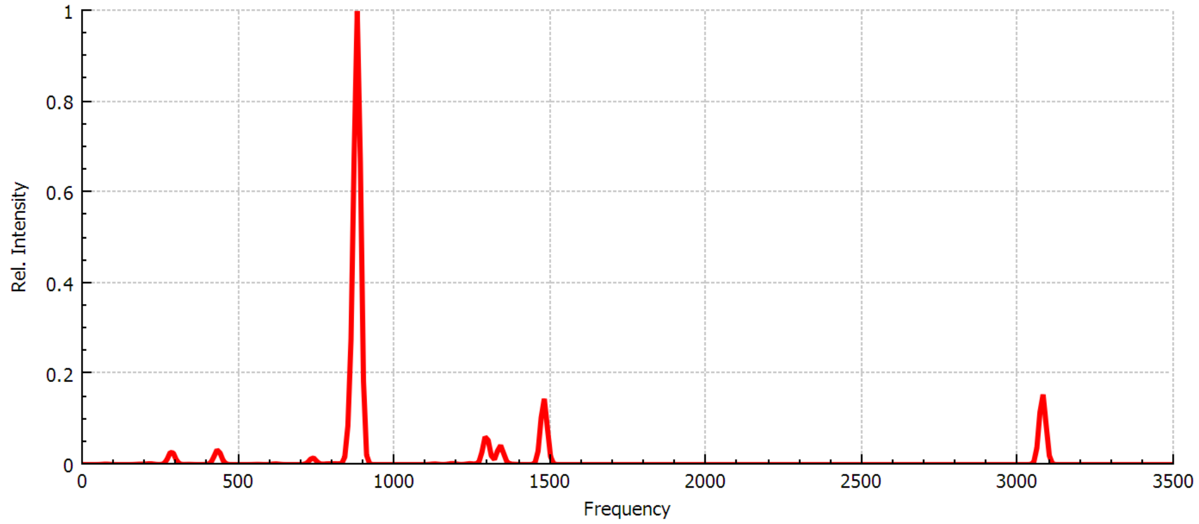

Figure S10. The harmonic TAO-DFT IR spectrum of [14]cyclacene. The X-axis is in units of cm^{-1}.

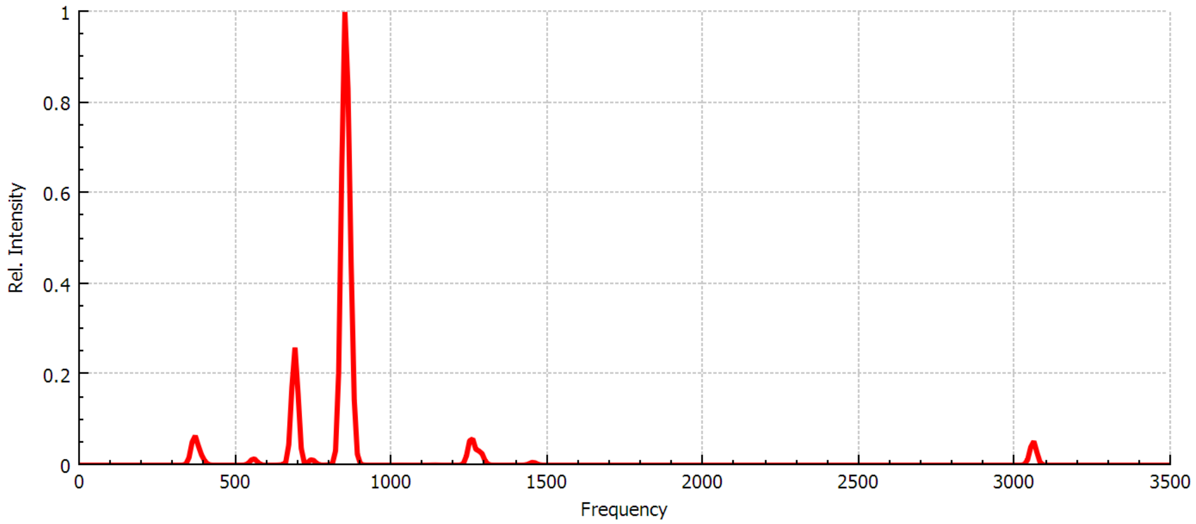

Figure S11. The harmonic KS-DFT IR spectrum of [6]cyclacene. The X-axis is in units of cm^{-1}.

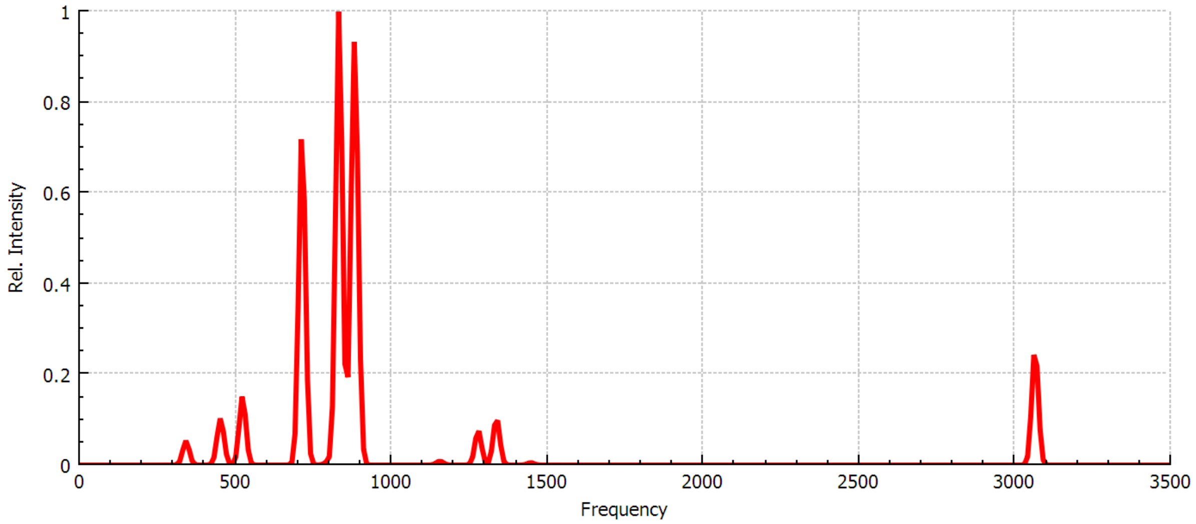

Figure S12. The harmonic KS-DFT IR spectrum of [7]cyclacene. The X-axis is in units of cm^{-1}.

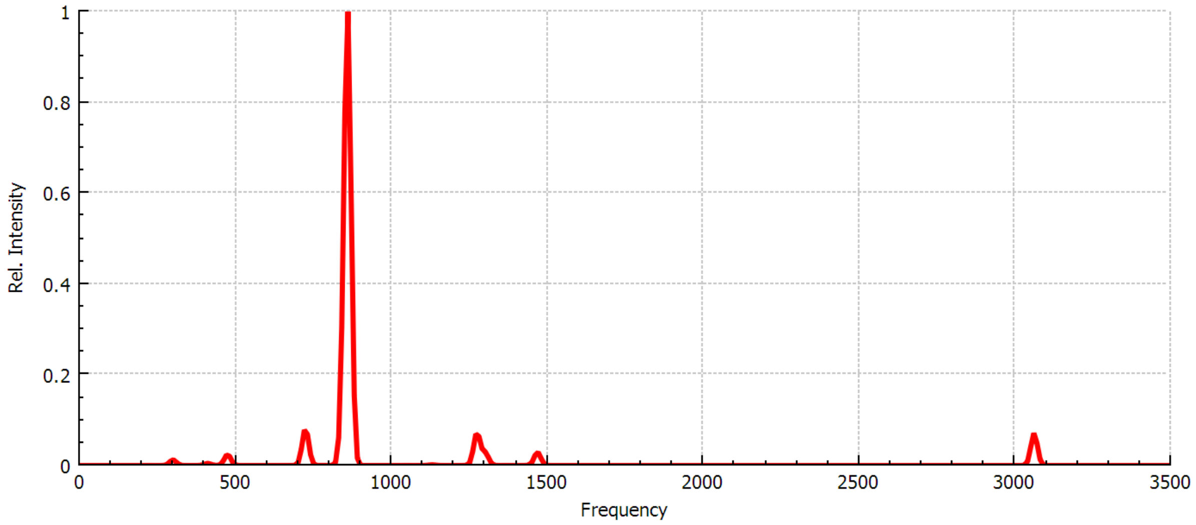

Figure S13. The harmonic KS-DFT IR spectrum of [8]cyclacene. The X-axis is in units of cm^{-1}.

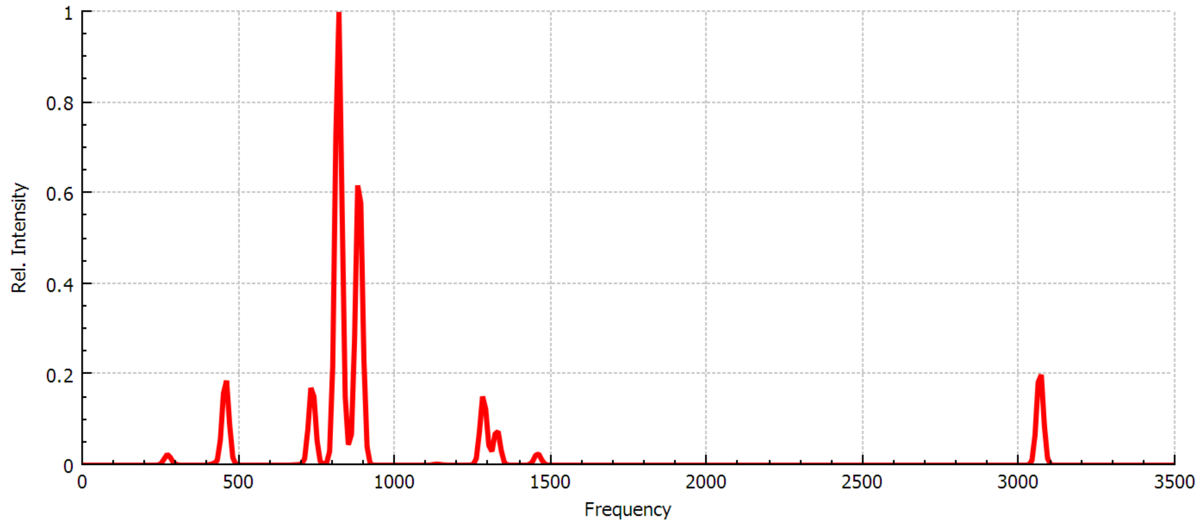

Figure S14. The harmonic KS-DFT IR spectrum of [9]cyclacene. The X-axis in cm^{-1}.

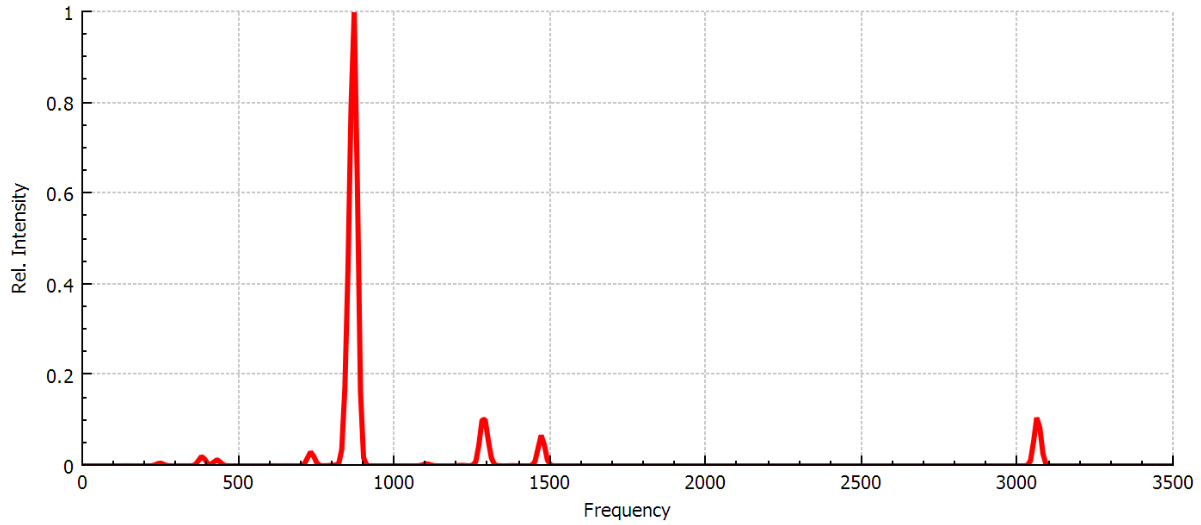

Figure S15. The harmonic KS-DFT IR spectrum of [10]cyclacene. The X-axis is in units of cm^{-1}.

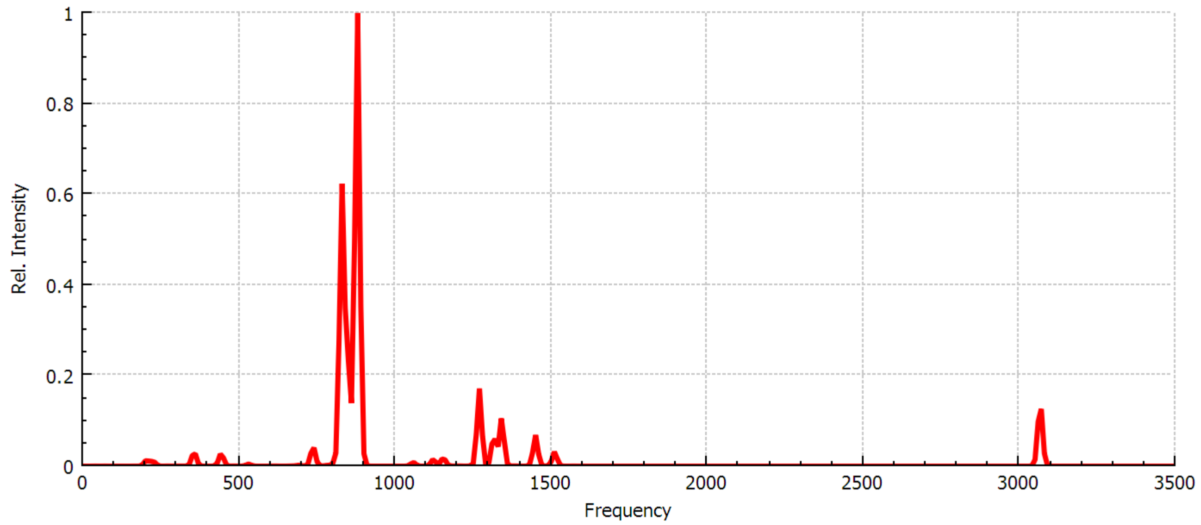

Figure S16. The harmonic KS-DFT IR spectrum of [11]cyclacene. The X-axis is in units of cm^{-1}.

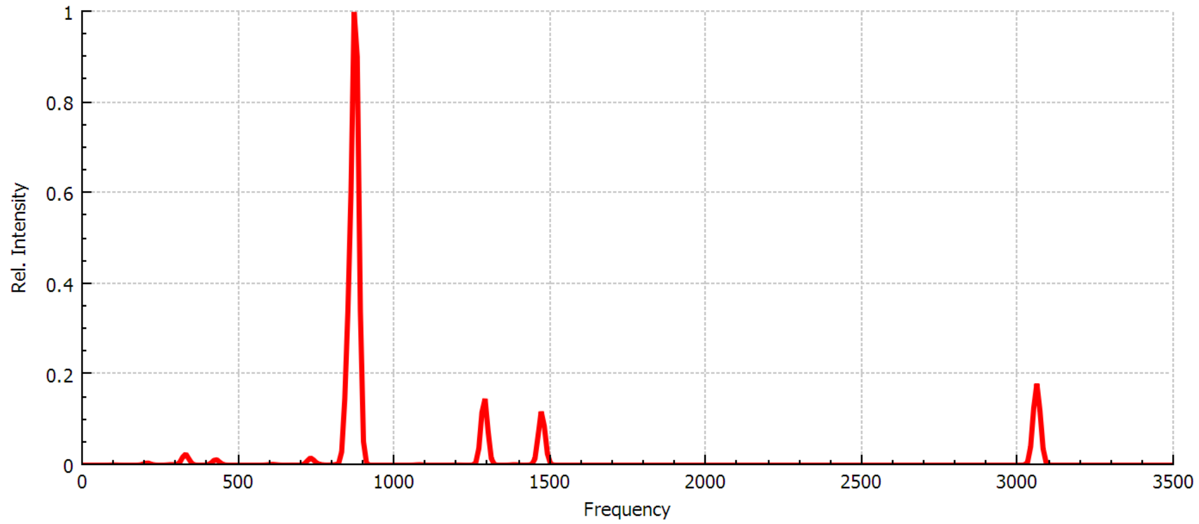

Figure S17. The harmonic KS-DFT IR spectrum of [12]cyclacene. The X-axis is in units of cm^{-1}.

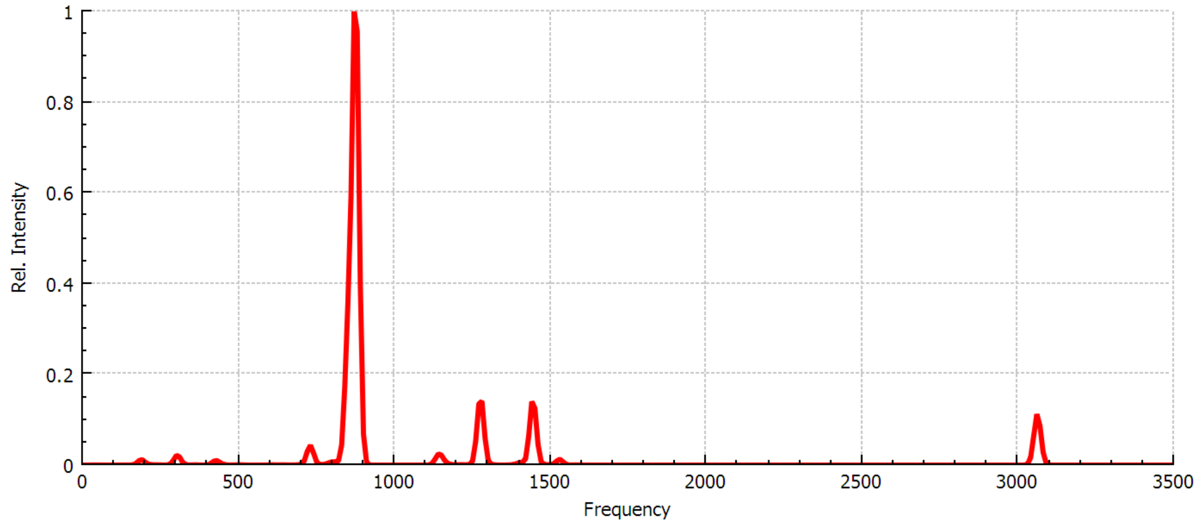

Figure S18. The harmonic KS-DFT IR spectrum of [13]cyclacene. The X-axis is in units of cm^{-1}.

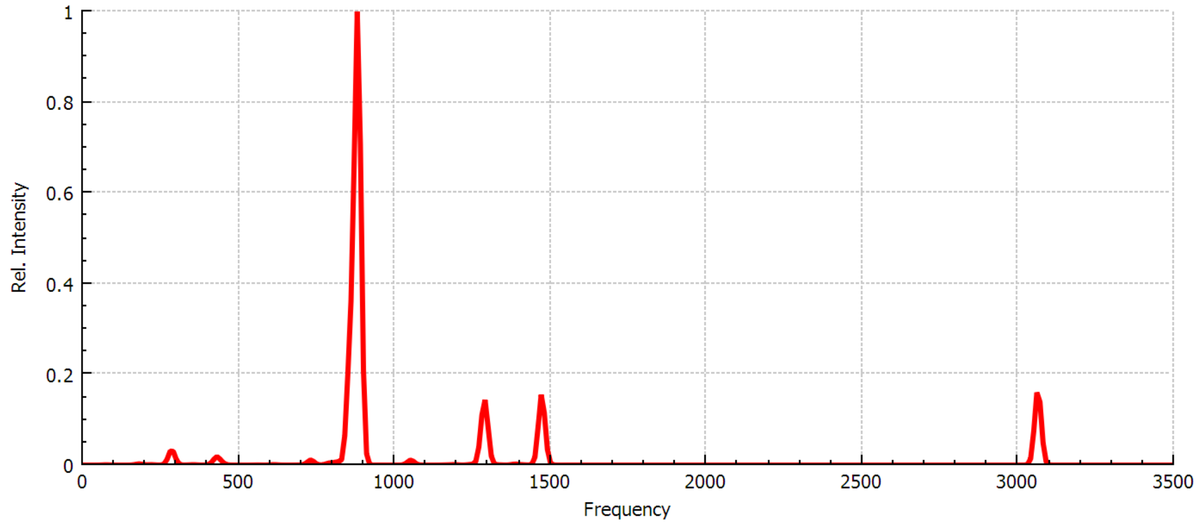

Figure S19. The harmonic KS-DFT IR spectrum of [14]cyclacene. The X-axis is in units of cm^{-1}.

5. Raman Spectroscopy Simulations

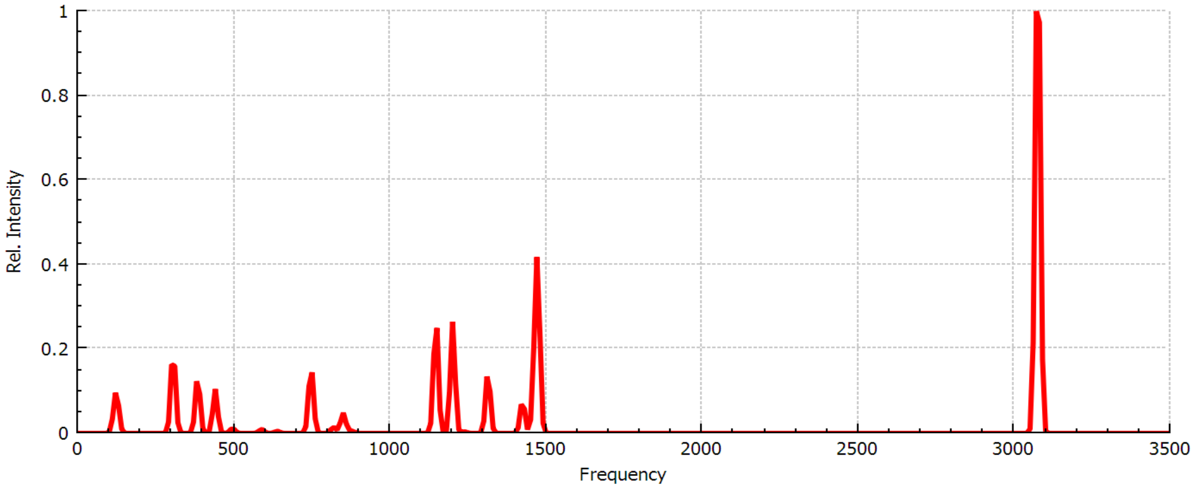

Figure S20. The harmonic TAO-DFT Raman spectrum of [6]cyclacene. The X-axis is in units of cm^{-1}.

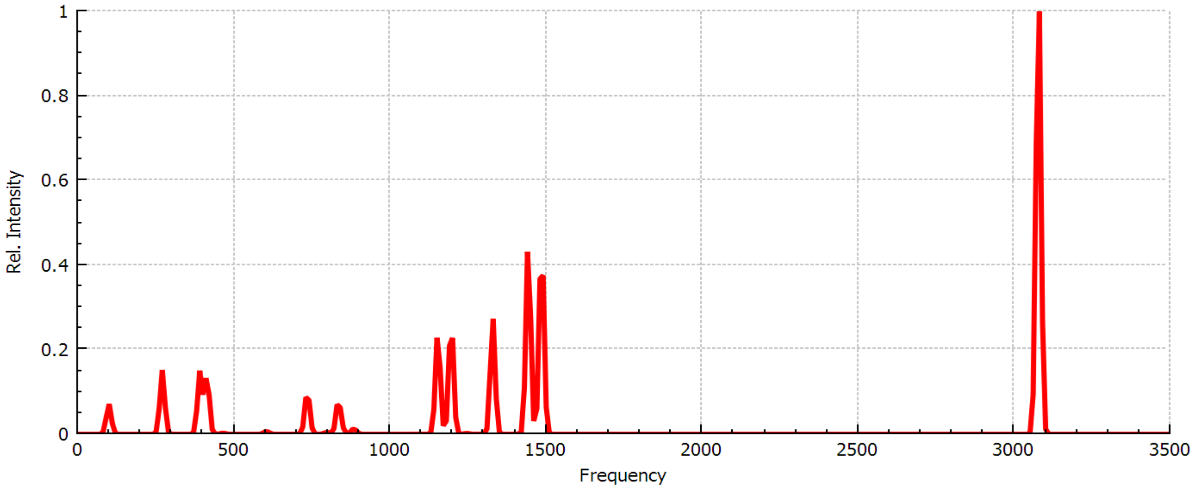

Figure S21. The harmonic TAO-DFT Raman spectrum of [7]cyclacene. The X-axis is in units of cm^{-1}.

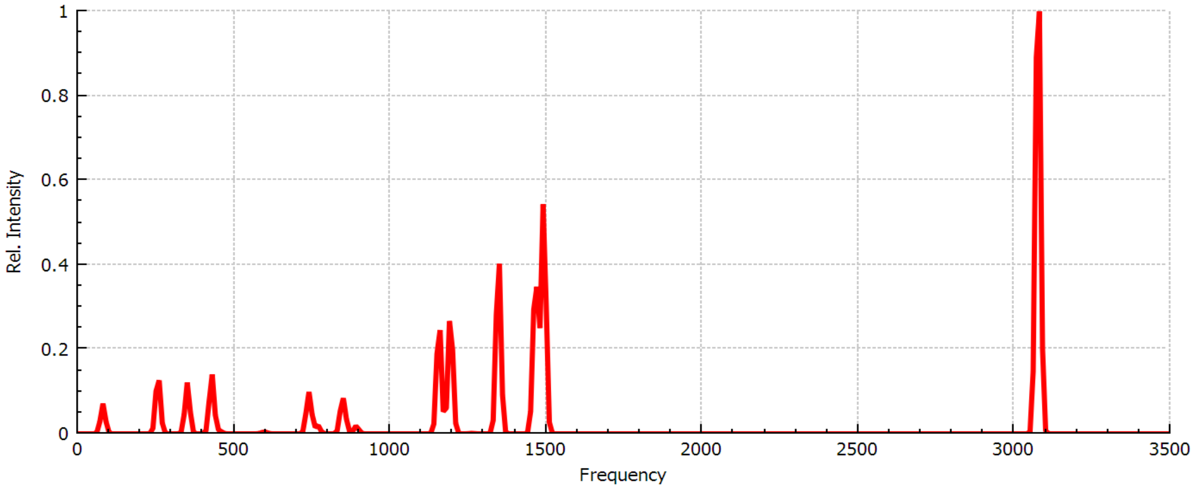

Figure S22. The harmonic TAO-DFT Raman spectrum of [8]cyclacene. The X-axis is in units of cm^{-1}.

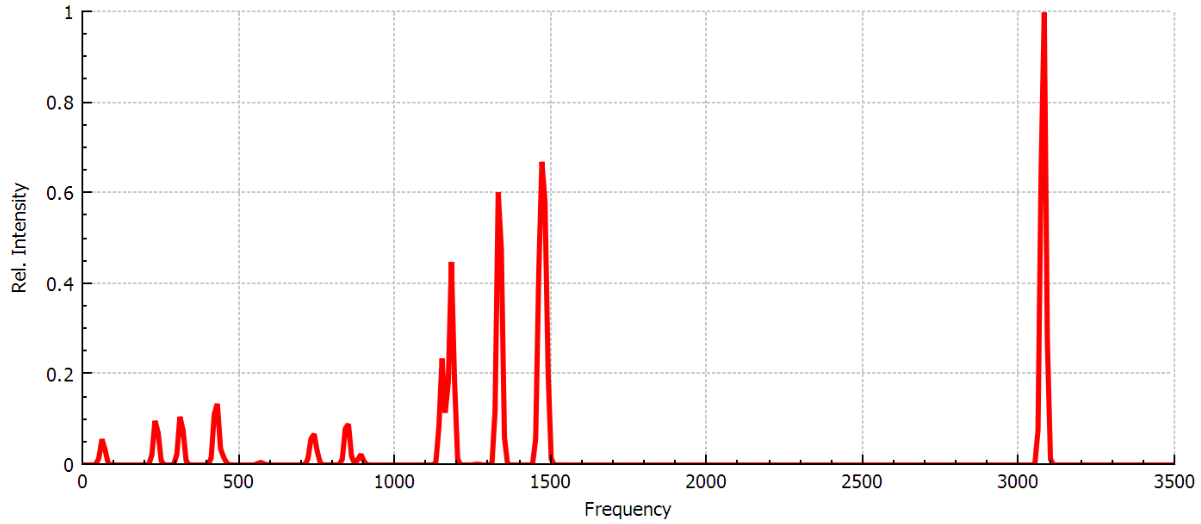

Figure S23. The harmonic TAO-DFT Raman spectrum of [9]cyclacene. The X-axis is in units of cm^{-1}.

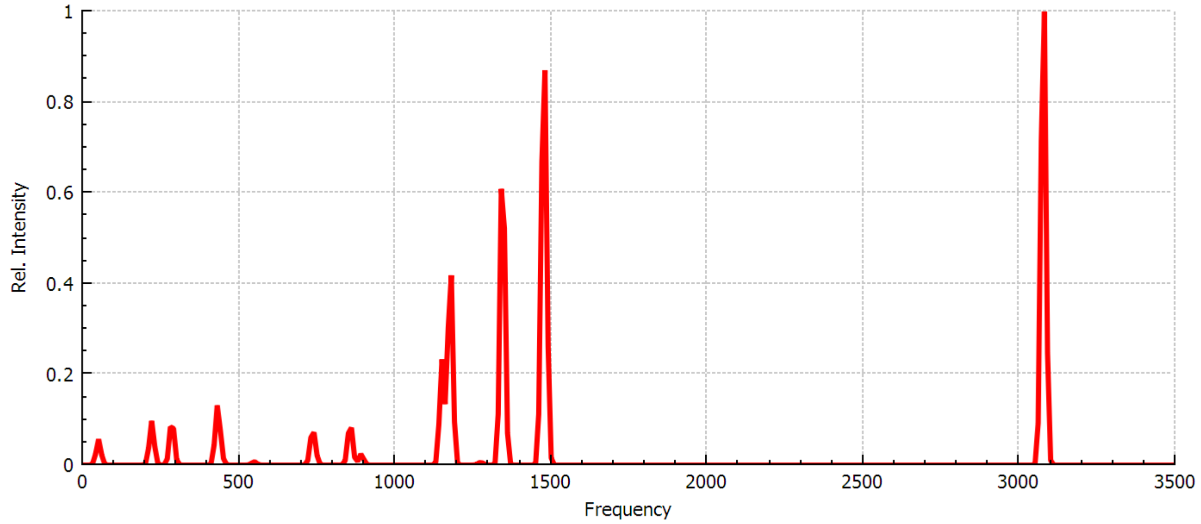

Figure S24. The harmonic TAO-DFT Raman spectrum of [10]cyclacene. The X-axis is in cm^{-1}.

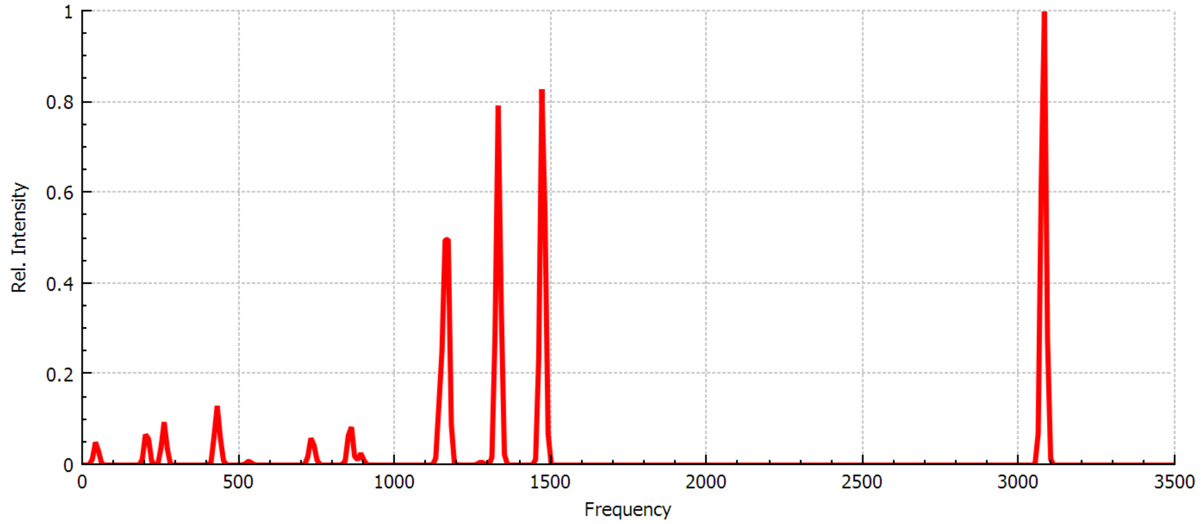

Figure S25. The harmonic TAO-DFT Raman spectrum of [11]cyclacene. The X-axis is in cm^{-1}.

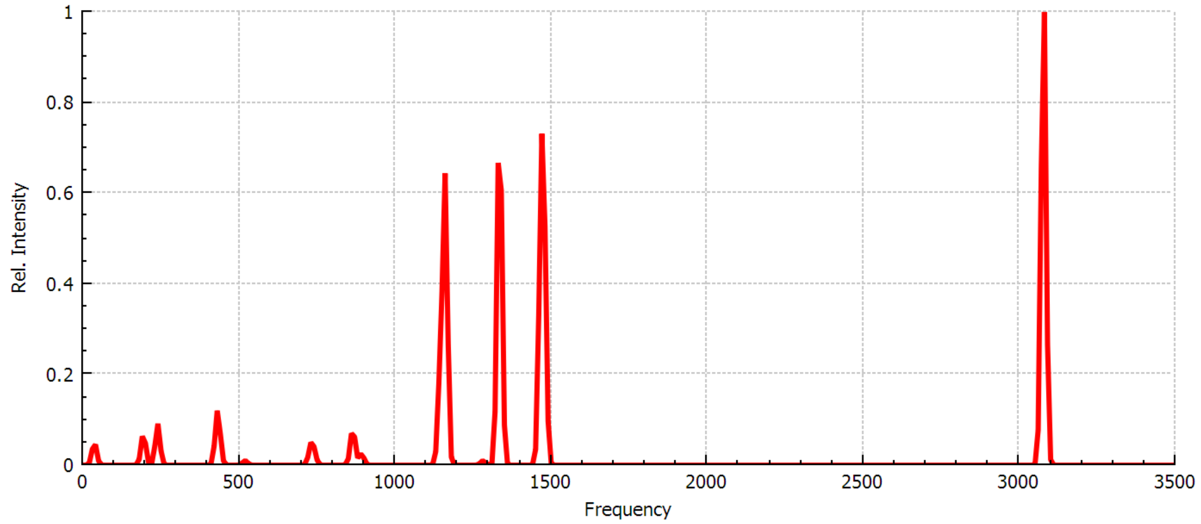

Figure S26. The harmonic TAO-DFT Raman spectrum of [12]cyclacene. The X-axis is in cm^{-1}.

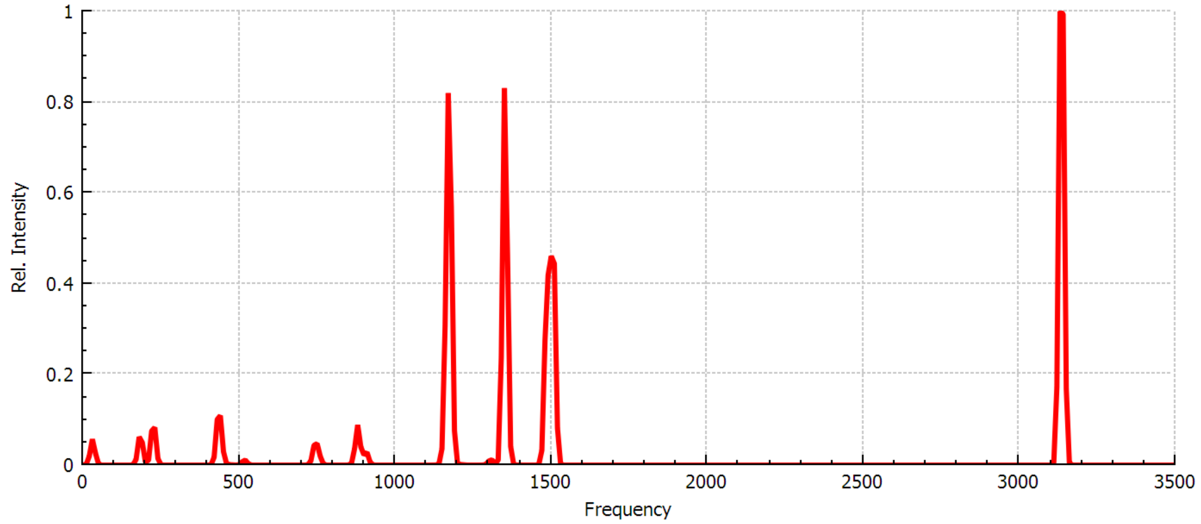

Figure S27. The harmonic TAO-DFT Raman spectrum of [13]cyclacene. The X-axis is in cm^{-1}.

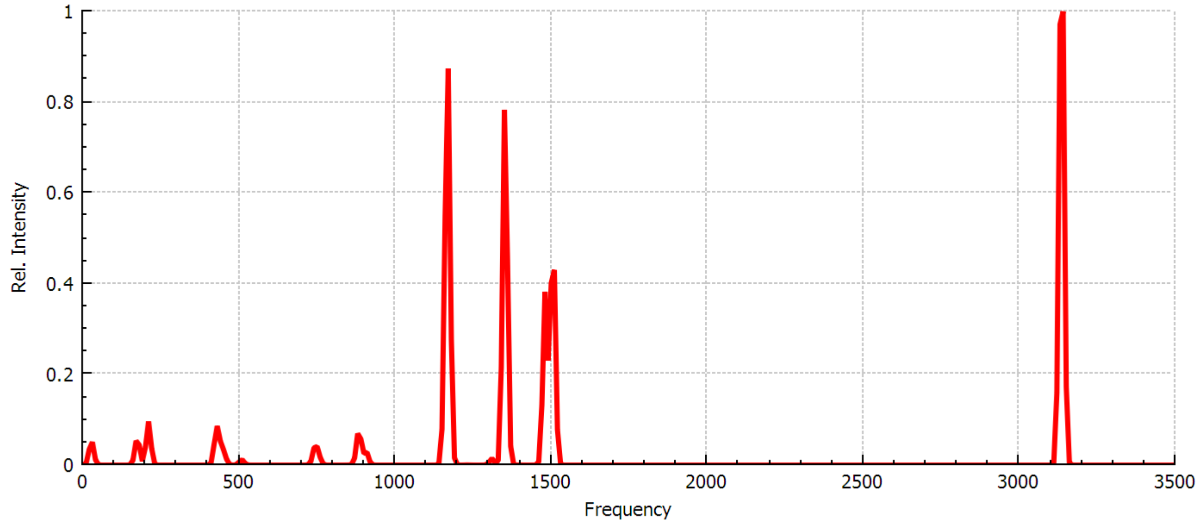

Figure S28. The harmonic TAO-DFT Raman spectrum of [14]cyclacene. The X-axis is in cm^{-1}.

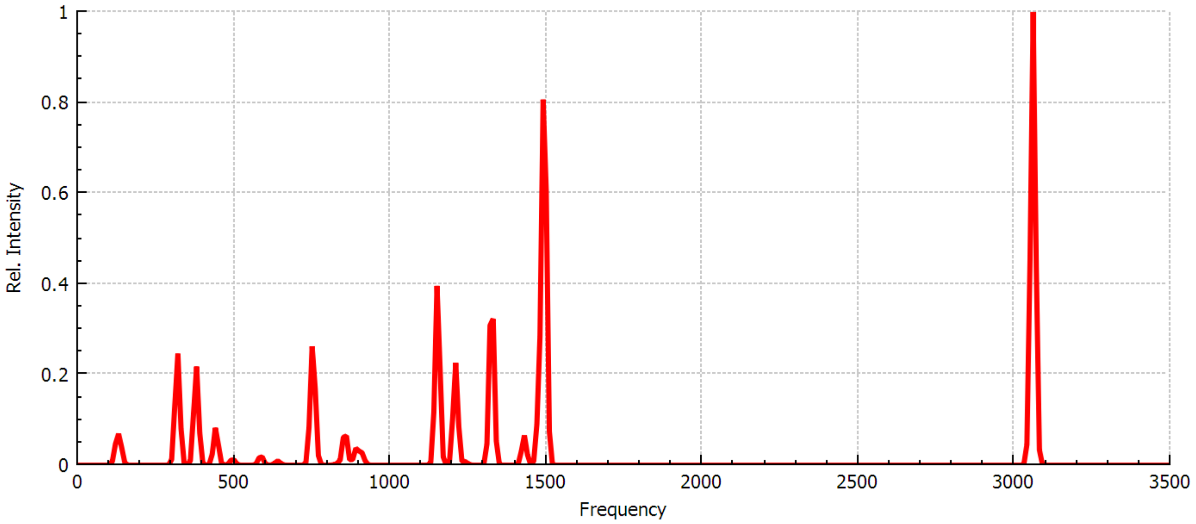

Figure S29. The harmonic KS-DFT Raman spectrum of [6]cyclacene. The X-axis is in cm^{-1}.

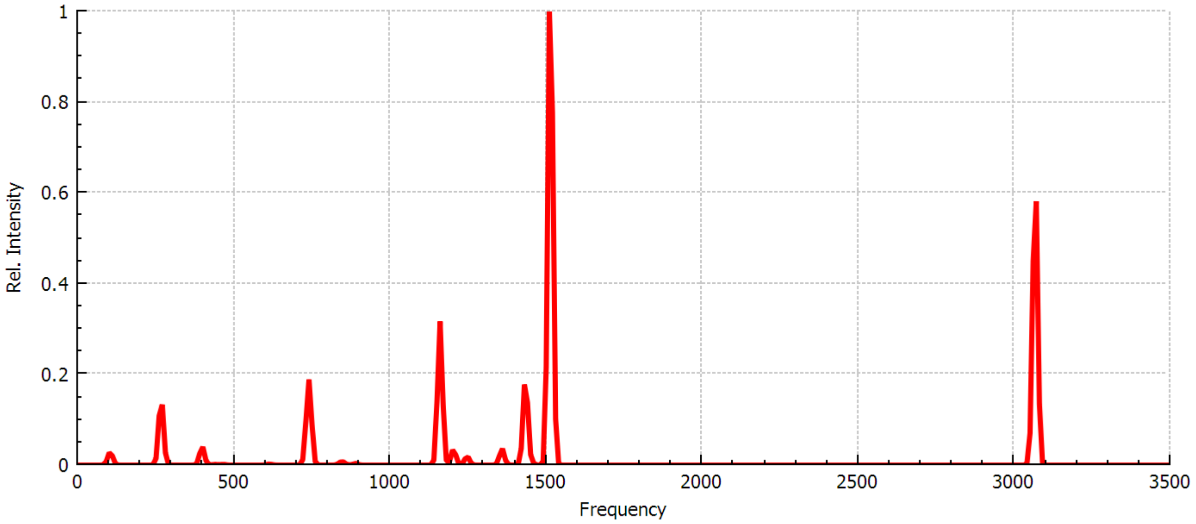

Figure S30. The harmonic KS-DFT Raman spectrum of [7]cyclacene. The X-axis is in cm^{-1}.

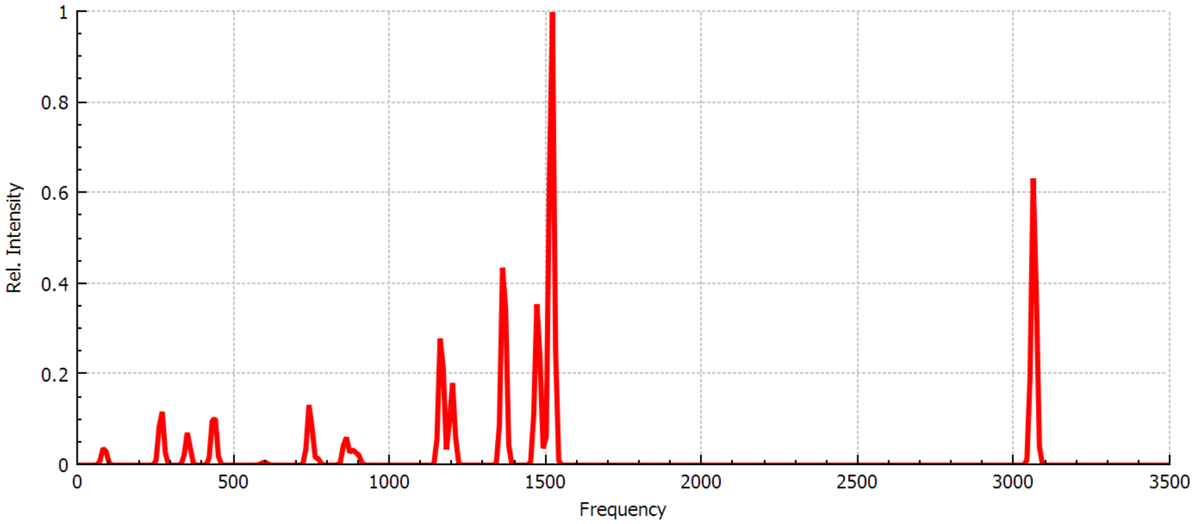

Figure S31. The harmonic KS-DFT Raman spectrum of [8]cyclacene. The X-axis is in cm^{-1}.

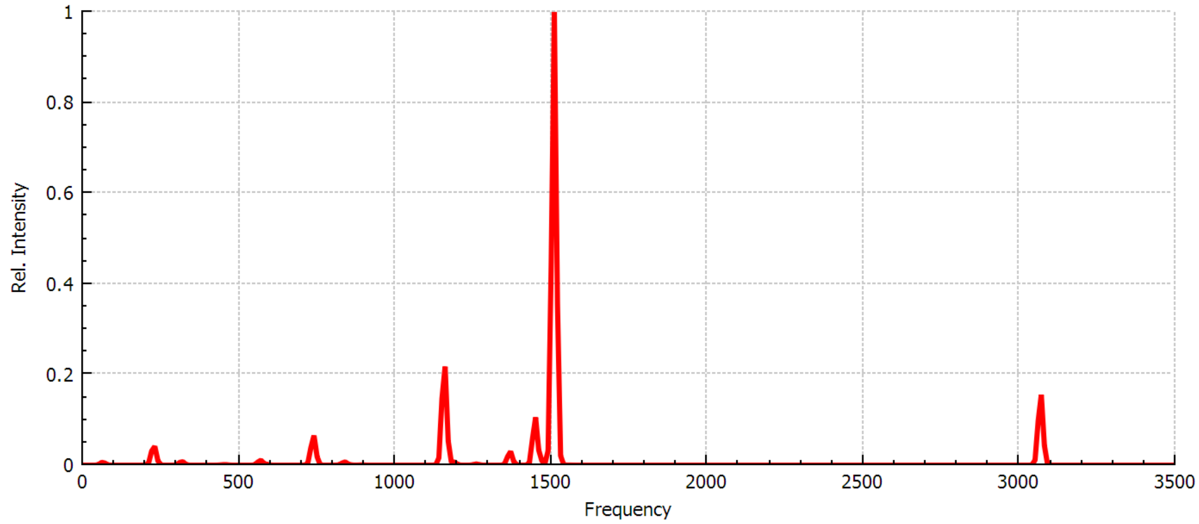

Figure S32. The harmonic KS-DFT Raman spectrum of [9]cyclacene. The X-axis is in units of cm^{-1}.

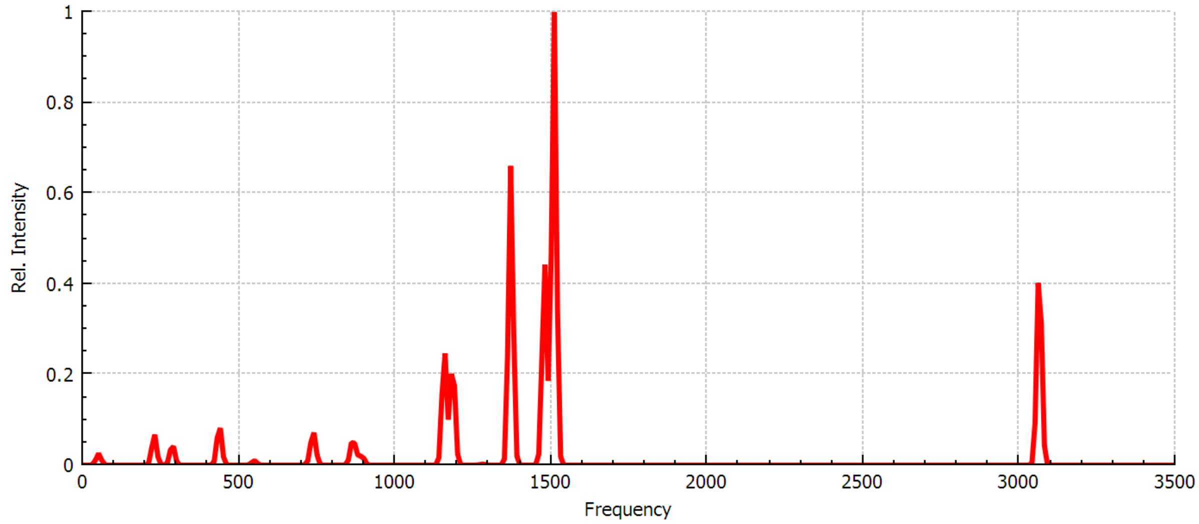

Figure S33. The harmonic KS-DFT Raman spectrum of [10]cyclacene. The X-axis is in units of cm^{-1}.

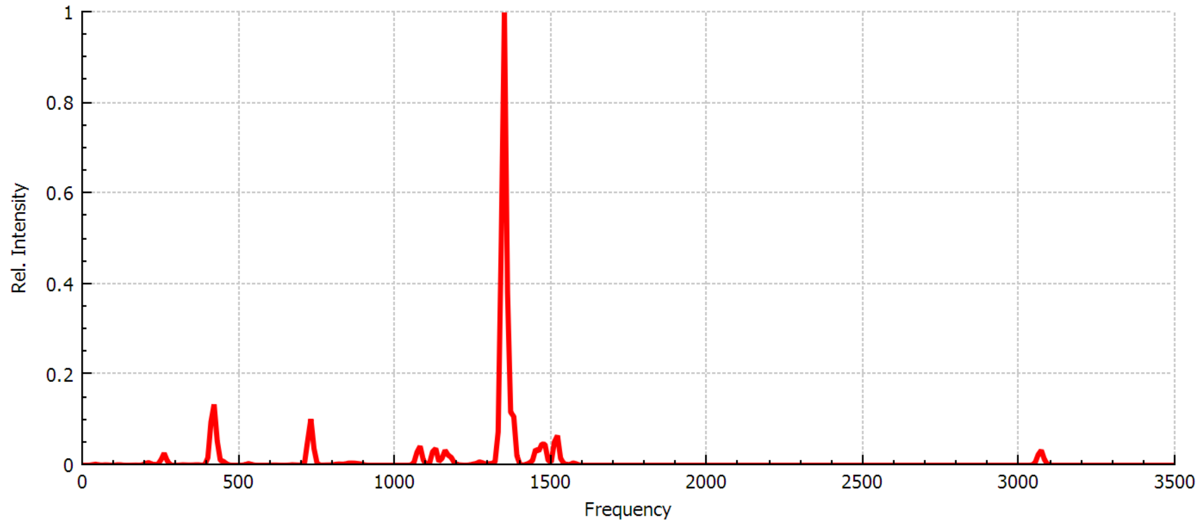

Figure S34. The harmonic KS-DFT Raman spectrum of [11]cyclacene. The X-axis is in units of cm^{-1}.

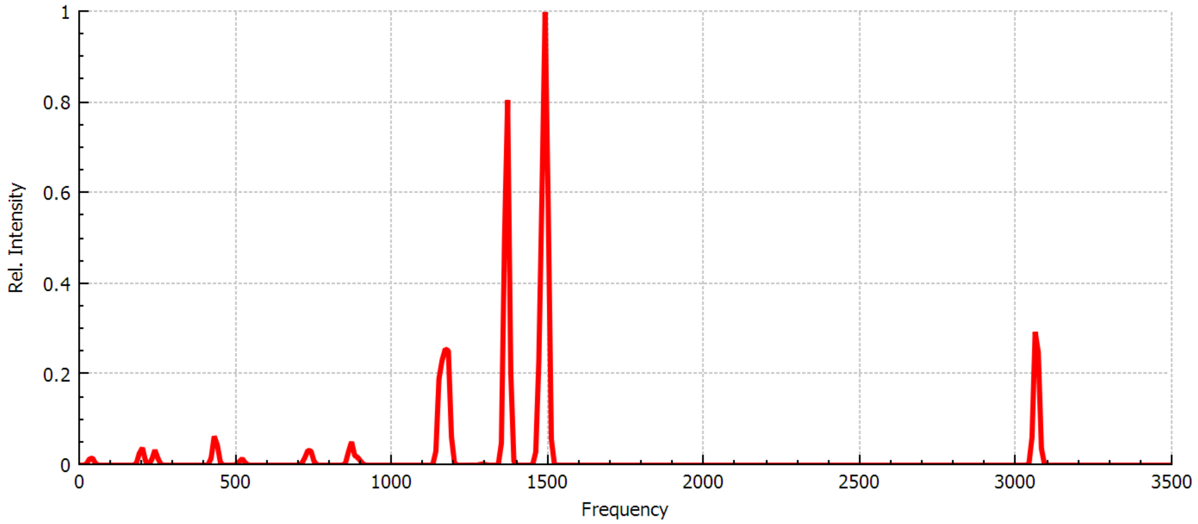

Figure S35. The harmonic KS-DFT Raman spectrum of [12]cyclacene. The X-axis is in units of cm^{-1}.

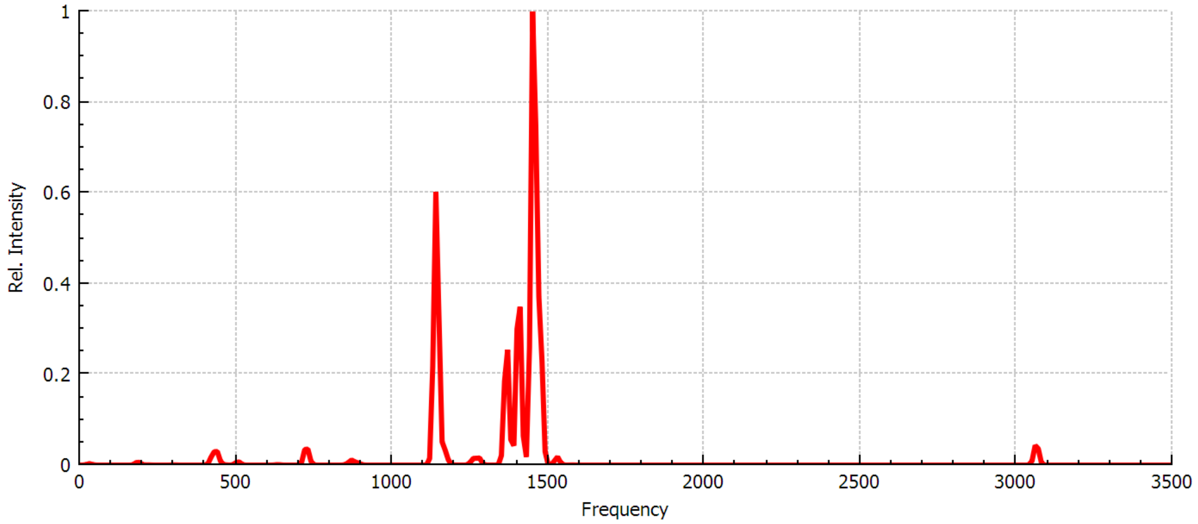

Figure S36. The harmonic KS-DFT Raman spectrum of [13]cyclacene. The X-axis is in units of cm^{-1}.

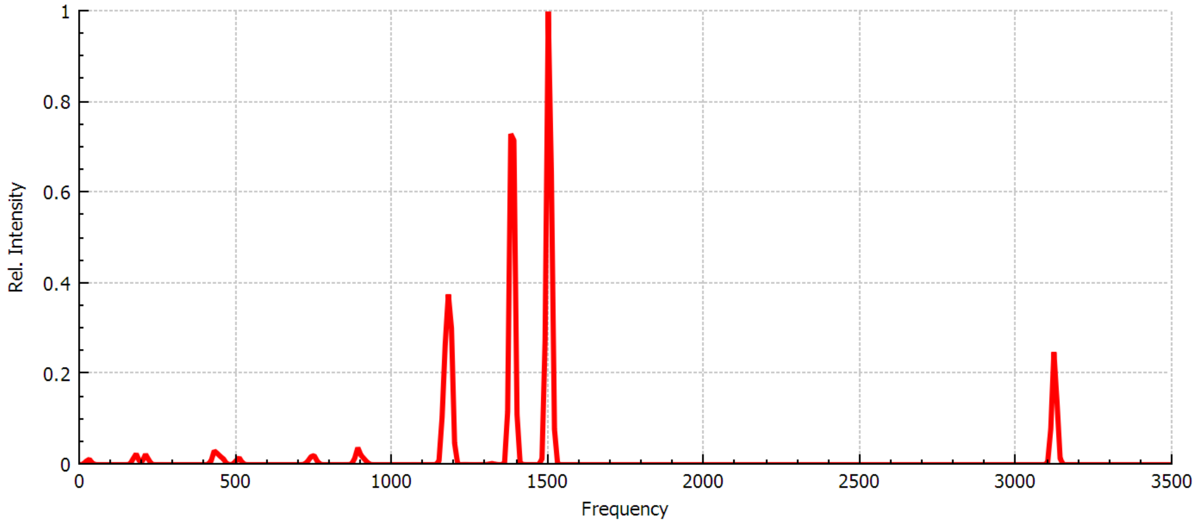

Figure S37. The harmonic KS-DFT Raman spectrum of [14]cyclacene. The X-axis is in units of cm^{-1}.

6. Nuclear Vibrational Frequencies

Table S12. Harmonic nuclear vibrational frequencies and ΔTAO shifts (in cm^{-1}) for the KS-B3LYP-D3M(BJ)/6-311G(d,p) and TAO-B3LYP-D3M(BJ)/6-311G(d,p) S_0 states of [6]cyclacene.

KS-DFT	TAO-DFT	ΔTAO
127.71	122.53	-5.18
138.99	130.07	-8.92
177.77	180.48	2.71
184.81	183.42	-1.39
243.01	256.44	13.43
325.07	314.42	-10.65
327.52	315.36	-12.16
331.03	321.43	-9.60
339.20	329.05	-10.15
379.02	394.28	15.26
389.67	395.25	5.58
392.66	396.95	4.29
397.10	419.18	22.08
403.80	419.88	16.08
425.82	423.47	-2.35
426.82	423.58	-3.24
429.37	443.32	13.95
456.16	454.13	-2.03
473.64	469.69	-3.95
511.60	511.38	-0.22
575.08	573.73	-1.35
575.82	574.76	-1.06
585.91	589.72	3.81
604.66	607.30	2.64
605.55	607.49	1.94
658.94	658.44	-0.50
662.55	659.44	-3.11
673.84	671.78	-2.06
674.64	674.98	0.34
678.48	676.55	-1.93
712.27	732.24	19.97
767.67	767.97	0.30
768.99	769.84	0.85
774.02	769.85	-4.17
779.32	770.84	-8.48
780.24	773.28	-6.96
794.19	774.90	-19.29
826.40	808.26	-18.14

828.15	813.86	-14.29
857.26	835.48	-21.78
859.60	842.91	-16.69
861.62	851.26	-10.36
865.81	860.57	-5.24
874.24	860.83	-13.41
879.01	872.67	-6.34
882.21	874.96	-7.25
884.99	877.40	-7.59
890.81	877.98	-12.83
894.11	882.75	-11.36
897.13	889.41	-7.72
918.47	893.79	-24.68
919.93	896.18	-23.75
923.63	908.34	-15.29
939.84	922.05	-17.79
1064.43	1134.87	70.44
1175.46	1169.92	-5.54
1176.20	1175.53	-0.67
1189.21	1178.36	-10.85
1190.27	1183.16	-7.11
1201.45	1185.80	-15.65
1227.28	1221.03	-6.25
1227.59	1221.26	-6.33
1247.98	1239.85	-8.13
1250.74	1240.46	-10.28
1280.52	1276.75	-3.77
1281.31	1278.73	-2.58
1287.38	1282.96	-4.42
1289.27	1287.67	-1.60
1292.28	1289.92	-2.36
1294.73	1290.54	-4.19
1297.04	1298.22	1.18
1326.26	1329.67	3.41
1326.75	1330.30	3.55
1349.16	1344.51	-4.65
1353.83	1356.57	2.74
1368.57	1356.96	-11.61
1369.54	1382.78	13.24
1390.93	1389.64	-1.29
1408.08	1405.61	-2.47
1438.48	1409.03	-29.45
1476.08	1439.83	-36.25
1476.77	1471.01	-5.76
1478.18	1471.33	-6.85
1500.71	1492.58	-8.13

1501.67	1493.15	-8.52
1525.97	1511.16	-14.81
1527.29	1519.67	-7.62
1528.70	1520.39	-8.31
1542.81	1521.34	-21.47
1543.27	1521.58	-21.69
3147.73	3163.51	15.78
3148.79	3164.79	16.00
3149.71	3165.30	15.59
3150.77	3166.49	15.72
3151.05	3167.15	16.10
3152.12	3168.35	16.23
3156.00	3171.40	15.40
3156.45	3172.13	15.68
3157.68	3173.40	15.72
3158.25	3174.22	15.97
3160.08	3176.37	16.29
3162.61	3177.91	15.30

Table S13. Harmonic nuclear vibrational frequencies and ΔTAO shifts (in cm^{-1}) for the KS-B3LYP-D3M(BJ)/6-311G(d,p) and TAO-B3LYP-D3M(BJ)/6-311G(d,p) S_0 states of [7]cyclacene.

KS-DFT	TAO-DFT	ΔTAO
103.82	99.45	-4.37
109.48	104.52	-4.96
142.34	143.01	0.67
144.89	144.83	-0.06
273.56	253.42	-20.14
276.33	253.93	-22.40
279.71	277.94	-1.77
280.08	280.07	-0.01
316.61	308.40	-8.21
317.31	309.00	-8.31
351.54	354.82	3.28
368.01	368.96	0.95
369.23	369.83	0.60
404.95	389.42	-15.53
410.52	390.87	-19.65
424.62	403.35	-21.27
425.93	425.72	-0.21
452.14	427.41	-24.73
455.33	428.00	-27.33
462.35	433.40	-28.95
469.87	437.61	-32.26
479.33	474.12	-5.21

480.59	476.77	-3.82
482.30	480.01	-2.29
537.33	531.33	-6.00
540.45	533.60	-6.85
619.57	609.57	-10.00
619.92	609.83	-10.09
630.65	623.18	-7.47
631.94	624.40	-7.54
635.81	634.21	-1.60
638.49	636.34	-2.15
664.68	663.32	-1.36
666.06	664.26	-1.80
696.30	690.44	-5.86
697.20	690.80	-6.40
736.26	741.89	5.63
746.28	755.47	9.19
747.17	756.35	9.18
758.64	757.30	-1.34
765.95	758.19	-7.76
767.20	763.20	-4.00
796.83	792.22	-4.61
797.52	792.95	-4.57
817.07	817.04	-0.03
819.13	817.80	-1.33
822.71	821.54	-1.17
824.29	823.14	-1.15
826.92	827.08	0.16
828.96	828.44	-0.52
858.71	861.47	2.76
868.27	861.57	-6.70
874.60	867.89	-6.71
875.07	868.32	-6.75
876.44	870.84	-5.60
879.94	872.18	-7.76
880.69	872.60	-8.09
899.38	877.66	-21.72
899.84	880.21	-19.63
902.17	893.16	-9.01
905.68	893.54	-12.14
906.51	909.19	2.68
914.63	912.62	-2.01
918.21	913.30	-4.91
971.13	1107.11	135.98
1038.35	1127.94	89.59
1190.23	1150.59	-39.64
1195.39	1155.34	-40.05

1195.96	1189.14	-6.82
1200.21	1192.85	-7.36
1210.87	1204.91	-5.96
1212.43	1206.40	-6.03
1241.96	1234.28	-7.68
1243.43	1235.69	-7.74
1286.79	1282.85	-3.94
1287.07	1283.46	-3.61
1287.08	1286.51	-0.57
1288.15	1286.66	-1.49
1289.05	1287.11	-1.94
1290.23	1288.20	-2.03
1308.27	1292.15	-16.12
1309.60	1293.56	-16.04
1312.32	1313.89	1.57
1319.76	1323.96	4.20
1324.93	1325.86	0.93
1352.50	1346.53	-5.97
1354.31	1348.01	-6.30
1379.46	1350.62	-28.84
1381.31	1372.08	-9.23
1400.50	1372.66	-27.84
1401.11	1375.61	-25.50
1403.15	1376.64	-26.51
1403.57	1384.21	-19.36
1453.74	1452.43	-1.31
1455.71	1454.26	-1.45
1480.93	1483.02	2.09
1491.40	1484.77	-6.63
1493.16	1489.43	-3.73
1493.36	1490.33	-3.03
1494.75	1492.04	-2.71
1498.85	1496.49	-2.36
1500.91	1497.78	-3.13
1562.20	1532.90	-29.30
1565.01	1535.53	-29.48
1577.71	1564.12	-13.59
1579.68	1566.06	-13.62
3153.72	3166.93	13.21
3154.18	3167.25	13.07
3155.15	3168.23	13.08
3155.59	3168.55	12.96
3156.88	3169.98	13.10
3157.71	3170.85	13.14
3158.78	3171.42	12.64
3159.62	3172.28	12.66

3162.51	3174.82	12.31
3162.95	3175.34	12.39
3164.53	3176.92	12.39
3165.14	3177.44	12.30
3165.55	3178.85	13.30
3168.91	3180.33	11.42

Table S14. Harmonic nuclear vibrational frequencies and ΔTAO shifts (in cm^{-1}) for the KS-B3LYP-D3M(BJ)/6-311G(d,p) and TAO-B3LYP-D3M(BJ)/6-311G(d,p) S_0 states of [8]cyclacene.

KS-DFT	TAO-DFT	ΔTAO
84.96	82.04	-2.92
88.28	83.10	-5.18
122.86	118.74	-4.12
127.64	123.38	-4.26
226.50	217.36	-9.14
226.53	217.37	-9.16
275.53	264.65	-10.88
275.54	264.65	-10.89
280.68	273.19	-7.49
280.69	273.21	-7.48
308.33	316.41	8.08
336.43	323.18	-13.25
339.82	334.37	-5.45
341.35	335.63	-5.72
363.03	361.92	-1.11
381.79	371.16	-10.63
396.09	386.44	-9.65
423.29	429.74	6.45
423.74	429.75	6.01
431.02	435.31	4.29
439.20	435.42	-3.78
439.28	441.42	2.14
448.61	443.59	-5.02
450.45	445.80	-4.65
454.65	449.55	-5.10
454.66	449.55	-5.11
462.74	468.02	5.28
489.25	489.74	0.49
489.41	489.83	0.42
492.40	490.63	-1.77
608.71	610.83	2.12
610.85	610.86	0.01
610.88	611.26	0.38
618.27	618.65	0.38

619.02	619.84	0.82
619.60	621.52	1.92
619.64	621.54	1.90
674.34	671.32	-3.02
681.85	680.15	-1.70
681.87	680.16	-1.71
705.23	704.60	-0.63
707.12	705.62	-1.50
747.42	757.24	9.82
754.19	757.38	3.19
754.29	757.40	3.11
766.17	761.42	-4.75
766.19	761.44	-4.75
767.52	767.95	0.43
791.99	793.09	1.10
792.37	793.21	0.84
802.21	793.41	-8.80
824.08	819.35	-4.73
826.08	820.41	-5.67
828.74	820.41	-8.33
828.76	820.69	-8.07
849.25	832.29	-16.96
859.63	845.56	-14.07
859.64	845.57	-14.07
862.34	860.18	-2.16
878.51	873.38	-5.13
883.88	876.40	-7.48
886.24	879.85	-6.39
886.26	883.73	-2.53
886.64	884.91	-1.73
888.73	884.92	-3.81
889.30	885.85	-3.45
889.51	886.86	-2.65
891.11	886.89	-4.22
894.79	890.26	-4.53
894.90	890.34	-4.56
908.19	894.67	-13.52
908.20	894.75	-13.45
920.53	920.94	0.41
925.68	922.32	-3.36
1085.46	1150.45	64.99
1167.18	1175.94	8.76
1167.26	1175.97	8.71
1202.07	1178.23	-23.84
1202.11	1194.63	-7.48
1205.55	1194.64	-10.91

1206.13	1202.58	-3.55
1206.99	1202.83	-4.16
1237.09	1231.61	-5.48
1239.75	1234.04	-5.71
1274.37	1271.74	-2.63
1274.42	1271.76	-2.66
1288.32	1281.74	-6.58
1288.49	1281.85	-6.64
1289.29	1288.77	-0.52
1292.41	1291.32	-1.09
1296.85	1296.05	-0.80
1307.73	1303.52	-4.21
1307.80	1303.54	-4.26
1314.61	1313.38	-1.23
1314.66	1313.44	-1.22
1315.67	1320.16	4.49
1343.42	1340.21	-3.21
1343.87	1357.21	13.34
1349.99	1357.33	7.34
1369.88	1390.98	21.10
1404.99	1391.93	-13.06
1408.08	1395.21	-12.87
1409.47	1403.64	-5.83
1414.26	1403.65	-10.61
1414.29	1408.83	-5.46
1436.70	1430.61	-6.09
1441.09	1431.93	-9.16
1445.10	1439.66	-5.44
1483.06	1446.28	-36.78
1487.79	1484.01	-3.78
1487.85	1484.03	-3.82
1516.26	1511.52	-4.74
1516.28	1512.86	-3.42
1518.27	1515.07	-3.20
1519.45	1515.07	-4.38
1525.76	1520.30	-5.46
1553.24	1541.19	-12.05
1553.28	1541.20	-12.08
1567.72	1547.48	-20.24
1567.75	1547.51	-20.24
1607.33	1596.10	-11.23
1607.60	1596.78	-10.82
3152.11	3166.58	14.47
3153.27	3167.73	14.46
3153.31	3167.74	14.43
3153.32	3167.90	14.58

3154.38	3169.10	14.72
3154.52	3169.11	14.59
3156.06	3170.44	14.38
3156.57	3171.19	14.62
3157.43	3171.94	14.51
3158.18	3172.69	14.51
3160.26	3174.25	13.99
3160.27	3174.26	13.99
3162.08	3176.76	14.68
3162.25	3176.77	14.52
3162.33	3177.25	14.92
3164.87	3178.82	13.95

Table S15. Harmonic nuclear vibrational frequencies and ΔTAO shifts (in cm^{-1}) for the KS-B3LYP-D3M(BJ)/6-311G(d,p) and TAO-B3LYP-D3M(BJ)/6-311G(d,p) S_0 states of [9]cyclacene.

KS-DFT	TAO-DFT	ΔTAO
65.16	64.12	-1.04
65.69	64.35	-1.34
99.37	100.45	1.08
99.90	100.68	0.78
179.18	174.04	-5.14
180.80	174.98	-5.82
227.71	229.63	1.92
228.96	230.40	1.44
232.47	239.60	7.13
236.00	242.41	6.41
279.74	284.72	4.98
295.60	293.08	-2.52
295.77	294.46	-1.31
312.59	298.90	-13.69
313.86	299.18	-14.68
326.71	323.54	-3.17
346.05	350.35	4.30
358.83	351.65	-7.18
360.62	403.49	42.87
406.82	405.00	-1.82
407.18	408.01	0.83
426.68	408.22	-18.46
427.84	420.61	-7.23
432.90	422.88	-10.02
435.91	436.81	0.90
452.51	438.72	-13.79
454.30	439.85	-14.45
458.24	440.25	-17.99

458.82	443.07	-15.75
471.17	457.36	-13.81
471.35	458.91	-12.44
473.00	462.30	-10.70
491.09	487.90	-3.19
493.34	490.09	-3.25
586.09	585.72	-0.37
586.53	585.87	-0.66
605.93	599.86	-6.07
606.23	600.04	-6.19
629.01	619.42	-9.59
629.56	619.93	-9.63
638.00	630.41	-7.59
638.19	630.64	-7.55
665.14	665.65	0.51
665.41	666.03	0.62
695.08	689.33	-5.75
695.87	689.88	-5.99
710.79	705.86	-4.93
711.76	706.67	-5.09
748.22	755.76	7.54
748.26	756.17	7.91
758.00	756.57	-1.43
760.61	757.42	-3.19
761.86	757.56	-4.30
762.18	764.18	2.00
763.32	769.13	5.81
763.67	769.24	5.57
800.25	798.08	-2.17
800.94	798.68	-2.26
801.20	799.29	-1.91
802.49	799.77	-2.72
808.70	820.20	11.50
809.90	820.66	10.76
829.65	826.55	-3.10
832.01	826.84	-5.17
832.70	841.82	9.12
838.64	842.61	3.97
839.65	860.94	21.29
845.71	861.04	15.33
863.14	866.91	3.77
863.51	867.62	4.11
864.53	870.43	5.90
866.44	872.63	6.19
867.59	872.96	5.37
869.22	873.59	4.37

879.24	876.50	-2.74
881.14	877.39	-3.75
885.05	879.86	-5.19
886.39	889.26	2.87
895.20	891.09	-4.11
896.63	892.18	-4.45
909.00	892.33	-16.67
910.10	916.83	6.73
912.82	918.52	5.70
914.56	919.42	4.86
923.42	1126.19	202.77
1026.33	1147.55	121.22
1170.64	1148.06	-22.58
1171.60	1157.39	-14.21
1194.94	1188.03	-6.91
1196.06	1189.12	-6.94
1201.09	1198.23	-2.86
1202.28	1199.22	-3.06
1226.05	1218.38	-7.67
1227.64	1220.00	-7.64
1243.42	1239.78	-3.64
1243.76	1240.23	-3.53
1264.41	1260.03	-4.38
1265.25	1260.80	-4.45
1288.75	1288.91	0.16
1289.97	1290.29	0.32
1293.96	1292.86	-1.10
1294.54	1293.41	-1.13
1299.57	1299.42	-0.15
1300.35	1300.54	0.19
1301.09	1305.21	4.12
1301.55	1305.73	4.18
1305.11	1322.52	17.41
1312.45	1323.02	10.57
1325.21	1324.14	-1.07
1331.69	1340.56	8.87
1332.34	1340.81	8.47
1369.32	1377.69	8.37
1370.01	1377.78	7.77
1381.92	1377.94	-3.98
1382.59	1378.29	-4.30
1412.16	1384.37	-27.79
1412.86	1400.57	-12.29
1413.76	1400.66	-13.10
1413.85	1402.00	-11.85
1415.55	1402.20	-13.35

1415.80	1406.26	-9.54
1459.06	1458.93	-0.13
1460.59	1460.42	-0.17
1496.07	1496.29	0.22
1501.56	1496.84	-4.72
1502.13	1500.81	-1.32
1504.08	1501.58	-2.50
1504.72	1513.82	9.10
1508.03	1513.84	5.81
1508.57	1514.83	6.26
1518.48	1515.53	-2.95
1519.53	1515.99	-3.54
1558.70	1528.79	-29.91
1559.83	1529.40	-30.43
1585.13	1577.63	-7.50
1585.60	1577.94	-7.66
1611.09	1597.82	-13.27
1611.58	1598.18	-13.40
3156.26	3168.60	12.34
3156.97	3169.23	12.26
3157.77	3169.98	12.21
3158.44	3170.54	12.10
3158.45	3170.63	12.18
3158.70	3170.78	12.08
3160.06	3172.04	11.98
3160.28	3172.27	11.99
3161.09	3173.58	12.49
3161.76	3173.81	12.05
3163.60	3175.17	11.57
3163.73	3175.38	11.65
3164.88	3176.48	11.60
3165.17	3176.89	11.72
3167.16	3179.29	12.13
3167.28	3179.59	12.31
3167.75	3179.99	12.24
3170.05	3180.94	10.89

Table S16. Harmonic nuclear vibrational frequencies and ΔTAO shifts (in cm^{-1}) for the KS-B3LYP-D3M(BJ)/6-311G(d,p) and TAO-B3LYP-D3M(BJ)/6-311G(d,p) S_0 states of [10]cyclacene.

KS-DFT	TAO-DFT	ΔTAO
49.33	50.33	1.00
53.79	52.57	-1.22
82.73	83.95	1.22
86.94	86.40	-0.54

145.86	143.99	-1.87
146.12	144.27	-1.85
198.88	198.61	-0.27
200.84	199.40	-1.44
234.98	226.82	-8.16
236.27	228.20	-8.07
254.62	257.47	2.85
264.17	259.06	-5.11
265.47	259.61	-5.86
274.63	272.28	-2.35
275.26	272.73	-2.53
295.59	294.18	-1.41
321.27	317.93	-3.34
322.31	319.22	-3.09
348.88	338.09	-10.79
373.49	370.94	-2.55
374.55	371.57	-2.98
390.84	385.59	-5.25
393.62	394.71	1.09
394.81	394.85	0.04
409.74	399.48	-10.26
428.71	434.00	5.29
443.50	434.48	-9.02
443.92	444.29	0.37
444.46	445.81	1.35
444.59	446.70	2.11
449.91	449.45	-0.46
451.41	450.11	-1.30
453.39	451.24	-2.15
457.62	453.17	-4.45
459.39	456.66	-2.73
469.11	466.36	-2.75
470.52	468.04	-2.48
502.97	501.53	-1.44
566.26	565.98	-0.28
566.72	566.60	-0.12
572.25	571.70	-0.55
572.50	571.81	-0.69
619.91	621.35	1.44
626.09	627.27	1.18
626.72	627.86	1.14
632.29	633.26	0.97
632.43	633.30	0.87
667.18	664.94	-2.24
676.43	675.14	-1.29
677.45	676.05	-1.40

702.64	702.20	-0.44
702.89	702.22	-0.67
704.74	704.24	-0.50
706.28	705.52	-0.76
752.54	757.80	5.26
753.37	758.01	4.64
753.83	758.04	4.21
754.18	758.17	3.99
755.24	758.27	3.03
761.29	759.01	-2.28
761.84	759.85	-1.99
765.17	766.33	1.16
781.32	779.95	-1.37
781.55	780.03	-1.52
802.77	797.92	-4.85
821.47	816.72	-4.75
822.28	817.39	-4.89
827.91	819.50	-8.41
828.38	829.37	0.99
830.59	829.84	-0.75
841.33	831.34	-9.99
841.53	831.69	-9.84
841.95	836.19	-5.76
844.29	838.54	-5.75
853.99	852.79	-1.20
868.95	860.00	-8.95
873.44	863.95	-9.49
874.10	873.75	-0.35
875.51	874.92	-0.59
880.67	877.86	-2.81
885.83	880.33	-5.50
886.92	882.40	-4.52
888.50	884.33	-4.17
889.06	884.81	-4.25
890.81	885.31	-5.50
892.63	886.62	-6.01
897.74	889.00	-8.74
900.74	889.21	-11.53
902.07	899.27	-2.80
902.67	902.34	-0.33
903.66	904.13	0.47
905.89	905.67	-0.22
920.45	920.76	0.31
923.78	923.28	-0.50
1065.88	1146.70	80.82
1138.53	1164.68	26.15

1139.91	1166.81	26.90
1194.49	1172.97	-21.52
1195.08	1187.02	-8.06
1197.17	1189.70	-7.47
1204.48	1201.41	-3.07
1205.61	1202.71	-2.90
1222.41	1214.91	-7.50
1224.22	1216.71	-7.51
1225.26	1222.27	-2.99
1226.47	1223.47	-3.00
1254.42	1250.71	-3.71
1256.29	1252.15	-4.14
1289.78	1288.31	-1.47
1289.83	1288.46	-1.37
1294.56	1294.16	-0.40
1296.06	1295.59	-0.47
1296.31	1296.35	0.04
1297.17	1297.20	0.03
1299.41	1298.88	-0.53
1311.13	1304.68	-6.45
1311.34	1305.27	-6.07
1322.92	1316.28	-6.64
1323.14	1316.48	-6.66
1324.94	1329.20	4.26
1338.26	1336.78	-1.48
1338.61	1337.58	-1.03
1338.90	1355.47	16.57
1339.21	1365.44	26.23
1361.98	1366.01	4.03
1364.58	1388.47	23.89
1415.65	1388.73	-26.92
1415.93	1410.51	-5.42
1423.24	1412.03	-11.21
1425.64	1412.28	-13.36
1426.17	1412.62	-13.55
1426.32	1416.82	-9.50
1426.67	1417.12	-9.55
1449.35	1433.91	-15.44
1451.37	1445.97	-5.40
1455.95	1451.05	-4.90
1465.27	1454.17	-11.10
1489.02	1484.71	-4.31
1489.72	1485.73	-3.99
1519.20	1518.38	-0.82
1519.57	1518.78	-0.79
1523.83	1521.49	-2.34

1526.56	1521.57	-4.99
1526.94	1521.93	-5.01
1528.74	1522.03	-6.71
1529.34	1522.85	-6.49
1552.78	1528.84	-23.94
1553.59	1529.80	-23.79
1558.80	1547.25	-11.55
1559.42	1548.17	-11.25
1614.83	1605.95	-8.88
1615.24	1606.46	-8.78
1625.80	1609.54	-16.26
1626.10	1609.87	-16.23
3154.32	3168.76	14.44
3154.67	3169.05	14.38
3155.64	3170.00	14.36
3155.66	3170.15	14.49
3156.10	3170.48	14.38
3157.04	3171.33	14.29
3157.06	3171.43	14.37
3157.52	3171.61	14.09
3158.56	3172.84	14.28
3158.98	3173.11	14.13
3159.84	3173.95	14.11
3160.48	3174.15	13.67
3160.65	3175.65	15.00
3160.89	3175.85	14.96
3162.42	3175.95	13.53
3163.03	3176.71	13.68
3163.54	3178.85	15.31
3163.59	3178.97	15.38
3164.70	3179.77	15.07
3166.46	3180.26	13.80

Table S17. Harmonic nuclear vibrational frequencies and ΔTAO shifts (in cm^{-1}) for the KS-B3LYP-D3M(BJ)/6-311G(d,p) and TAO-B3LYP-D3M(BJ)/6-311G(d,p) S_0 states of [11]cyclacene.

KS-DFT	TAO-DFT	ΔTAO
37.67	43.47	5.80
43.07	43.66	0.59
72.81	73.85	1.04
74.16	73.99	-0.17
117.87	119.25	1.38
123.91	122.48	-1.43
170.56	172.73	2.17
174.12	175.47	1.35

208.87	210.12	1.25
209.98	211.67	1.69
216.23	220.05	3.82
223.98	220.39	-3.59
225.88	237.21	11.33
232.45	249.12	16.67
246.75	249.59	2.84
248.33	268.41	20.08
281.23	282.47	1.24
282.03	283.02	0.99
312.05	315.15	3.10
326.43	316.10	-10.33
327.30	336.94	9.64
334.73	337.49	2.76
335.29	363.60	28.31
367.10	365.67	-1.43
367.94	372.28	4.34
375.88	372.96	-2.92
376.68	412.93	36.25
429.42	413.31	-16.11
429.70	433.01	3.31
432.80	434.86	2.06
434.66	442.04	7.38
452.07	442.45	-9.62
452.96	444.19	-8.77
454.31	444.56	-9.75
454.53	446.05	-8.48
456.25	448.39	-7.86
457.27	449.32	-7.95
457.81	449.58	-8.23
459.87	451.50	-8.37
463.08	454.32	-8.76
499.73	496.98	-2.75
499.84	497.31	-2.53
541.70	538.72	-2.98
541.74	538.92	-2.82
547.92	549.12	1.20
548.42	549.48	1.06
630.74	625.39	-5.35
630.90	625.53	-5.37
632.24	628.86	-3.38
632.45	629.01	-3.44
637.35	633.04	-4.31
637.83	633.52	-4.31
663.05	664.82	1.77
663.96	666.13	2.17

684.89	685.82	0.93
690.24	686.06	-4.18
693.19	692.96	-0.23
694.06	693.68	-0.38
712.98	709.13	-3.85
715.33	709.50	-5.83
716.84	754.10	37.26
744.24	754.13	9.89
744.39	755.56	11.17
749.09	756.24	7.15
749.30	758.04	8.74
760.56	759.09	-1.47
761.85	760.36	-1.49
762.28	764.42	2.14
768.31	764.59	-3.72
768.90	765.05	-3.85
795.00	802.88	7.88
795.52	802.99	7.47
805.48	803.43	-2.05
805.77	803.95	-1.82
806.72	816.26	9.54
808.34	817.92	9.58
812.18	825.22	13.04
829.80	826.55	-3.25
831.80	835.12	3.32
832.15	835.18	3.03
832.47	835.82	3.35
837.29	836.61	-0.68
837.75	854.73	16.98
856.08	855.70	-0.38
858.41	863.73	5.32
859.55	865.06	5.51
865.68	875.90	10.22
866.49	877.33	10.84
874.19	877.87	3.68
876.25	879.15	2.90
876.86	881.09	4.23
878.14	884.24	6.10
878.30	884.45	6.15
880.34	884.79	4.45
882.37	885.16	2.79
884.50	885.28	0.78
887.21	885.32	-1.89
888.14	886.87	-1.27
894.85	887.02	-7.83
895.67	901.12	5.45

907.66	902.07	-5.59
908.70	919.37	10.67
911.89	920.83	8.94
912.17	921.38	9.21
923.64	1138.43	214.79
1092.70	1156.81	64.11
1159.50	1157.32	-2.18
1159.70	1169.23	9.53
1192.12	1184.44	-7.68
1192.56	1185.02	-7.54
1195.99	1199.98	3.99
1196.84	1201.89	5.05
1201.88	1202.87	0.99
1202.01	1205.13	3.12
1215.72	1209.43	-6.29
1216.76	1209.69	-7.07
1242.33	1240.44	-1.89
1242.53	1241.18	-1.35
1249.56	1268.51	18.95
1250.20	1268.96	18.76
1282.47	1280.89	-1.58
1282.91	1282.29	-0.62
1290.65	1294.52	3.87
1291.44	1294.66	3.22
1295.29	1296.84	1.55
1295.59	1297.03	1.44
1295.88	1297.52	1.64
1296.85	1298.76	1.91
1298.13	1310.38	12.25
1311.25	1312.38	1.13
1311.55	1317.23	5.68
1311.86	1317.63	5.77
1312.16	1330.29	18.13
1313.31	1340.31	27.00
1347.54	1340.72	-6.82
1347.84	1361.30	13.46
1359.45	1361.61	2.16
1378.66	1374.23	-4.43
1378.85	1374.73	-4.12
1385.55	1398.65	13.10
1385.89	1399.26	13.37
1417.18	1405.15	-12.03
1417.35	1406.41	-10.94
1421.33	1406.75	-14.58
1421.59	1414.65	-6.94
1422.35	1414.79	-7.56

1422.48	1415.56	-6.92
1431.64	1415.66	-15.98
1431.73	1423.10	-8.63
1474.75	1461.83	-12.92
1475.15	1462.61	-12.54
1497.49	1500.70	3.21
1497.68	1501.16	3.48
1506.12	1501.87	-4.25
1513.29	1502.95	-10.34
1514.46	1518.29	3.83
1516.69	1518.86	2.17
1516.80	1522.34	5.54
1522.02	1522.66	0.64
1522.70	1523.10	0.40
1533.88	1523.34	-10.54
1534.35	1523.62	-10.73
1560.84	1524.26	-36.58
1561.13	1524.33	-36.80
1587.89	1576.78	-11.11
1588.35	1576.98	-11.37
1621.69	1601.73	-19.96
1621.87	1602.02	-19.85
1627.64	1614.90	-12.74
1628.00	1615.48	-12.52
3157.71	3169.73	12.02
3158.20	3169.83	11.63
3158.90	3170.38	11.48
3159.22	3171.11	11.89
3159.47	3171.19	11.72
3159.73	3171.22	11.49
3160.39	3171.95	11.56
3160.92	3172.61	11.69
3161.12	3172.67	11.55
3161.68	3172.96	11.28
3162.62	3174.27	11.65
3163.08	3174.67	11.59
3163.34	3175.09	11.75
3163.67	3175.37	11.70
3165.52	3176.82	11.30
3165.60	3177.27	11.67
3166.19	3177.50	11.31
3166.30	3177.56	11.26
3167.51	3180.02	12.51
3168.45	3180.57	12.12
3169.53	3180.94	11.41
3170.34	3181.48	11.14

Table S18. Harmonic nuclear vibrational frequencies and ΔTAO shifts (in cm^{-1}) for the KS-B3LYP-D3M(BJ)/6-311G(d,p) and TAO-B3LYP-D3M(BJ)/6-311G(d,p) S_0 states of [12]cyclacene.

KS-DFT	TAO-DFT	ΔTAO
36.70	36.89	0.19
37.56	38.18	0.62
61.84	64.70	2.86
64.36	66.12	1.76
103.68	102.87	-0.81
103.70	102.88	-0.82
153.67	153.98	0.31
153.70	154.00	0.30
191.30	189.04	-2.26
195.97	191.37	-4.60
203.57	199.83	-3.74
203.67	199.98	-3.69
212.94	220.16	7.22
229.93	230.37	0.44
230.67	231.32	0.65
248.36	247.12	-1.24
251.30	250.76	-0.54
256.15	253.63	-2.52
293.68	285.00	-8.68
293.74	285.00	-8.74
309.62	308.85	-0.77
309.63	308.85	-0.78
339.00	338.27	-0.73
340.33	338.75	-1.58
351.23	345.42	-5.81
351.25	346.67	-4.58
359.88	346.68	-13.20
401.66	394.11	-7.55
401.95	399.78	-2.17
403.43	402.31	-1.12
418.09	407.67	-10.42
422.11	436.26	14.15
439.60	436.27	-3.33
440.38	443.46	3.08
446.15	445.83	-0.32
446.62	447.79	1.17
447.63	449.60	1.97
447.67	449.65	1.98

448.70	451.58	2.88
460.39	453.96	-6.43
460.43	454.32	-6.11
462.03	454.35	-7.68
464.36	455.49	-8.87
481.09	478.98	-2.11
481.10	478.98	-2.12
508.48	506.88	-1.60
508.77	508.17	-0.60
509.67	508.45	-1.22
535.58	536.23	0.65
535.60	536.26	0.66
618.76	618.09	-0.67
618.97	618.13	-0.84
627.10	626.71	-0.39
631.03	630.73	-0.30
631.10	630.74	-0.36
636.88	636.69	-0.19
637.25	637.33	0.08
663.74	664.33	0.59
673.74	674.12	0.38
673.76	674.13	0.37
675.99	676.86	0.87
676.35	677.45	1.10
697.24	697.42	0.18
697.41	698.09	0.68
715.96	716.48	0.52
715.97	716.49	0.52
746.36	752.20	5.84
746.93	752.49	5.56
752.59	757.50	4.91
754.00	757.52	3.52
754.88	758.49	3.61
757.85	758.86	1.01
757.88	759.03	1.15
758.71	759.76	1.05
759.54	759.83	0.29
764.08	765.64	1.56
777.79	781.66	3.87
778.16	781.77	3.61
805.11	802.85	-2.26
818.89	815.76	-3.13
818.94	816.91	-2.03
819.88	816.92	-2.96
826.78	825.19	-1.59
826.82	825.22	-1.60

829.45	826.32	-3.13
829.55	826.33	-3.22
839.50	836.58	-2.92
839.93	839.13	-0.80
846.78	844.70	-2.08
847.28	846.57	-0.71
850.04	849.71	-0.33
862.59	864.05	1.46
865.68	864.96	-0.72
867.40	867.30	-0.10
867.41	867.31	-0.10
876.44	874.29	-2.15
876.50	874.37	-2.13
878.31	875.54	-2.77
878.36	875.56	-2.80
880.35	882.34	1.99
885.74	887.19	1.45
889.01	889.19	0.18
889.40	889.97	0.57
889.58	890.75	1.17
894.41	890.83	-3.58
897.69	891.33	-6.36
899.45	891.50	-7.95
899.61	895.37	-4.24
903.32	908.54	5.22
903.34	908.64	5.30
907.43	911.68	4.25
907.80	911.74	3.94
919.95	922.12	2.17
920.78	924.48	3.70
1035.51	1144.86	109.35
1114.37	1162.89	48.52
1114.84	1162.94	48.10
1190.00	1171.84	-18.16
1193.07	1182.16	-10.91
1193.19	1182.23	-10.96
1204.25	1198.43	-5.82
1210.20	1199.03	-11.17
1210.32	1201.03	-9.29
1210.37	1201.85	-8.52
1211.12	1205.68	-5.44
1217.40	1205.74	-11.66
1240.59	1234.41	-6.18
1241.01	1234.51	-6.50
1250.51	1247.03	-3.48
1251.57	1247.62	-3.95

1272.96	1266.15	-6.81
1274.25	1268.66	-5.59
1294.85	1293.73	-1.12
1294.90	1293.75	-1.15
1296.00	1295.18	-0.82
1296.06	1295.20	-0.86
1300.32	1299.67	-0.65
1302.38	1301.63	-0.75
1302.92	1302.46	-0.46
1304.43	1303.34	-1.09
1304.63	1304.31	-0.32
1327.69	1320.63	-7.06
1328.36	1320.68	-7.68
1329.88	1323.67	-6.21
1330.58	1323.71	-6.87
1331.28	1333.92	2.64
1332.27	1358.43	26.16
1332.52	1358.45	25.93
1348.96	1362.63	13.67
1361.69	1374.38	12.69
1361.80	1374.52	12.72
1373.22	1377.84	4.62
1412.47	1379.35	-33.12
1412.96	1409.97	-2.99
1429.72	1409.98	-19.74
1429.94	1413.73	-16.21
1430.02	1419.61	-10.41
1430.26	1419.66	-10.60
1430.79	1420.60	-10.19
1430.91	1422.55	-8.36
1431.02	1422.56	-8.46
1445.74	1435.03	-10.71
1452.56	1453.03	0.47
1460.23	1454.38	-5.85
1463.61	1462.29	-1.32
1487.84	1483.84	-4.00
1487.91	1483.91	-4.00
1520.16	1514.98	-5.18
1520.22	1515.03	-5.19
1522.18	1517.73	-4.45
1526.25	1519.72	-6.53
1526.77	1523.33	-3.44
1528.82	1525.28	-3.54
1529.48	1525.32	-4.16
1539.26	1525.40	-13.86
1539.38	1526.72	-12.66

1542.06	1530.01	-12.05
1542.10	1530.02	-12.08
1548.82	1543.44	-5.38
1548.89	1543.46	-5.43
1609.88	1601.88	-8.00
1610.19	1603.05	-7.14
1624.64	1603.49	-21.15
1625.00	1604.80	-20.20
1638.22	1626.53	-11.69
1638.25	1626.56	-11.69
3155.51	3169.65	14.14
3155.67	3170.01	14.34
3156.15	3170.02	13.87
3156.40	3170.69	14.29
3157.25	3171.09	13.84
3157.36	3171.48	14.12
3157.79	3171.50	13.71
3157.99	3172.08	14.09
3158.59	3172.24	13.65
3159.36	3173.37	14.01
3159.57	3173.38	13.81
3159.82	3173.62	13.80
3159.91	3174.98	15.07
3160.61	3175.01	14.40
3160.76	3175.03	14.27
3162.00	3176.33	14.33
3162.72	3176.98	14.26
3163.15	3177.39	14.24
3163.93	3177.42	13.49
3164.03	3178.29	14.26
3164.35	3179.46	15.11
3164.49	3180.79	16.30
3167.19	3181.10	13.91
3169.32	3181.10	11.78

Table S19. Harmonic nuclear vibrational frequencies and ΔTAO shifts (in cm^{-1}) for the KS-B3LYP-D3M(BJ)/6-311G(d,p) and TAO-B3LYP-D3M(BJ)/6-311G(d,p) S_0 states of [13]cyclacene.

KS-DFT	TAO-DFT	ΔTAO
31.22	31.17	-0.05
31.32	31.27	-0.05
55.43	56.53	1.10
56.13	56.58	0.45
86.71	86.96	0.25
88.07	88.07	0.00

135.71	136.72	1.01
136.61	137.43	0.82
163.23	163.52	0.29
163.51	163.81	0.30
190.77	185.84	-4.93
192.09	186.94	-5.15
195.85	202.36	6.51
215.69	213.57	-2.12
216.05	213.82	-2.23
225.51	225.81	0.30
226.06	226.51	0.45
227.25	228.72	1.47
248.77	249.48	0.71
252.26	252.00	-0.26
284.07	283.31	-0.76
284.57	283.67	-0.90
312.76	313.76	1.00
313.36	315.10	1.74
314.61	315.46	0.85
317.85	317.42	-0.43
330.12	328.46	-1.66
330.80	329.10	-1.70
369.53	368.84	-0.69
370.95	370.34	-0.61
373.08	384.79	11.71
373.10	385.13	12.03
434.80	417.36	-17.44
435.69	418.40	-17.29
437.56	440.00	2.44
438.30	440.78	2.48
440.00	445.25	5.25
441.08	446.16	5.08
442.63	446.82	4.19
445.91	447.45	1.54
447.34	447.63	0.29
452.86	449.66	-3.20
453.14	451.50	-1.64
453.36	452.04	-1.32
456.77	452.06	-4.71
457.53	452.49	-5.04
458.10	455.82	-2.28
458.83	456.38	-2.45
477.33	479.11	1.78
477.59	479.34	1.75
502.91	502.30	-0.61
503.42	502.68	-0.74

522.60	524.51	1.91
522.75	524.74	1.99
597.85	600.04	2.19
598.28	600.36	2.08
625.95	629.05	3.10
626.08	629.25	3.17
631.91	634.73	2.82
632.01	634.79	2.78
634.32	636.97	2.65
634.73	637.43	2.70
654.83	657.68	2.85
655.21	658.10	2.89
662.61	663.81	1.20
664.15	665.37	1.22
681.64	682.04	0.40
682.27	682.58	0.31
704.96	704.61	-0.35
705.19	704.63	-0.56
718.79	718.25	-0.54
719.03	718.44	-0.59
745.80	752.71	6.91
745.99	752.88	6.89
748.09	753.36	5.27
750.68	754.16	3.48
753.97	754.92	0.95
754.41	755.51	1.10
754.85	756.84	1.99
755.65	761.04	5.39
755.96	761.96	6.00
756.47	765.39	8.92
759.98	767.32	7.34
760.30	767.45	7.15
805.80	805.82	0.02
806.26	806.27	0.01
807.34	806.79	-0.55
808.38	806.99	-1.39
823.02	813.60	-9.42
824.86	814.99	-9.87
825.96	823.75	-2.21
826.07	824.27	-1.80
827.31	829.22	1.91
827.44	830.13	2.69
837.34	833.61	-3.73
839.33	834.81	-4.52
845.84	840.91	-4.93
847.15	842.06	-5.09

850.22	851.23	1.01
850.83	851.66	0.83
861.45	855.43	-6.02
863.30	856.69	-6.61
874.10	873.32	-0.78
874.64	873.61	-1.03
876.31	876.31	0.00
876.82	876.54	-0.28
879.66	877.80	-1.86
883.46	878.19	-5.27
884.19	883.08	-1.11
885.57	884.70	-0.87
885.65	885.76	0.11
889.04	885.94	-3.10
889.71	887.25	-2.46
894.65	889.45	-5.20
895.42	891.09	-4.33
898.76	892.24	-6.52
899.08	893.83	-5.25
903.50	897.32	-6.18
903.70	898.38	-5.32
904.87	907.37	2.50
905.09	907.83	2.74
917.67	919.80	2.13
918.07	922.09	4.02
920.82	922.68	1.86
1175.87	1144.37	-31.50
1176.01	1163.63	-12.38
1179.11	1163.85	-15.26
1182.47	1174.02	-8.45
1183.31	1180.58	-2.73
1185.28	1181.10	-4.18
1186.07	1189.25	3.18
1186.31	1191.82	5.51
1196.23	1199.85	3.62
1196.44	1201.36	4.92
1200.23	1203.77	3.54
1200.82	1205.22	4.40
1207.94	1228.42	20.48
1208.90	1228.45	19.55
1229.89	1228.49	-1.40
1230.31	1228.90	-1.41
1242.00	1254.85	12.85
1242.19	1257.16	14.97
1254.35	1284.48	30.13
1255.14	1284.57	29.43

1266.68	1296.05	29.37
1266.98	1296.23	29.25
1296.60	1298.50	1.90
1296.72	1298.66	1.94
1300.74	1298.71	-2.03
1301.30	1298.86	-2.44
1304.07	1298.87	-5.20
1305.40	1300.56	-4.84
1306.34	1306.19	-0.15
1306.43	1307.52	1.09
1316.94	1324.97	8.03
1319.44	1325.02	5.58
1319.61	1326.56	6.95
1323.87	1326.66	2.79
1324.31	1334.32	10.01
1353.14	1351.30	-1.84
1353.57	1351.97	-1.60
1366.40	1370.51	4.11
1366.76	1371.49	4.73
1411.66	1377.58	-34.08
1412.06	1377.80	-34.26
1429.19	1404.11	-25.08
1429.28	1404.25	-25.03
1430.62	1412.74	-17.88
1430.77	1413.66	-17.11
1433.61	1415.46	-18.15
1433.75	1418.45	-15.30
1443.40	1418.60	-24.80
1443.58	1421.90	-21.68
1452.22	1422.18	-30.04
1452.37	1422.76	-29.61
1484.71	1422.93	-61.78
1491.63	1432.61	-59.02
1491.82	1463.20	-28.62
1497.26	1463.38	-33.88
1497.39	1500.23	2.84
1501.18	1500.51	-0.67
1519.89	1501.85	-18.04
1520.58	1502.02	-18.56
1520.92	1507.59	-13.33
1521.29	1507.80	-13.49
1523.28	1525.12	1.84
1533.94	1525.98	-7.96
1534.21	1526.32	-7.89
1541.43	1526.75	-14.68
1541.67	1527.06	-14.61

1542.05	1527.44	-14.61
1542.89	1527.70	-15.19
1578.90	1530.17	-48.73
1579.47	1530.66	-48.81
1591.47	1571.52	-19.95
1591.88	1571.81	-20.07
1628.65	1594.36	-34.29
1628.77	1594.75	-34.02
1633.90	1615.68	-18.22
1633.94	1615.74	-18.20
1648.97	1626.87	-22.10
1649.34	1627.32	-22.02
3156.05	3170.28	14.23
3156.19	3170.42	14.23
3156.57	3170.89	14.32
3156.95	3171.10	14.15
3157.26	3171.68	14.42
3157.56	3171.86	14.30
3158.04	3172.03	13.99
3158.21	3172.37	14.16
3158.35	3172.59	14.24
3158.69	3172.70	14.01
3159.46	3173.58	14.12
3159.83	3174.06	14.23
3160.19	3174.12	13.93
3160.36	3174.28	13.92
3161.61	3175.74	14.13
3161.97	3175.85	13.88
3162.21	3176.04	13.83
3162.48	3176.20	13.72
3163.03	3177.84	14.81
3163.40	3177.93	14.53
3163.79	3178.29	14.50
3163.99	3178.42	14.43
3164.17	3179.41	15.24
3166.67	3180.58	13.91
3167.10	3181.74	14.64
3168.09	3182.16	14.07

Table S20. Harmonic nuclear vibrational frequencies and ΔTAO shifts (in cm^{-1}) for the KS-B3LYP-D3M(BJ)/6-311G(d,p) and TAO-B3LYP-D3M(BJ)/6-311G(d,p) S_0 states of [14]cyclacene.

KS-DFT	TAO-DFT	ΔTAO
26.98	27.06	0.08
28.40	27.80	-0.60

49.55	50.14	0.59
50.36	50.47	0.11
76.99	76.16	-0.83
77.04	76.25	-0.79
123.02	122.84	-0.18
123.35	123.85	0.50
144.42	142.49	-1.93
145.18	143.47	-1.71
179.57	174.99	-4.58
182.62	177.86	-4.76
185.28	188.23	2.95
199.34	199.39	0.05
199.48	199.67	0.19
204.41	204.06	-0.35
204.74	204.72	-0.02
213.90	212.77	-1.13
226.65	222.10	-4.55
226.73	222.18	-4.55
262.49	261.98	-0.51
262.68	262.16	-0.52
289.81	288.37	-1.44
290.14	288.50	-1.64
293.69	292.80	-0.89
294.89	295.57	0.68
309.59	300.88	-8.71
313.12	303.03	-10.09
342.16	341.15	-1.01
343.24	342.38	-0.86
363.99	350.16	-13.83
367.25	363.22	-4.03
370.65	365.26	-5.39
407.03	399.55	-7.48
411.48	411.36	-0.12
422.75	422.78	0.03
424.37	423.46	-0.91
424.85	433.60	8.75
436.84	434.23	-2.61
438.66	435.18	-3.48
441.22	436.61	-4.61
444.27	445.27	1.00
446.83	447.80	0.97
448.04	447.81	-0.23
448.59	447.89	-0.70
457.68	451.41	-6.27
459.42	453.15	-6.27
460.48	454.80	-5.68

461.76	455.43	-6.33
464.45	457.05	-7.40
465.13	458.61	-6.52
465.79	458.85	-6.94
466.84	459.38	-7.46
488.57	486.72	-1.85
489.39	487.69	-1.70
511.64	510.33	-1.31
515.11	515.26	0.15
515.25	515.38	0.13
580.07	578.68	-1.39
580.57	579.00	-1.57
631.26	630.03	-1.23
633.65	632.56	-1.09
634.07	632.95	-1.12
634.73	634.09	-0.64
635.40	634.70	-0.70
638.79	638.12	-0.67
638.85	638.21	-0.64
638.88	639.35	0.47
639.39	639.87	0.48
662.91	662.09	-0.82
671.99	670.34	-1.65
672.69	671.59	-1.10
693.82	691.85	-1.97
694.06	692.09	-1.97
713.70	711.49	-2.21
714.22	711.81	-2.41
720.75	719.02	-1.73
721.69	719.91	-1.78
746.03	752.40	6.37
746.64	752.61	5.97
752.67	752.76	0.09
753.39	753.24	-0.15
753.92	754.23	0.31
754.30	755.93	1.63
754.94	756.48	1.54
756.34	758.44	2.10
756.57	758.66	2.09
757.04	761.09	4.05
758.24	762.34	4.10
763.51	765.33	1.82
787.43	786.95	-0.48
787.53	787.07	-0.46
808.86	804.91	-3.95
809.25	811.25	2.00

809.85	811.99	2.14
815.65	812.92	-2.73
818.74	814.37	-4.37
821.34	815.85	-5.49
821.74	817.43	-4.31
826.51	820.28	-6.23
837.29	832.31	-4.98
839.55	833.56	-5.99
842.25	836.86	-5.39
843.95	838.02	-5.93
848.22	844.38	-3.84
849.15	845.27	-3.88
849.21	846.55	-2.66
861.68	860.64	-1.04
863.07	861.26	-1.81
867.83	861.36	-6.47
869.66	862.63	-7.03
877.11	875.61	-1.50
877.32	877.74	0.42
879.67	878.31	-1.36
880.24	878.58	-1.66
881.40	879.93	-1.47
883.75	880.23	-3.52
885.49	880.67	-4.82
886.24	883.06	-3.18
886.44	885.65	-0.79
888.13	885.84	-2.29
890.55	886.03	-4.52
892.23	887.52	-4.71
894.30	890.24	-4.06
900.33	892.15	-8.18
902.55	892.73	-9.82
903.25	896.18	-7.07
904.66	897.48	-7.18
909.12	908.88	-0.24
910.50	911.24	0.74
911.06	914.64	3.58
912.06	915.78	3.72
919.80	920.09	0.29
923.57	924.61	1.04
984.29	1146.37	162.08
1085.80	1164.66	78.86
1087.09	1166.07	78.98
1179.96	1173.99	-5.97
1185.39	1177.85	-7.54
1185.73	1179.53	-6.20

1200.14	1184.63	-15.51
1202.72	1186.89	-15.83
1203.72	1199.82	-3.90
1205.82	1202.04	-3.78
1206.08	1203.16	-2.92
1207.90	1205.13	-2.77
1220.36	1217.33	-3.03
1220.49	1217.71	-2.78
1228.43	1222.23	-6.20
1230.94	1224.77	-6.17
1250.81	1246.97	-3.84
1252.79	1249.35	-3.44
1270.84	1267.95	-2.89
1271.15	1268.30	-2.85
1286.79	1282.23	-4.56
1287.72	1283.06	-4.66
1296.81	1296.20	-0.61
1296.96	1296.59	-0.37
1297.12	1297.35	0.23
1297.85	1297.94	0.09
1298.09	1298.30	0.21
1298.58	1298.90	0.32
1300.51	1300.25	-0.26
1309.63	1309.99	0.36
1310.54	1310.43	-0.11
1310.93	1311.08	0.15
1311.48	1311.38	-0.10
1322.59	1328.57	5.98
1323.68	1328.84	5.16
1332.26	1332.43	0.17
1333.67	1332.66	-1.01
1337.97	1336.34	-1.63
1338.00	1367.36	29.36
1339.47	1370.72	31.25
1339.77	1371.71	31.94
1376.98	1375.12	-1.86
1378.27	1375.54	-2.73
1378.69	1382.63	3.94
1404.14	1383.28	-20.86
1404.85	1402.00	-2.85
1429.11	1402.81	-26.30
1430.07	1416.55	-13.52
1430.79	1418.88	-11.91
1431.90	1419.37	-12.53
1431.93	1423.48	-8.45
1432.13	1423.94	-8.19

1432.15	1424.77	-7.38
1432.68	1425.54	-7.14
1433.09	1425.95	-7.14
1433.14	1435.89	2.75
1456.01	1456.08	0.07
1464.22	1457.72	-6.50
1468.65	1466.63	-2.02
1485.87	1481.02	-4.85
1486.41	1481.20	-5.21
1519.99	1501.48	-18.51
1520.71	1502.04	-18.67
1521.04	1514.39	-6.65
1522.89	1515.20	-7.69
1523.19	1525.63	2.44
1523.23	1527.08	3.85
1523.79	1527.37	3.58
1527.40	1527.75	0.35
1528.11	1528.13	0.02
1541.40	1531.41	-9.99
1541.52	1531.47	-10.05
1542.80	1531.77	-11.03
1543.22	1532.20	-11.02
1543.37	1537.82	-5.55
1543.73	1538.34	-5.39
1601.52	1590.82	-10.70
1602.05	1590.99	-11.06
1616.28	1594.28	-22.00
1616.42	1594.97	-21.45
1636.79	1626.81	-9.98
1637.02	1626.98	-10.04
1644.15	1628.71	-15.44
1644.50	1629.13	-15.37
3156.40	3170.47	14.07
3156.60	3170.61	14.01
3156.78	3170.86	14.08
3157.42	3171.25	13.83
3157.91	3171.93	14.02
3157.95	3171.94	13.99
3158.17	3172.11	13.94
3158.28	3172.34	14.06
3158.90	3172.61	13.71
3158.99	3172.82	13.83
3159.41	3173.21	13.80
3159.62	3173.26	13.64
3160.71	3174.28	13.57
3161.13	3174.29	13.16

3161.30	3174.98	13.68
3161.65	3175.01	13.36
3162.27	3176.06	13.79
3162.37	3176.44	14.07
3163.41	3176.67	13.26
3163.44	3176.75	13.31
3163.56	3178.19	14.63
3164.04	3178.34	14.30
3164.31	3178.75	14.44
3164.53	3178.79	14.26
3164.72	3179.67	14.95
3164.96	3181.07	16.11
3166.70	3182.29	15.59
3167.81	3182.98	15.17

7. Atomic Cartesian Coordinates

7.1 TAO-DFT Ground States

Table S21. Cartesian coordinates (in Å) for the TAO-DFT S_0 state of [6]cyclacene.

H	2.1308438998	-1.0142576184	2.4848716065
C	2.1679281396	-1.0320839608	1.4022608419
C	2.1679281137	-1.0320839477	-1.4022608443
C	2.3838445585	0.1900940830	0.7236367564
C	2.3838445619	0.1900940827	-0.7236367669
H	2.1308438971	-1.0142576157	-2.4848715902
H	0.1870512403	-2.3582411975	2.4849297274
C	0.1900644232	-2.3970254164	1.4021215567
C	0.1900644216	-2.3970253826	-1.4021215614
C	1.3563313731	-1.9714274422	0.7236745215
C	1.3563313756	-1.9714274437	-0.7236745331

H	0.1870512411	-2.3582411865	-2.4849297133
C	1.9793163510	1.3637658044	1.4022410968
C	1.9793163252	1.3637657852	-1.4022411005
H	1.9479420278	1.3420345837	-2.4850510771
H	1.9479420322	1.3420345896	2.4850510921
C	-1.0288739004	-2.1621874663	0.7234722368
C	-1.0288739008	-2.1621874668	-0.7234722493
C	1.0288738911	2.1621874663	0.7234722367
C	1.0288738914	2.1621874668	-0.7234722492
H	-2.1308438836	1.0142576108	-2.4848715971
C	-2.1679280968	1.0320839397	-1.4022608469
C	-2.1679281227	1.0320839529	1.4022608446
C	-1.3563314001	1.9714274695	-0.7236745332
C	-1.3563313977	1.9714274680	0.7236745216
H	-2.1308438864	1.0142576135	2.4848716134
H	-1.9479420133	-1.3420345937	-2.4850510812
C	-1.9793163166	-1.3637657804	-1.4022411020
C	-1.9793163425	-1.3637657996	1.4022410982
C	-2.3838445997	-0.1900940814	-0.7236367670
C	-2.3838445964	-0.1900940816	0.7236367566
H	-1.9479420177	-1.3420345996	2.4850510962
C	-0.1900644202	2.3970253742	-1.4021215627
C	-0.1900644219	2.3970254080	1.4021215579
H	-0.1870512232	2.3582411968	2.4849297305

H	-0.1870512240	2.3582411859	-2.4849297165

Table S22. Cartesian coordinates (in Å) for the TAO-DFT S_0 state of [7]cyclacene.

C	-0.3424522213	2.7622595883	-1.4074969200
C	-0.3424522213	2.7622595884	1.4074969200
C	0.8858367192	2.6253317626	-0.7282530568
C	0.8858367192	2.6253317626	0.7282530568
H	-0.3370044408	2.7172264310	2.4899329214
H	-2.3365737249	1.4371590713	-2.4898604507
C	-2.3741942803	1.4604724262	-1.4073957289
C	-2.3741942804	1.4604724262	1.4073957289
C	-1.5033666280	2.3383566041	-0.7282902704
C	-1.5033666280	2.3383566041	0.7282902704
H	-2.3365737249	1.4371590714	2.4898604507
C	1.9415541310	1.9827098902	-1.4074322898
C	1.9415541310	1.9827098902	1.4074322898
H	1.9078072461	1.9482607821	2.4896477809
H	1.9078072461	1.9482607821	-2.4896477808
C	-2.7573882163	0.2852645669	-0.7283232800
C	-2.7573882163	0.2852645669	0.7283232800
C	2.6036772428	0.9394445878	-0.7282006531
C	2.6036772428	0.9394445878	0.7282006531
C	-2.6107121994	-0.9416274771	1.4075617501
C	-2.6107121994	-0.9416274771	-1.4075617501

C	2.7659953928	-0.2856067275	1.4074745748
C	2.7659953928	-0.2856067274	-1.4074745748
C	-0.8881021076	-2.6322842209	-1.4073956180
C	-0.8881021076	-2.6322842209	1.4073956180
C	-1.9361931328	-1.9770344291	-0.7283668170
C	-1.9361931328	-1.9770344291	0.7283668170
H	-2.5671808183	-0.9260273777	2.4898468572
H	-2.5671808183	-0.9260273778	-2.4898468572
H	2.7206005465	-0.2809811476	2.4898929656
C	2.3674315038	-1.4555115962	-0.7282715747
C	2.3674315038	-1.4555115962	0.7282715747
C	1.5084195695	-2.3448942285	-1.4074572009
C	1.5084195695	-2.3448942285	1.4074572009
H	-0.8735017127	-2.5888933133	2.4897168108
H	-0.8735017127	-2.5888933133	-2.4897168108
H	-0.3370044408	2.7172264310	-2.4899329214
C	0.3418060946	-2.7533900520	-0.7283435489
C	0.3418060947	-2.7533900520	0.7283435489
H	2.7206005465	-0.2809811476	-2.4898929656
H	1.4842183633	-2.3075669674	2.4899128454
H	1.4842183633	-2.3075669673	-2.4899128454

Table S23. Cartesian coordinates (in Å) for the TAO-DFT S_0 state of [8]cyclacene.

H	-2.8714523949	1.2451171941	2.4876346948

C	-2.9039169611	1.2590369821	1.4046654790
C	-2.9039163631	1.2590367236	-1.4046655609
C	-3.1551460297	0.0516987502	0.7272241891
C	-3.1551458389	0.0516987475	-0.7272244048
H	-2.8714518007	1.2451169369	-2.4876343964
C	-2.9440080410	-1.1632246359	1.4046149084
C	-2.9440074320	-1.1632243954	-1.4046149918
H	-2.9112500423	-1.1503102432	-2.4875792521
H	-2.9112506476	-1.1503104829	2.4875795491
C	-2.2677409237	-2.1945695271	0.7272478935
C	-2.2677407859	-2.1945693942	-0.7272481102
H	1.1503105550	-2.9112498517	-2.4875794747
C	1.1632247508	-2.9440073706	-1.4046150692
C	1.1632249904	-2.9440079776	1.4046149874
C	-0.0516986881	-3.1551458838	-0.7272245083
C	-0.0516986914	-3.1551460734	0.7272242936
H	1.1503107930	-2.9112504536	2.4875797732
H	2.8714519535	-1.2451168157	-2.4876345702
C	2.9039166201	-1.2590365938	-1.4046656289
C	2.9039172185	-1.2590368524	1.4046655471
C	2.1945697167	-2.2677404866	-0.7272481255
C	2.1945698489	-2.2677406231	0.7272479101
H	2.8714525482	-1.2451170730	2.4876348686
C	-1.2590369977	-2.9039164653	-1.4046656756

C	-1.2590372576	-2.9039170635	1.4046655935
H	-1.2451174585	-2.8714523072	2.4876350052
H	-1.2451171988	-2.8714517129	-2.4876347069
C	-2.1945695305	2.2677404910	-0.7272480562
C	-2.1945696626	2.2677406275	0.7272478406
C	-1.1632245666	2.9440070753	-1.4046149400
C	-1.1632248061	2.9440076823	1.4046148580
H	-1.1503106848	2.9112503144	2.4875794441
H	-1.1503104468	2.9112497127	-2.4875791458
C	0.0516986518	3.1551457291	-0.7272244229
C	0.0516986551	3.1551459187	0.7272242081
H	2.9112505343	1.1503106477	2.4875794669
C	2.9440078672	1.1632248358	1.4046148763
C	2.9440072581	1.1632245953	-1.4046149597
C	2.2677407269	2.1945695926	0.7272478418
C	2.2677405891	2.1945694598	-0.7272480585
H	2.9112499291	1.1503104080	-2.4875791700
C	3.1551460589	-0.0516985247	0.7272242019
C	3.1551458681	-0.0516985219	-0.7272244175
C	1.2590370148	2.9039168376	1.4046654791
C	1.2590367551	2.9039162398	-1.4046655612
H	1.2451170436	2.8714516167	-2.4876344160
H	1.2451173030	2.8714522107	2.4876347142

Table S24. Cartesian coordinates (in Å) for the TAO-DFT S_0 state of [9]cyclacene.

H	-3.5391300716	-0.0166145237	2.4885253148
C	-3.5729552364	-0.0167336657	1.4054768683
C	-3.5729552359	-0.0167336653	-1.4054768687
C	-3.3423057841	-1.2277093256	0.7275968855
C	-3.3423057836	-1.2277093254	-0.7275968863
H	-3.5391300711	-0.0166145229	-2.4885253151
C	-2.7194806561	-2.2915686730	1.4056237111
C	-2.7194806551	-2.2915686724	-1.4056237118
H	-2.6938627928	-2.2695495107	-2.4885453971
H	-2.6938627942	-2.2695495115	2.4885453964
C	-1.7598345095	-3.0641435357	0.7274410028
C	-1.7598345091	-3.0641435355	-0.7274410032
H	1.7742609216	-3.0239721543	-2.4885893147
C	1.7923020449	-3.0553152481	-1.4057314475
C	1.7923020447	-3.0553152475	1.4057314482
C	0.6315842269	-3.4690477672	-0.7275685612
C	0.6315842267	-3.4690477666	0.7275685617
H	1.7742609210	-3.0239721531	2.4885893154
H	3.3198683575	1.2194855817	-2.4885071507
C	3.3497779720	1.2306251170	-1.4054713980
C	3.3497779730	1.2306251175	1.4054713974
H	3.3198683588	1.2194855825	2.4885071502
C	-0.6006429899	-3.4819095996	-1.4054191418

C	-0.6006429905	-3.4819095996	1.4054191419
H	-0.5952546956	-3.4476613683	2.4883160894
H	-0.5952546946	-3.4476613687	-2.4883160893
C	-3.3530258649	1.1962149303	-0.7275975790
C	-3.3530258645	1.1962149300	0.7275975790
C	-2.7397915637	2.2656347426	-1.4056209095
C	-2.7397915633	2.2656347420	1.4056209100
H	-2.7138642339	2.2437542483	2.4885381224
H	-2.7138642347	2.2437542496	-2.4885381219
C	-1.7873697196	3.0470731825	-0.7274397497
C	-1.7873697191	3.0470731820	0.7274397502
H	1.7470637856	3.0410577860	2.4885917200
C	1.7647077332	3.0723822244	1.4057240048
C	1.7647077324	3.0723822239	-1.4057240051
C	0.6000423635	3.4748934447	0.7275722064
C	0.6000423632	3.4748934447	-0.7275722064
H	1.7470637845	3.0410577855	-2.4885917203
C	2.7139293967	2.2868167743	0.7275947404
C	2.7139293962	2.2868167741	-0.7275947409
C	-0.6322378235	3.4758846948	1.4054242250
C	-0.6322378240	3.4758846951	-1.4054242246
H	-0.6265326055	3.4417188341	-2.4883183815
H	-0.6265326047	3.4417188332	2.4883183820
H	3.3304159371	-1.1881884213	2.4884967261

C	3.3604208080	-1.1990426561	1.4054636862
C	3.3604208075	-1.1990426564	-1.4054636861
C	3.5625791459	0.0166951605	0.7275788121
C	2.7341317998	-2.2609324218	0.7275929145
C	2.7341317997	-2.2609324220	-0.7275929140
C	3.5625791456	0.0166951603	-0.7275788124
H	3.3304159367	-1.1881884220	-2.4884967259

Table S25. Cartesian coordinates (in Å) for the TAO-DFT S_0 state of [10]cyclacene.

H	-3.8516107480	0.6388666302	2.4882487890
C	-3.8814155531	0.6433554853	1.4051179855
C	-3.8814155288	0.6433554864	-1.4051179878
C	-3.8849008418	-0.5872430631	0.7282256277
C	-3.8849008305	-0.5872430588	-0.7282256394
H	-3.8516107214	0.6388666346	-2.4882487779
C	-3.5234907568	-1.7635319327	1.4051571619
C	-3.5234907261	-1.7635319145	-1.4051571717
H	-3.4966888817	-1.7500672907	-2.4883339911
H	-3.4966889202	-1.7500673146	2.4883339949
C	-2.8028891404	-2.7613031586	0.7282756321
C	-2.8028891293	-2.7613031480	-0.7282756474
H	0.5810517815	-3.8619428073	-2.4882558420
C	0.5851091956	-3.8916652466	-1.4051117776
C	0.5851091955	-3.8916652729	1.4051117786

C	-0.6454085589	-3.8760754721	-0.7282151121
C	-0.6454085626	-3.8760754806	0.7282151038
H	0.5810517780	-3.8619428364	2.4882558563
H	3.4966883927	1.7500672176	-2.4883334859
C	3.5234901119	1.7635317784	-1.4051569710
C	3.5234901460	1.7635317981	1.4051569618
H	3.4966884356	1.7500672446	2.4883334902
C	-1.8159620204	-3.4965802632	-1.4051924148
C	-1.8159620398	-3.4965802932	1.4051924069
H	-1.8019320811	-3.4695510768	2.4883452138
H	-1.8019320544	-3.4695510403	-2.4883452082
C	-3.4850385428	1.8082468863	-0.7282198045
C	-3.4850385469	1.8082468865	0.7282198009
C	-2.7616196384	2.8036739558	-1.4052317425
C	-2.7616196516	2.8036739682	1.4052317459
H	-2.7421244876	2.7839079909	2.4884401888
H	-2.7421244762	2.7839079813	-2.4884401722
C	-1.7558721580	3.5128445356	-0.7282412967
C	-1.7558721581	3.5128445388	0.7282412945
H	1.8019320074	3.4695505838	2.4883447105
C	1.8159619009	3.4965796757	1.4051922073
C	1.8159618815	3.4965796470	-1.4051922155
C	0.6454087813	3.8760750598	0.7282149882
C	0.6454087767	3.8760750499	-0.7282149974

H	1.8019319816	3.4695505485	-2.4883447052
C	2.8028887701	2.7613027883	0.7282754887
C	2.8028887582	2.7613027775	-0.7282755040
C	-0.5851087190	3.8916650142	1.4051116531
C	-0.5851087204	3.8916649925	-1.4051116530
H	-0.5810513160	3.8619426433	-2.4882555308
H	-0.5810513115	3.8619426655	2.4882555441
H	2.7421248755	-2.7839075977	2.4884401874
C	2.7616200361	-2.8036735798	1.4052317459
C	2.7616200214	-2.8036735657	-1.4052317408
C	1.7558726440	-3.5128444428	0.7282413397
C	1.7558726417	-3.5128444360	-0.7282413402
H	2.7421248616	-2.7839075846	-2.4884401689
H	3.8516105814	-0.6388661630	2.4882484746
C	3.8814153004	-0.6433550076	1.4051178603
C	3.8814152737	-0.6433550082	-1.4051178607
C	3.8849004303	0.5872432829	0.7282255128
C	3.4850386498	-1.8082464025	0.7282197569
C	3.4850386423	-1.8082464006	-0.7282197579
C	3.8849004180	0.5872432786	-0.7282255230
H	3.8516105507	-0.6388661673	-2.4882484616

Table S26. Cartesian coordinates (in Å) for the TAO-DFT S_0 state of [11]cyclacene.

H	-3.9675158966	-1.6809832975	2.4880188289

C	-3.9908116317	-1.6909853658	1.4047142354
C	-3.9908116451	-1.6909853663	-1.4047142307
C	-3.3421805470	-2.7369485754	0.7276978633
C	-3.3421805490	-2.7369485740	-0.7276978613
H	-3.9675159144	-1.6809832954	-2.4880188292
C	-2.4356344019	-3.5683540737	1.4050371459
C	-2.4356344061	-3.5683540746	-1.4050371498
H	-2.4193883094	-3.5442995329	-2.4881594956
H	-2.4193883051	-3.5442995363	2.4881594870
C	-1.3263894949	-4.1009603025	0.7277757530
C	-1.3263894952	-4.1009603020	-0.7277757566
C	1.1070664476	-4.1652979108	-0.7276917772
C	1.1070664464	-4.1652979100	0.7276917743
C	-0.1143951327	-4.3142153522	-1.4046912344
C	-0.1143951331	-4.3142153458	1.4046912284
H	-0.1137780328	-4.2877636354	2.4879905648
H	-0.1137780319	-4.2877636385	-2.4879905756
C	-4.3008643266	-0.5001996707	-0.7277400550
C	-4.3008643179	-0.5001996713	0.7277400657
C	-4.2759386039	0.7301015246	-1.4048851025
C	-4.2759385799	0.7301015210	1.4048851125
H	-4.2487366671	0.7259276233	2.4880783263
H	-4.2487366998	0.7259276283	-2.4880783214
C	-3.8896007809	1.8982937658	-0.7277859052

C	-3.8896007724	1.8982937640	0.7277859154
H	-1.1025892450	4.1455151622	2.4881255951
C	-1.1089706845	4.1713505268	1.4049065996
C	-1.1089706876	4.1713505252	-1.4049066057
C	-2.2398498842	3.6868749751	0.7276980336
C	-2.2398498866	3.6868749735	-0.7276980333
H	-1.1025892490	4.1455151534	-2.4881256058
C	-3.1996100629	2.9173708647	1.4049908968
C	-3.1996100757	2.9173708686	-1.4049908934
H	-3.1806870207	2.9000184878	-2.4882371251
H	-3.1806870033	2.9000184856	2.4882371236
H	3.3270600941	2.7243734171	2.4882418644
C	3.3481262263	2.7417977032	1.4050542771
C	3.3481262387	2.7417977060	-1.4050542747
C	2.4312533518	3.5615879948	0.7276749522
C	2.4312533538	3.5615879929	-0.7276749528
H	3.3270601105	2.7243734175	-2.4882418668
C	1.3282738661	4.1069070577	1.4047932182
C	1.3282738686	4.1069070558	-1.4047932247
H	1.3208602902	4.0829306443	-2.4880860092
H	1.3208602877	4.0829306535	2.4880859979
C	0.1140030520	4.3053390596	0.7278487941
C	0.1140030518	4.3053390559	-0.7278487998
C	3.9844591661	1.6883249076	-0.7278301097

C	3.9844591578	1.6883249065	0.7278301189
C	4.3086790310	0.5012320532	-1.4048135061
C	4.3086790074	0.5012320514	1.4048135155
H	4.2819664094	0.4984636493	2.4880559057
H	4.2819664414	0.4984636516	-2.4880559014
C	4.2679437167	-0.7286663579	-0.7276724078
C	4.2679437081	-0.7286663576	0.7276724183
H	2.2288500007	-3.6694391655	2.4880866658
C	2.2436345621	-3.6940607207	1.4049467408
C	2.2436345678	-3.6940607238	-1.4049467433
C	3.1939662347	-2.9126160897	0.7277292449
C	3.1939662373	-2.9126160892	-0.7277292418
H	2.2288500075	-3.6694391656	-2.4880866731
C	3.8957213449	-1.9014726093	1.4047586744
C	3.8957213586	-1.9014726114	-1.4047586692
H	3.8716695533	-1.8895714460	-2.4880013897
H	3.8716695349	-1.8895714453	2.4880013900

Table S27. Cartesian coordinates (in Å) for the TAO-DFT S_0 state of [12]cyclacene.

H	-0.4009508625	4.6716922357	-2.4883866808
C	-0.4027613683	4.6959735552	-1.4050341818
C	-0.4027612904	4.6959730760	1.4050342276
C	0.8250235597	4.6327041499	-0.7284114072
C	0.8250235476	4.6327039796	0.7284114944

H	-0.4009507415	4.6716916009	2.4883865232
C	1.9990902475	4.2680144593	-1.4049479267
C	1.9990900954	4.2680140823	1.4049479131
H	1.9890120474	4.2465643524	2.4883042102
H	1.9890122294	4.2465648345	-2.4883044262
C	3.0311166692	3.5994659371	-0.7284352478
C	3.0311165795	3.5994658300	0.7284352970
C	4.4256993972	1.6022226863	0.7285582265
C	4.4256995713	1.6022227287	-0.7285581420
C	3.8643839704	2.6960445217	1.4053050487
C	3.8643843002	2.6960447255	-1.4053050661
H	3.8426780061	2.6808881436	-2.4885345860
H	3.8426775846	2.6808878958	2.4885343662
C	-1.6022216206	4.4256998651	0.7285581117
C	-1.6022217082	4.4257000630	-0.7285579611
C	-2.6960432148	3.8643845711	1.4053050010
C	-2.6960435264	3.8643849866	-1.4053049037
H	-2.6808871924	3.8426787673	-2.4885342441
H	-2.6808867586	3.8426782022	2.4885341367
C	-3.5994646664	3.0311173439	0.7284353797
C	-3.5994648439	3.0311174932	-0.7284351977
H	-4.6716918165	-0.4009504929	-2.4883874971
C	-4.6959735532	-0.4027610084	-1.4050344729
C	-4.6959730666	-0.4027610066	1.4050345179

C	-4.6327035619	0.8250242316	-0.7284115363
C	-4.6327033596	0.8250241788	0.7284116880
H	-4.6716911685	-0.4009505079	2.4883873386
C	-4.2680136324	1.9990911688	-1.4049480102
C	-4.2680131514	1.9990909240	1.4049481109
H	-4.2465632372	1.9890126232	2.4883046391
H	-4.2465638960	1.9890129676	-2.4883047431
H	0.4009509302	-4.6716930474	-2.4883879050
C	0.4027614831	-4.6959749207	-1.4050345985
C	0.4027614053	-4.6959744418	1.4050346443
C	-0.8250239889	-4.6327050528	-0.7284116675
C	-0.8250239768	-4.6327048826	0.7284117548
H	0.4009508092	-4.6716924129	2.4883877474
C	-1.9990912209	-4.2680152814	-1.4049482862
C	-1.9990910688	-4.2680149046	1.4049482727
H	-1.9890127498	-4.2465647634	2.4883052656
H	-1.9890129318	-4.2465652453	-2.4883054815
C	-3.0311176656	-3.5994659940	-0.7284354397
C	-3.0311175758	-3.5994658869	0.7284354889
C	-3.8643850205	-2.6960441114	1.4053052605
C	-3.8643853503	-2.6960443152	-1.4053052778
H	-3.8426787656	-2.6808876758	-2.4885352005
C	1.6022222089	-4.4257007058	0.7285583789
C	1.6022222965	-4.4257009036	-0.7285582283

C	2.6960443286	-3.8643852225	1.4053053726
C	2.6960446401	-3.8643856380	-1.4053052753
H	2.6808879683	-3.8426790543	-2.4885353162
H	2.6808875345	-3.8426784893	2.4885352089
C	3.5994656563	-3.0311172121	0.7284355746
C	3.5994658338	-3.0311173613	-0.7284353925
H	4.6716916773	0.4009514546	-2.4883874960
C	4.6959734302	0.4027621129	-1.4050344758
C	4.6959729436	0.4027621112	1.4050345209
C	4.6327039568	-0.8250232553	-0.7284116053
C	4.6327037545	-0.8250232025	0.7284117570
H	4.6716910293	0.4009514696	2.4883873375
C	4.2680146085	-1.9990905690	-1.4049482173
C	4.2680141275	-1.9990903242	1.4049483180
H	4.2465639166	-1.9890120173	2.4883052512
H	4.2465645754	-1.9890123617	-2.4883053550
C	-4.4256999248	-1.6022217949	0.7285582956
C	-4.4257000989	-1.6022218374	-0.7285582112
H	-3.8426783439	-2.6808874279	2.4885349809

Table S28. Cartesian coordinates (in Å) for the TAO-DFT S_0 state of [13]cyclacene.

H	-4.8880450498	1.4121558226	2.4881299014
C	-4.9094248063	1.4179149081	1.4047368921
C	-4.9094243236	1.4179151183	-1.4047381781

C	-5.0948679820	0.2027259804	0.7281669095
C	-5.0948677216	0.2027261020	-0.7281684685
H	-4.8880442129	1.4121562076	-2.4881311408
C	-4.9964736800	-1.0225896802	1.4047294478
C	-4.9964731666	-1.0225894157	-1.4047311817
H	-4.9741966015	-1.0183703836	-2.4881050716
H	-4.9741974734	-1.0183708509	2.4881033843
C	-4.5960470906	-2.1848322057	0.7281997145
C	-4.5960468157	-2.1848320527	-0.7282015466
C	-3.0460304440	-4.0678764825	-0.7280155211
C	-3.0460306857	-4.0678766355	0.7280138183
C	-3.9393477578	-3.2242114243	-1.4049195030
C	-3.9393482680	-3.2242117409	1.4049176921
H	-3.9216154919	-3.2096832913	2.4882585568
H	-3.9216146237	-3.2096827528	-2.4882603214
C	3.6913630554	-3.5341678441	0.7280467315
C	3.6913632623	-3.5341676026	-0.7280460657
C	4.4318217746	-2.5527155947	1.4046468217
C	4.4318222184	-2.5527151898	-1.4046456179
C	2.7430516191	-4.3162709602	-1.4047744738
C	2.7430512290	-4.3162714621	1.4047746258
H	2.7303067729	-4.2962638272	-2.4880826773
C	1.6291033752	-4.8360915292	-0.7281046717
C	1.6291031504	-4.8360917659	0.7281043081

H	-1.9766589389	-4.6690286147	2.4879373427
C	-1.9848172335	-4.6884678147	1.4045213780
C	-1.9848167582	-4.6884674611	-1.4045228600
C	-0.8026294480	-5.0253380269	0.7280468121
C	-0.8026292036	-5.0253378131	-0.7280479999
H	-1.9766581024	-4.6690280033	-2.4879387802
C	0.4252209712	-5.0850436986	1.4046104544
C	0.4252214198	-5.0850432588	-1.4046112531
H	0.4237133836	-5.0629851052	-2.4880111907
H	0.4237125916	-5.0629858545	2.4880104361
H	0.8015264890	5.0124085823	-2.4880556781
C	0.8047435564	5.0346515682	-1.4046723077
C	0.8047430393	5.0346516252	1.4046735240
H	0.8015255606	5.0124086223	2.4880569339
C	-0.4237806736	5.0788934229	0.7281398787
C	-0.4237803913	5.0788933869	-0.7281391413
C	-1.6297702581	4.8408782522	1.4046792614
C	-1.6297696905	4.8408782324	-1.4046789840
H	-1.6232467262	4.8203585073	-2.4880913482
H	-1.6232477249	4.8203585003	2.4880916690
C	-2.7375193125	4.3079834812	0.7280725733
C	-2.7375190124	4.3079834936	-0.7280727637
C	-4.4251753006	2.5477912251	-0.7281354152
C	-4.4251755512	2.5477911310	0.7281344447

C	-3.6941234735	3.5361935107	-1.4049075545
C	-3.6941240254	3.5361934151	1.4049069598
H	-3.6784554371	3.5211989439	2.4882952298
H	-3.6784545038	3.5211991545	-2.4882957795
H	3.0364983169	4.0566299694	2.4882086044
C	3.0507217877	4.0755528183	1.4048984131
C	3.0507223327	4.0755529581	-1.4048964057
C	1.9835235596	4.6858206964	0.7281873735
C	1.9835238238	4.6858207064	-0.7281857381
H	3.0364992956	4.0566302438	-2.4882065644
C	3.9335639456	3.2208615860	-0.7280213321
C	3.9335636205	3.2208614539	0.7280235655
C	4.5994090277	2.1873083391	-1.4044987905
C	4.5994084092	2.1873080586	1.4045011308
H	4.5800508073	2.1780866165	2.4878981757
H	4.5800519305	2.1780871387	-2.4878958042
C	4.9866231930	1.0206296922	-0.7280158292
C	4.9866228512	1.0206295295	0.7280180740
C	4.9021149260	-1.4169635587	0.7280541153
C	4.9021151885	-1.4169633727	-0.7280524632
C	5.0989688857	-0.2034919891	1.4046075901
C	5.0989694748	-0.2034916647	-1.4046055566
H	5.0770382636	-0.2028034284	-2.4880074742
H	5.0770371743	-0.2028040041	2.4880095400

H	4.4118655242	-2.5411938636	-2.4879818800
H	4.4118647039	-2.5411945561	2.4879831224
H	2.7303060547	-4.2962646565	2.4880828713

Table S29. Cartesian coordinates (in Å) for the TAO-DFT S_0 state of [14]cyclacene.

H	-3.4934041313	4.2106916368	-2.4885833606
C	-3.5071384410	4.2271395294	-1.4051942931
C	-3.5071383098	4.2271393246	1.4051944780
C	-2.4807177877	4.9029195064	-0.7286236040
C	-2.4807177286	4.9029194104	0.7286237881
H	-3.4934039181	4.2106913047	2.4885835443
C	-1.3330605152	5.3419209522	-1.4050062864
C	-1.3330604142	5.3419208014	1.4050064277
H	-1.3282969535	5.3214443483	2.4884359176
H	-1.3282971373	5.3214445933	-2.4884357783
C	-0.1146435443	5.5004160976	-0.7285780339
C	-0.1146435055	5.5004160842	0.7285781009
C	2.2746118496	5.0030611086	0.7286170881
C	2.2746118225	5.0030611077	-0.7286171551
C	1.1093888619	5.3930959639	1.4049697701
C	1.1093887830	5.3930959265	-1.4049697739
H	1.1054613430	5.3723521297	-2.4883959246
H	1.1054614848	5.3723521579	2.4883959211
H	4.9632073741	-2.2707608451	-2.4881683250

C	4.9813947862	-2.2788677967	-1.4047310626
C	4.9813947211	-2.2788676720	1.4047310832
C	4.3480585054	-3.3319361584	-0.7284376272
C	4.3480584583	-3.3319360795	0.7284377160
H	4.9632072742	-2.2707606244	2.4881683463
C	3.5071386693	-4.2271385485	-1.4051942105
C	3.5071385405	-4.2271383385	1.4051943582
C	5.3538566230	-1.1079392853	0.7284665608
C	5.3538566141	-1.1079393261	-0.7284666267
C	5.4716879557	0.1151423898	1.4048334068
C	5.4716878596	0.1151423337	-1.4048335273
H	5.4513251144	0.1147826786	-2.4882669920
H	5.4513252526	0.1147827771	2.4882668726
C	5.3043444792	1.3324464898	0.7284657048
C	5.3043444484	1.3324464714	-0.7284658624
H	3.3151666555	4.3537870223	-2.4885144482
C	3.3285961224	4.3711946829	-1.4051489888
C	3.3285961448	4.3711946523	1.4051488837
C	4.2072258741	3.5129112516	-0.7284374808
C	4.2072258884	3.5129112494	0.7284373396
H	3.3151667124	4.3537869799	2.4885143453
C	4.8839062423	2.4870384567	-1.4047219575
C	4.8839062595	2.4870384588	1.4047218043
H	4.8658548301	2.4779977493	2.4881452435

H	4.8658547829	2.4779977420	-2.4881453961
H	-4.8658561062	-2.4779989034	2.4881456761
C	-4.8839075903	-2.4870396491	1.4047219333
C	-4.8839073906	-2.4870395483	-1.4047220957
H	-4.8658557851	-2.4779987352	-2.4881458376
C	-5.3043451753	-1.3324471364	-0.7284659720
C	-5.3043452648	-1.3324471817	0.7284658342
C	-5.4716880339	-0.1151426712	-1.4048336827
C	-5.4716881210	-0.1151427462	1.4048335982
H	-5.4513252128	-0.1147831473	2.4882674048
H	-5.4513250450	-0.1147830131	-2.4882674880
C	-5.3538560921	1.1079391758	-0.7284667279
C	-5.3538561327	1.1079391375	0.7284667157
C	-4.3480578906	3.3319364488	0.7284378433
C	-4.3480579299	3.3319365277	-0.7284377030
C	-4.9813939560	2.2788676909	1.4047312894
C	-4.9813939270	2.2788677790	-1.4047312225
H	-4.9632062492	2.2707606916	-2.4881687955
H	-4.9632062786	2.2707605271	2.4881688618
H	-3.3151670837	-4.3537879486	-2.4885147501
C	-3.3285965965	-4.3711957266	-1.4051490877
C	-3.3285966839	-4.3711957262	1.4051489893
C	-4.2072267278	-3.5129124027	-0.7284375779
C	-4.2072268104	-3.5129124404	0.7284374320

H	-3.3151672455	-4.3537879680	2.4885146547
C	-2.2746118542	-5.0030615382	0.7286171533
C	-2.2746118136	-5.0030615529	-0.7286171917
C	-1.1093885581	-5.3930957653	1.4049698105
C	-1.1093884766	-5.3930957956	-1.4049697792
H	-1.1054610917	-5.3723520055	-2.4883959962
H	-1.1054612245	-5.3723519308	2.4883960270
C	0.1146438817	-5.5004151717	0.7285781111
C	0.1146439239	-5.5004152378	-0.7285780156
C	2.4807179812	-4.9029182272	-0.7286235587
C	2.4807179238	-4.9029181243	0.7286237209
C	1.3330607957	-5.3419195853	-1.4050062477
C	1.3330606962	-5.3419193827	1.4050063971
H	1.3282972130	-5.3214429529	2.4884357992
H	1.3282973764	-5.3214432903	-2.4884356524
H	3.4934042006	-4.2106904039	2.4885832020
H	3.4934044090	-4.2106907638	-2.4885830575

7.2 KS-DFT Ground States

Table S30. Cartesian coordinates (in Å) for the KS-DFT S_0 state of [6]cyclacene.

H	2.1375411097	-1.0173392363	2.4837445481
C	2.1718292714	-1.0338482384	1.3993845276
C	2.1718291689	-1.0338481789	-1.3993845501
C	2.3818654878	0.1901686094	0.7242884151
C	2.3818654453	0.1901686048	-0.7242885197
H	2.1375410448	-1.0173391874	-2.4837443922
H	0.1875723048	-2.3669739468	2.4837550032
C	0.1902539902	-2.4020500424	1.3991724403
C	0.1902539841	-2.4020498950	-1.3991724836
C	1.3549339417	-1.9696475397	0.7243233086
C	1.3549339191	-1.9696474907	-0.7243234270
H	0.1875723053	-2.3669738299	-2.4837548675
C	1.9836841019	1.3668995427	1.3993338405
C	1.9836840097	1.3668994684	-1.3993338700
H	1.9553059735	1.3472216845	-2.4838973878
H	1.9553060094	1.3472217334	2.4838975365
C	-1.0282632540	-2.1606979867	0.7241410221
C	-1.0282632290	-2.1606979281	-0.7241411441
C	1.0282633572	2.1606982711	0.7241410995
C	1.0282633322	2.1606982125	-0.7241412214
H	-2.1375411683	1.0173388616	-2.4837443597
C	-2.1718292408	1.0338479497	-1.3993845384

C	-2.1718293433	1.0338480092	1.3993845158
C	-1.3549341488	1.9696474816	-0.7243234559
C	-1.3549341714	1.9696475305	0.7243233375
H	-2.1375412332	1.0173389105	2.4837445156
H	-1.9553057143	-1.3472216621	-2.4838971897
C	-1.9836837642	-1.3668994099	-1.3993338043
C	-1.9836838563	-1.3668994843	1.3993337748
C	-2.3818652314	-0.1901687532	-0.7242884695
C	-2.3818652739	-0.1901687578	0.7242883649
H	-1.9553057501	-1.3472217109	2.4838973384
C	-0.1902541493	2.4020500746	-1.3991725395
C	-0.1902541554	2.4020502220	1.3991724962
H	-0.1875725365	2.3669741143	2.4837551709
H	-0.1875725370	2.3669739976	-2.4837550352

Table S31. Cartesian coordinates (in Å) for the KS-DFT S_0 state of [7]cyclacene.

C	-0.3431160969	2.7560332273	-1.4134452267
C	-0.3431160683	2.7560331213	1.4134452841
C	0.8834033329	2.6222379862	-0.7343678553
C	0.8834032783	2.6222378075	0.7343680020
H	-0.3368161624	2.7039940748	2.4968398971
H	-2.3263804662	1.4316753326	-2.4967580291
C	-2.3698710285	1.4587099238	-1.4133235672
C	-2.3698709217	1.4587098481	1.4133236407

C	-1.5042347629	2.3387465922	-0.7344318002
C	-1.5042346487	2.3387464214	0.7344319797
H	-2.3263801302	1.4316751069	2.4967578359
C	1.9337057221	1.9749832392	-1.4133706897
C	1.9337056720	1.9749831879	1.4133707215
H	1.8937788801	1.9341565393	2.4964389579
H	1.8937790991	1.9341567613	-2.4964391916
C	-2.7556651289	0.2865321927	-0.7344087959
C	-2.7556649383	0.2865321658	0.7344089606
C	2.5999429000	0.9369707261	-0.7342787244
C	2.5999427241	0.9369706685	0.7342788721
C	-2.6016748074	-0.9372917119	1.4135285062
C	-2.6016748892	-0.9372917311	-1.4135284689
C	2.7588309630	-0.2863885372	1.4134082140
C	2.7588310711	-0.2863885639	-1.4134081578
C	-0.8839231769	-2.6239298094	-1.4133485808
C	-0.8839231596	-2.6239297245	1.4133486219
C	-1.9323878602	-1.9734473417	-0.7344705001
C	-1.9323877288	-1.9734472071	0.7344706388
H	-2.5497588232	-0.9187466807	2.4966581312
H	-2.5497591470	-0.9187467777	-2.4966583614
H	2.7060146873	-0.2809663517	2.4967639172
C	2.3673384593	-1.4565173790	-0.7344173458
C	2.3673382864	-1.4565172685	0.7344175259

C	1.5064983054	-2.3411808009	-1.4133929366
C	1.5064982313	-2.3411806991	1.4133930113
H	-0.8665980150	-2.5722426947	2.4965271413
H	-0.8665981038	-2.5722430232	-2.4965273673
H	-0.3368162384	2.7039944506	-2.4968401064
C	0.3430763719	-2.7525457653	-0.7344373761
C	0.3430763418	-2.7525455742	0.7344375436
H	2.7060150653	-0.2809664196	-2.4967641288
H	1.4785136700	-2.2981143201	2.4968207231
H	1.4785138957	-2.2981146453	-2.4968209156

Table S32. Cartesian coordinates (in Å) for the KS-DFT S_0 state of [8]cyclacene.

H	-2.8793767224	1.2485352740	2.4881956372
C	-2.9064161371	1.2600794106	1.4035172857
C	-2.9064154106	1.2600790963	-1.4035173885
C	-3.1551511385	0.0516858912	0.7281133016
C	-3.1551508536	0.0516858870	-0.7281135399
H	-2.8793759764	1.2485349509	-2.4881952954
C	-2.9464701776	-1.1641904601	1.4034711164
C	-2.9464694376	-1.1641901681	-1.4034712203
H	-2.9191325436	-1.1533361840	-2.4881377420
H	-2.9191333021	-1.1533364834	2.4881380827
C	-2.2675699127	-2.1944318761	0.7281372448
C	-2.2675697077	-2.1944316781	-0.7281374825

H	1.1533367524	-2.9191322527	-2.4881383281
C	1.1641909009	-2.9464695501	-1.4034714411
C	1.1641911930	-2.9464702900	1.4034713372
C	-0.0516858488	-3.1551511669	-0.7281137707
C	-0.0516858530	-3.1551514516	0.7281135324
H	1.1533370519	-2.9191330113	2.4881386689
H	2.8793762194	-1.2485347989	-2.4881957563
C	2.9064159818	-1.2600790060	-1.4035175585
C	2.9064167082	-1.2600793204	1.4035174557
C	2.1944323379	-2.2675692302	-0.7281375402
C	2.1944325360	-2.2675694351	0.7281373025
H	2.8793769654	-1.2485351221	2.4881960981
C	-1.2600797703	-2.9064156372	-1.4035176351
C	-1.2600800846	-2.9064163635	1.4035175323
H	-1.2485358123	-2.8793764837	2.4881962938
H	-1.2485354892	-2.8793757380	-2.4881959519
C	-2.1944320457	2.2675691337	-0.7281374049
C	-2.1944322437	2.2675693386	0.7281371672
C	-1.1641906646	2.9464689246	-1.4034712054
C	-1.1641909567	2.9464696645	1.4034711015
H	-1.1533369776	2.9191328041	2.4881380308
H	-1.1533366781	2.9191320456	-2.4881376900
C	0.0516856851	3.1551509021	-0.7281136378
C	0.0516856894	3.1551511868	0.7281133996

H	2.9191331790	1.1533368036	2.4881380982
C	2.9464700245	1.1641908810	1.4034711224
C	2.9464692846	1.1641905889	-1.4034712263
C	2.2675695781	2.1944320632	0.7281371920
C	2.2675693732	2.1944318651	-0.7281374297
H	2.9191324205	1.1533365040	-2.4881377575
C	3.1551512674	-0.0516855349	0.7281133607
C	3.1551509827	-0.0516855306	-0.7281135989
C	1.2600796026	2.9064160699	1.4035173690
C	1.2600792882	2.9064153434	-1.4035174717
H	1.2485351995	2.8793756990	-2.4881955107
H	1.2485355227	2.8793764449	2.4881958527

Table S33. Cartesian coordinates (in Å) for the KS-DFT S_0 state of [9]cyclacene.

H	-3.5305223245	-0.0168403971	2.4964307487
C	-3.5718703776	-0.0169908456	1.4124290929
C	-3.5718704158	-0.0169908457	-1.4124291128
C	-3.3462903945	-1.2270544356	0.7336619992
C	-3.3462904669	-1.2270544659	-0.7336619782
H	-3.5305224566	-0.0168403966	-2.4964308345
C	-2.7165979381	-2.2847048944	1.4125179046
C	-2.7165979832	-2.2847049348	-1.4125179073
H	-2.6848502152	-2.2573575333	-2.4963721971
H	-2.6848500876	-2.2573574274	2.4963721273
C	-1.7598005814	-3.0577775559	0.7334478310

C	-1.7598006186	-3.0577776286	-0.7334477905
H	1.7653596611	-3.0028506305	-2.4963538823
C	1.7882483290	-3.0424835969	-1.4126489755
C	1.7882483174	-3.0424835822	1.4126489915
C	0.6308609826	-3.4596148959	-0.7335926572
C	0.6308609693	-3.4596148211	0.7335927008
H	1.7653595977	-3.0028505086	2.4963538432
H	3.3110371758	1.2138811649	-2.4963799394
C	3.3485073985	1.2278846334	-1.4124133971
C	3.3485074235	1.2278846444	1.4124134090
H	3.3110370885	1.2138811301	2.4963799106
C	-0.5994675272	-3.4665034390	-1.4122958784
C	-0.5994675195	-3.4665033825	1.4122958964
H	-0.5925158966	-3.4230802437	2.4960608645
H	-0.5925159324	-3.4230804152	-2.4960609095
C	-3.3564363251	1.1948954040	-0.7336628279
C	-3.3564362564	1.1948953758	0.7336628497
C	-2.7360281896	2.2580082044	-1.4125126510
C	-2.7360281594	2.2580081767	1.4125126536
H	-2.7038358147	2.2307772402	2.4963564379
H	-2.7038359265	2.2307773336	-2.4963564935
C	-1.7865537834	3.0400170175	-0.7334445090
C	-1.7865537505	3.0400169538	0.7334445526
H	1.7391777651	3.0207114730	2.4963674623

C	1.7615122852	3.0602465845	1.4126450349
C	1.7615122833	3.0602465764	-1.4126450116
C	0.5999706564	3.4657298306	0.7335973001
C	0.5999706667	3.4657298933	-0.7335972528
H	1.7391778128	3.0207115729	-2.4963674819
C	2.7157447272	2.2837611357	0.7336631716
C	2.7157447681	2.2837611735	-0.7336631368
C	-0.6303453664	3.4601742807	1.4123039922
C	-0.6303453697	3.4601743088	-1.4123039654
H	-0.6229845073	3.4167798562	-2.4960627982
H	-0.6229844750	3.4167797134	2.4960627758
H	3.3208793634	-1.1819981195	2.4963662003
C	3.3585258692	-1.1956669225	1.4124046862
C	3.3585258548	-1.1956669150	-1.4124046768
C	3.5679496975	0.0169864325	0.7336102815
C	2.7351212990	-2.2570819055	0.7336589176
C	2.7351213458	-2.2570819474	-0.7336588845
C	3.5679497502	0.0169864327	-0.7336102508
H	3.3208794629	-1.1819981553	-2.4963662363

Table S34. Cartesian coordinates (in Å) for the KS-DFT S_0 state of [10]cyclacene.

H	-3.8537281830	0.6386820569	2.4900424816
C	-3.8808273995	0.6426864025	1.4053401757
C	-3.8808275866	0.6426864609	-1.4053401197

C	-3.8843486052	-0.5878633757	0.7300022390
C	-3.8843487387	-0.5878633843	-0.7300021406
H	-3.8537284956	0.6386821635	-2.4900426201
C	-3.5250314928	-1.7648262571	1.4053167203
C	-3.5250316079	-1.7648262943	-1.4053167173
H	-3.5015171065	-1.7529829564	-2.4900944428
H	-3.5015169104	-1.7529829046	2.4900942598
C	-2.8040224215	-2.7623885901	0.7300708187
C	-2.8040224998	-2.7623886668	-0.7300707627
H	0.5809088254	-3.8643635840	-2.4900460791
C	0.5845064399	-3.8912236204	-1.4053248590
C	0.5845063836	-3.8912234353	1.4053249130
C	-0.6459670924	-3.8753849914	-0.7299900392
C	-0.6459670823	-3.8753848604	0.7299901347
H	0.5809087224	-3.8643632755	2.4900459394
H	3.5015169774	1.7529830189	-2.4900943525
C	3.5250314837	1.7648263343	-1.4053166838
C	3.5250313686	1.7648262970	1.4053166868
H	3.5015167813	1.7529829670	2.4900941695
C	-1.8171619246	-3.4978374560	-1.4053688266
C	-1.8171618866	-3.4978373456	1.4053688272
H	-1.8047072908	-3.4738857393	2.4901172264
H	-1.8047073443	-3.4738859273	-2.4901174114
C	-3.4838494187	1.8073314565	-0.7299986411

C	-3.4838492699	1.8073313702	0.7299987950
C	-2.7616037504	2.8036796575	-1.4053727338
C	-2.7616035812	2.8036794858	1.4053728210
H	-2.7450206710	2.7868669790	2.4901974536
H	-2.7450209588	2.7868672701	-2.4901975647
C	-1.7550642943	3.5118391513	-0.7300235001
C	-1.7550642103	3.5118390014	0.7300236529
H	1.8047071833	3.4738856173	2.4901170902
C	1.8171618051	3.4978372363	1.4053687756
C	1.8171618431	3.4978373467	-1.4053687749
C	0.6459670726	3.8753848139	0.7299900952
C	0.6459670827	3.8753849448	-0.7299899997
H	1.8047072369	3.4738858052	-2.4901172753
C	2.8040223307	2.7623886087	0.7300707797
C	2.8040224091	2.7623886854	-0.7300707236
C	-0.5845063422	3.8912233162	1.4053248632
C	-0.5845063984	3.8912235012	-1.4053248093
H	-0.5809087731	3.8643634287	-2.4900459493
H	-0.5809086703	3.8643631205	2.4900458096
H	2.7450208168	-2.7868669932	2.4901975476
C	2.7616036986	-2.8036795031	1.4053728572
C	2.7616038677	-2.8036796748	-1.4053727701
C	1.7550642808	-3.5118390104	0.7300236885
C	1.7550643647	-3.5118391602	-0.7300235357

H	2.7450211045	-2.7868672843	-2.4901976586
H	3.8537282243	-0.6386819248	2.4900424906
C	3.8808274326	-0.6426862915	1.4053401790
C	3.8808276198	-0.6426863499	-1.4053401231
C	3.8843485674	0.5878635086	0.7300022256
C	3.4838493488	-1.8073312746	0.7299988163
C	3.4838494976	-1.8073313608	-0.7299986624
C	3.8843487009	0.5878635172	-0.7300021271
H	3.8537285369	-0.6386820312	-2.4900426291

Table S35. Cartesian coordinates (in Å) for the KS-DFT S_0 state of [11]cyclacene.

H	-3.9592132302	-1.6612054382	2.4960922767
C	-3.9897366199	-1.6741298661	1.4118129880
C	-3.9809366652	-1.7032298194	-1.4109130119
C	-3.3404618860	-2.7367473667	0.7332445139
C	-3.3424619511	-2.7349473983	-0.7332445594
H	-3.9518133030	-1.6903053998	-2.4952923091
C	-2.4429429188	-3.5513077098	1.4118696334
C	-2.4196430714	-3.5702078795	-1.4116697179
H	-2.3981597830	-3.5384602055	-2.4956544046
H	-2.4204595781	-3.5185599400	2.4958541991
C	-1.3241318942	-4.0981026368	0.7333153995
C	-1.3277319509	-4.0984028049	-0.7333154384
C	1.1043201307	-4.1617047358	-0.7331147728

C	1.1080200633	-4.1612045093	0.7332147596
C	-0.0999622346	-4.3077126251	-1.4115508707
C	-0.1289622252	-4.3026122319	1.4118507739
H	-0.1281703153	-4.2682187588	2.4961365817
H	-0.0991703153	-4.2748193374	-2.4958368713
C	-4.2998065621	-0.4993144928	-0.7330789798
C	-4.2999065988	-0.5016145226	0.7334789601
C	-4.2703671413	0.7125873502	-1.4123610041
C	-4.2670673058	0.7437873161	1.4110610678
H	-4.2310581396	0.7385333532	2.4952389866
H	-4.2337579474	0.7063333918	-2.4964388158
C	-3.8884742523	1.8981641582	-0.7346339709
C	-3.8887743761	1.8958641986	0.7322339869
H	-1.0823461341	4.1278591904	2.4964927133
C	-1.0910547010	4.1631833656	1.4123706355
C	-1.1203546303	4.1580830249	-1.4111705065
C	-2.2394033842	3.6830347854	0.7335301685
C	-2.2366032892	3.6832346295	-0.7330301431
H	-1.1113460521	4.1237587488	-2.4952923652
C	-3.1850348314	2.9230641743	1.4109718304
C	-3.2033345986	2.8998639979	-1.4123717022
H	-3.1782378974	2.8779029004	-2.4966070018
H	-3.1619381788	2.9006031231	2.4953073216
H	3.3240242356	2.7015028374	2.4967889434

C	3.3517791751	2.7246325620	1.4126624205
C	3.3337790328	2.7491325003	-1.4126624093
C	2.4298661878	3.5608567105	0.7333051078
C	2.4320660997	3.5588566098	-0.7330051461
H	3.3067240762	2.7269027524	-2.4967888474
C	1.3409258619	4.0934260443	1.4125211505
C	1.3128257242	4.1049257632	-1.4115210777
H	1.3028601178	4.0738282311	-2.4957676269
H	1.3309602958	4.0612285925	2.4967678788
C	0.1134631251	4.3021098782	0.7339999441
C	0.1164631055	4.3019096876	-0.7327999478
C	3.9831108143	1.6866493845	-0.7335854050
C	3.9828108332	1.6890493808	0.7332853651
C	4.3001395761	0.5156094387	-1.4121518905
C	4.3023394813	0.4853093960	1.4119518631
H	4.2675781447	0.4815752453	2.4961217478
H	4.2651783208	0.5117752759	-2.4963218112
C	4.2664424003	-0.7292303266	-0.7332973661
C	4.2660422794	-0.7265303126	0.7328973520
H	2.2074942251	-3.6610137037	2.4962894370
C	2.2270433175	-3.6937647364	1.4122813117
C	2.2509435578	-3.6772650979	-1.4123813882
C	3.1936979351	-2.9095321424	0.7331880967
C	3.1911981020	-2.9111322843	-0.7332880968

H	2.2309945762	-3.6443142468	-2.4963897153
C	3.8840735421	-1.9118122428	1.4118766371
C	3.8941738479	-1.8833123647	-1.4121766866
H	3.8621357391	-1.8678862285	-2.4962555434
H	3.8532352889	-1.8961860189	2.4960553515

Table S36. Cartesian coordinates (in Å) for the KS-DFT S_0 state of [12]cyclacene.

H	-0.4010498537	4.6722585530	-2.4911996377
C	-0.4026143015	4.6946615567	-1.4063226700
C	-0.4026142774	4.6946612746	1.4063226828
C	0.8247826720	4.6312900503	-0.7309909279
C	0.8247826475	4.6312899036	0.7309909316
H	-0.4010498031	4.6722579389	2.4911996304
C	1.9986128795	4.2670433423	-1.4062210450
C	1.9986127629	4.2670430861	1.4062210556
H	1.9894626459	4.2474823904	2.4911002689
H	1.9894628961	4.2474829410	-2.4911002792
C	3.0303085377	3.5985604876	-0.7310300540
C	3.0303084425	3.5985603719	0.7310300544
C	4.4244654011	1.6017694859	0.7311532250
C	4.4244655442	1.6017695373	-0.7311532235
C	3.8627993706	2.6949588843	1.4066384590
C	3.8627996021	2.6949590459	-1.4066384503
H	3.8416275540	2.6800873250	-2.4913626047

H	3.8416270549	2.6800869765	2.4913625896
C	-1.6017697777	4.4244659298	0.7311533004
C	-1.6017698287	4.4244660734	-0.7311532978
C	-2.6949591305	3.8627996008	1.4066385106
C	-2.6949592909	3.8627998328	-1.4066385020
H	-2.6800873776	3.8416277479	-2.4913627330
H	-2.6800870315	3.8416272476	2.4913627178
C	-3.5985603314	3.0303083672	0.7310300449
C	-3.5985604463	3.0303084626	-0.7310300456
H	-4.6722580367	-0.4010499244	-2.4911992232
C	-4.6946606625	-0.4026143573	-1.4063225147
C	-4.6946603816	-0.4026143327	1.4063225259
C	-4.6312894083	0.8247824750	-0.7309908400
C	-4.6312892624	0.8247824506	0.7309908416
H	-4.6722574246	-0.4010498728	2.4911992141
C	-4.2670429453	1.9986126210	-1.4062209675
C	-4.2670426908	1.9986125045	1.4062209762
H	-4.2474821192	1.9894625983	2.4911000600
H	-4.2474826671	1.9894628484	-2.4911000721
H	0.4010497910	-4.6722583926	-2.4911992893
C	0.4026142129	-4.6946611907	-1.4063225459
C	0.4026141886	-4.6946609085	1.4063225587
C	-0.8247826034	-4.6312897769	-0.7309908511
C	-0.8247825789	-4.6312896301	0.7309908546

H	0.4010497401	-4.6722577784	2.4911992819
C	-1.9986126387	-4.2670430576	-1.4062209249
C	-1.9986125221	-4.2670428015	1.4062209353
H	-1.9894625035	-4.2474822844	2.4910999316
H	-1.9894627537	-4.2474828349	-2.4910999422
C	-3.0303082571	-3.5985604031	-0.7310299893
C	-3.0303081618	-3.5985602874	0.7310299896
C	-3.8627990351	-2.6949589249	1.4066383739
C	-3.8627992667	-2.6949590864	-1.4066383653
H	-3.8416273522	-2.6800874228	-2.4913623706
C	1.6017695725	-4.4244657263	0.7311532308
C	1.6017696236	-4.4244658702	-0.7311532282
C	2.6949588087	-3.8627994754	1.4066384128
C	2.6949589694	-3.8627997079	-1.4066384042
H	2.6800871689	-3.8416277412	-2.4913624635
H	2.6800868222	-3.8416272400	2.4913624483
C	3.5985600635	-3.0303084440	0.7310300015
C	3.5985601786	-3.0303085395	-0.7310300022
H	4.6722580926	0.4010496748	-2.4911992887
C	4.6946607559	0.4026140729	-1.4063225373
C	4.6946604753	0.4026140483	1.4063225483
C	4.6312893322	-0.8247827476	-0.7309908337
C	4.6312891864	-0.8247827232	0.7309908352
H	4.6722574808	0.4010496232	2.4911992795

C	4.2670427123	-1.9986128201	-1.4062209257
C	4.2670424576	-1.9986127035	1.4062209344
H	4.2474819605	-1.9894627916	2.4910999421
H	4.2474825086	-1.9894630420	-2.4910999544
C	-4.4244651977	-1.6017696822	0.7311531917
C	-4.4244653410	-1.6017697336	-0.7311531901
H	-3.8416268527	-2.6800870745	2.4913623554

Table S37. Cartesian coordinates (in Å) for the KS-DFT S_0 state of [13]cyclacene.

H	-4.9033323164	1.3901063064	2.4907396310
C	-4.9229326794	1.3955102669	1.4058240001
C	-4.9083826007	1.4456271964	-1.4057850288
C	-5.1002091607	0.2043999262	0.7300411717
C	-5.1000214662	0.2036152739	-0.7300522707
H	-4.8884669538	1.4403776663	-2.4906809360
C	-4.9959182398	-1.0479566241	1.4058003859
C	-5.0058193717	-0.9968469245	-1.4058313906
H	-4.9845743983	-0.9928291708	-2.4907085193
H	-4.9750559412	-1.0440683544	2.4906782277
C	-4.5995891884	-2.1851084647	0.7301457334
C	-4.5988846678	-2.1857654394	-0.7300018692
C	-3.0472587492	-4.0703336127	-0.7296509370
C	-3.0479329164	-4.0697068818	0.7301386771
C	-3.9585125070	-3.2058448718	-1.4057885582

C	-3.9259335583	-3.2462232838	1.4061634466
H	-3.9099003954	-3.2329520991	2.4910407213
H	-3.9419025539	-3.1920748090	-2.4906462929
C	3.6962290365	-3.5386479028	0.7296238724
C	3.6964003850	-3.5382491308	-0.7302197625
C	4.4512374449	-2.5336830922	1.4053316929
C	4.4243649160	-2.5788578398	-1.4061011170
C	2.7245490475	-4.3352005057	-1.4059188487
C	2.7690968896	-4.3067806353	1.4057726239
H	2.7123362091	-4.3160347865	-2.4907116657
C	1.6318295858	-4.8407100431	-0.7298652883
C	1.6312542701	-4.8405231671	0.7300689662
H	-1.9547960810	-4.6849602120	2.4908601668
C	-1.9619653476	-4.7022698477	1.4059193985
C	-2.0099604505	-4.6822273909	-1.4052353385
C	-0.8027821973	-5.0285457591	0.7303047817
C	-0.8021074199	-5.0290769081	-0.7295346532
H	-2.0027867413	-4.6650568164	-2.4901772334
C	0.4526345171	-5.0873042464	1.4059635291
C	0.4003573760	-5.0927112693	-1.4053510695
H	0.3989787404	-5.0734301979	-2.4902900860
H	0.4515283860	-5.0667339934	2.4908793559
H	0.8275886631	5.0133522118	-2.4905619051
C	0.8303048525	5.0340655204	-1.4056680365

C	0.7784769030	5.0418444779	1.4058263968
H	0.7753505852	5.0208767961	2.4907169484
C	-0.4249169758	5.0829019461	0.7299386421
C	-0.4255883368	5.0830083834	-0.7301015877
C	-1.6576048609	4.8380560535	1.4055187343
C	-1.6079397495	4.8547595567	-1.4059566469
H	-1.6018858698	4.8364121814	-2.4908993572
H	-1.6519456045	4.8193811082	2.4904429407
C	-2.7411630642	4.3130358861	0.7296257702
C	-2.7418756435	4.3127727090	-0.7302698308
C	-4.4309221377	2.5510529239	-0.7299875821
C	-4.4307401614	2.5518633191	0.7300301634
C	-3.6820604466	3.5603028327	-1.4061202758
C	-3.7179862084	3.5228008774	1.4057843379
H	-3.7040821468	3.5094017377	2.4907102919
H	-3.6682169535	3.5470654557	-2.4910486557
H	3.0175547324	4.0746639012	2.4909698375
C	3.0313755317	4.0928267052	1.4061594837
C	3.0735319875	4.0621059658	-1.4057653287
C	1.9842816329	4.6875670327	0.7302175347
C	1.9837955827	4.6883283210	-0.7298932640
H	3.0602576857	4.0446131239	-2.4906074353
C	3.9360124632	3.2224550635	-0.7296577166
C	3.9364903389	3.2217020259	0.7301478122

C	4.6152148878	2.1649680003	-1.4055106336
C	4.5927597296	2.2116677850	1.4056136949
H	4.5752421054	2.2031081852	2.4905344788
H	4.5977729037	2.1570157595	-2.4904224340
C	4.9911729134	1.0208834899	-0.7300460966
C	4.9916159331	1.0200511785	0.7297179381
C	4.9082396322	-1.4196687874	0.7295540858
C	4.9078899547	-1.4188716076	-0.7302783286
C	5.1071607070	-0.1786758610	1.4053288239
C	5.1041243730	-0.2306594291	-1.4059712573
H	5.0840549636	-0.2300238150	-2.4908965489
H	5.0882287712	-0.1782004691	2.4902758586
H	4.4052800123	-2.5676604607	-2.4909543112
H	4.4325455200	-2.5231507586	2.4901612368
H	2.7568424961	-4.2876121174	2.4906016497

Table S38. Cartesian coordinates (in Å) for the KS-DFT S_0 state of [14]cyclacene.

H	-3.4926650903	4.2084717155	-2.4922529320
C	-3.5063391160	4.2246985175	-1.4074052498
C	-3.5063396073	4.2246991133	1.4074051803
C	-2.4805021953	4.9003264853	-0.7318063263
C	-2.4805023476	4.9003267895	0.7318061753
H	-3.4926657804	4.2084725567	2.4922531245
C	-1.3328599570	5.3376503319	-1.4072103029

C	-1.3328601457	5.3376510856	1.4072102254
H	-1.3280668770	5.3171521372	2.4920873191
H	-1.3280666102	5.3171510738	-2.4920871347
C	-0.1151075126	5.4966953660	-0.7317531833
C	-0.1151075207	5.4966957061	0.7317530302
C	2.2731613824	4.9989902811	0.7317950502
C	2.2731612446	4.9989899724	-0.7317951946
C	1.1081693216	5.3877726908	1.4071780407
C	1.1081691686	5.3877719327	-1.4071781142
H	1.1042151450	5.3668617788	-2.4920481051
H	1.1042153594	5.3668628483	2.4920482935
H	4.9628660578	-2.2702406462	-2.4918149596
C	4.9805118419	-2.2780420508	-1.4069187457
C	4.9805125324	-2.2780423712	1.4069186904
C	4.3476894432	-3.3307485462	-0.7316109129
C	4.3476897080	-3.3307487525	0.7316107740
H	4.9628670325	-2.2702411038	2.4918151659
C	3.5063390133	-4.2246980632	-1.4074050485
C	3.5063395046	-4.2246986591	1.4074049790
C	5.3534503623	-1.1078874629	0.7316275625
C	5.3534500367	-1.1078873939	-0.7316276870
C	5.4708153493	0.1146566368	1.4070172143
C	5.4708145934	0.1146566227	-1.4070172602
H	5.4511692640	0.1143308151	-2.4919156231

H	5.4511703317	0.1143308345	2.4919158389
C	5.3036369259	1.3314185526	0.7316284484
C	5.3036366038	1.3314184715	-0.7316285702
H	3.3127597024	4.3495155353	-2.4921658663
C	3.3263697733	4.3669787993	-1.4073498787
C	3.3263702351	4.3669794109	1.4073498171
C	4.2057964482	3.5103929747	-0.7316107096
C	4.2057967036	3.5103931906	0.7316105776
H	3.3127603495	4.3495163975	2.4921660669
C	4.8821461521	2.4849820777	-1.4069121937
C	4.8821468277	2.4849824238	1.4069121433
H	4.8643202772	2.4761235240	2.4917882734
H	4.8643193255	2.4761230327	-2.4917880621
H	-4.8643201384	-2.4761235991	2.4917889967
C	-4.8821471828	-2.4849828635	1.4069123934
C	-4.8821465072	-2.4849825174	-1.4069124438
H	-4.8643191868	-2.4761231078	-2.4917887854
C	-5.3036369392	-1.3314187305	-0.7316287152
C	-5.3036372612	-1.3314188116	0.7316285934
C	-5.4708151514	-0.1146567473	-1.4070175485
C	-5.4708159073	-0.1146567615	1.4070175025
H	-5.4511701928	-0.1143309200	2.4919166863
H	-5.4511691252	-0.1143309007	-2.4919164705
C	-5.3534504040	1.1078874300	-0.7316278393

C	-5.3534507297	1.1078874990	0.7316277148
C	-4.3476898847	3.3307490380	0.7316109043
C	-4.3476896199	3.3307488317	-0.7316110432
C	-4.9805129858	2.2780426339	1.4069189602
C	-4.9805122954	2.2780423135	-1.4069190155
H	-4.9628659189	2.2702405760	-2.4918157615
H	-4.9628668936	2.2702410336	2.4918159678
H	-3.3127595176	-4.3495155383	-2.4921663212
C	-3.3263696816	-4.3669791837	-1.4073500399
C	-3.3263701434	-4.3669797952	1.4073499783
C	-4.2057965116	-3.5103933522	-0.7316108169
C	-4.2057967670	-3.5103935682	0.7316106850
H	-3.3127601647	-4.3495164004	2.4921665218
C	-2.2731611209	-4.9989904337	0.7317950972
C	-2.2731609832	-4.9989901251	-0.7317952416
C	-1.1081689694	-5.3877727089	1.4071780784
C	-1.1081688165	-5.3877719508	-1.4071781519
H	-1.1042149667	-5.3668617089	-2.4920482018
H	-1.1042151811	-5.3668627783	2.4920483902
C	0.1151078344	-5.4966955501	0.7317530098
C	0.1151078263	-5.4966952100	-0.7317531630
C	2.4805023074	-4.9003261488	-0.7318062419
C	2.4805024598	-4.9003264531	0.7318060909
C	1.3328602087	-5.3376499974	-1.4072102123

C	1.3328603973	-5.3376507511	1.4072101348
H	1.3280670167	-5.3171520791	2.4920870390
H	1.3280667499	-5.3171510157	-2.4920868546
H	3.4926658903	-4.2084725607	2.4922525241
H	3.4926652003	-4.2084717194	-2.4922523316

7.3 TAO-DFT Triplet States

Table S39. Cartesian coordinates (in Å) for the TAO-DFT T_1 state of [6]cyclacene.

H	2.1312649041	-1.0145040733	2.4864870400
C	2.1680577953	-1.0321972691	1.4040142759
C	2.1680577952	-1.0321972691	-1.4040142760
C	2.3857818093	0.1903526902	0.7233239491
C	2.3857818093	0.1903526902	-0.7233239491
H	2.1312649041	-1.0145040733	-2.4864870400
H	0.1870649367	-2.3599587921	2.4865613205
C	0.1900250264	-2.3981016754	1.4038828312
C	0.1900250263	-2.3981016755	-1.4038828312
C	1.3574035750	-1.9732663516	0.7233543615
C	1.3574035750	-1.9732663517	-0.7233543616
H	0.1870649367	-2.3599587922	-2.4865613205
C	1.9799256338	1.3643926302	1.4039945102
C	1.9799256338	1.3643926303	-1.4039945102
H	1.9490434459	1.3430055269	-2.4866738346
H	1.9490434458	1.3430055268	2.4866738346

C	-1.0299183283	-2.1644889489	0.7231486171
C	-1.0299183284	-2.1644889490	-0.7231486170
C	1.0299181705	2.1644885344	0.7231484302
C	1.0299181706	2.1644885344	-0.7231484302
H	-2.1312647293	1.0145045083	-2.4864870845
C	-2.1680575945	1.0321978171	-1.4040142908
C	-2.1680575945	1.0321978172	1.4040142907
C	-1.3574031052	1.9732664448	-0.7233542807
C	-1.3574031053	1.9732664449	0.7233542807
H	-2.1312647295	1.0145045083	2.4864870845
H	-1.9490438596	-1.3430055597	-2.4866743712
C	-1.9799262919	-1.3643927751	-1.4039946919
C	-1.9799262919	-1.3643927751	1.4039946920
C	-2.3857820856	-0.1903523054	-0.7233240537
C	-2.3857820856	-0.1903523054	0.7233240537
H	-1.9490438596	-1.3430055597	2.4866743713
C	-0.1900246545	2.3981011247	-1.4038826675
C	-0.1900246545	2.3981011246	1.4038826675
H	-0.1870646479	2.3599584742	2.4865608318
H	-0.1870646479	2.3599584743	-2.4865608318

Table S40. Cartesian coordinates (in Å) for the TAO-DFT T_1 state of [7]cyclacene.

C	-0.3432956550	2.7672237968	-1.4050717739
C	-0.3432956550	2.7672237968	1.4050717739

C	0.8864199251	2.6275664590	-0.7254308700
C	0.8864199251	2.6275664590	0.7254308700
H	-0.3383523001	2.7263700544	2.4877076123
H	-2.3451189255	1.4426152187	-2.4876408116
C	-2.3789894966	1.4635817373	-1.4049740924
C	-2.3789894966	1.4635817373	1.4049740924
C	-1.5050841866	2.3409327469	-0.7254654659
C	-1.5050841866	2.3409327469	0.7254654660
H	-2.3451189255	1.4426152187	2.4876408116
C	1.9447334813	1.9860015828	-1.4050277365
C	1.9447334813	1.9860015828	1.4050277365
H	1.9141029723	1.9547341668	2.4874573198
H	1.9141029722	1.9547341668	-2.4874573198
C	-2.7598576749	0.2857724643	-0.7255279455
C	-2.7598576749	0.2857724643	0.7255279455
C	2.6057526737	0.9400651448	-0.7253915942
C	2.6057526738	0.9400651448	0.7253915942
C	-2.6149950771	-0.9429886111	1.4051432941
C	-2.6149950771	-0.9429886111	-1.4051432941
C	2.7708628329	-0.2863448369	1.4050565427
C	2.7708628329	-0.2863448370	-1.4050565427
C	-0.8894204642	-2.6368277077	-1.4049773840
C	-0.8894204642	-2.6368277077	1.4049773840
C	-1.9376407764	-1.9785444367	-0.7255333589

C	-1.9376407764	-1.9785444367	0.7255333589
H	-2.5755683058	-0.9288961602	2.4876406562
H	-2.5755683057	-0.9288961601	-2.4876406562
H	2.7296573310	-0.2821403804	2.4876754334
C	2.3699945344	-1.4571883822	-0.7254425891
C	2.3699945344	-1.4571883822	0.7254425892
C	1.5115811640	-2.3496528238	-1.4050336147
C	1.5115811640	-2.3496528237	1.4050336148
H	-0.8762762893	-2.5976435584	2.4875119018
H	-0.8762762892	-2.5976435584	-2.4875119019
H	-0.3383523001	2.7263700543	-2.4877076123
C	0.3423507138	-2.7559257681	-0.7255521357
C	0.3423507138	-2.7559257681	0.7255521357
H	2.7296573310	-0.2821403804	-2.4876754334
H	1.4898208502	-2.3160425330	2.4876921862
H	1.4898208502	-2.3160425330	-2.4876921862

Table S41. Cartesian coordinates (in Å) for the TAO-DFT T_1 state of [8]cyclacene.

H	-2.8725386615	1.2455816392	2.4879725249
C	-2.9055940020	1.2597620903	1.4050940672
C	-2.9055940010	1.2597620907	-1.4050940673
C	-3.1574701404	0.0517357618	0.7265179699
C	-3.1574701395	0.0517357620	-0.7265179709
H	-2.8725386603	1.2455816401	-2.4879725243

C	-2.9457326638	-1.1639029507	1.4050441942
C	-2.9457326616	-1.1639029497	-1.4050441952
H	-2.9123844432	-1.1507533853	-2.4879182058
H	-2.9123844461	-1.1507533866	2.4879182056
C	-2.2693809962	-2.1961565908	0.7265395521
C	-2.2693809953	-2.1961565902	-0.7265395532
H	1.1507532277	-2.9123843470	-2.4879180180
C	1.1639027371	-2.9457324806	-1.4050441202
C	1.1639027369	-2.9457324803	1.4050441209
C	-0.0517357853	-3.1574699429	-0.7265178925
C	-0.0517357855	-3.1574699426	0.7265178926
H	1.1507532272	-2.9123843459	2.4879180194
H	2.8725384982	-1.2455816914	-2.4879723405
C	2.9055937673	-1.2597621274	-1.4050939929
C	2.9055937686	-1.2597621272	1.4050939928
C	2.1961564171	-2.2693810587	-0.7265395235
C	2.1961564171	-2.2693810583	0.7265395238
H	2.8725384997	-1.2455816906	2.4879723411
C	-1.2597619246	-2.9055938049	-1.4050939859
C	-1.2597619258	-2.9055938058	1.4050939857
H	-1.2455815281	-2.8725385480	2.4879723213
H	-1.2455815264	-2.8725385473	-2.4879723208
C	-2.1961565575	2.2693811347	-0.7265395876
C	-2.1961565574	2.2693811342	0.7265395878

C	-1.1639028060	2.9457327257	-1.4050442131
C	-1.1639028058	2.9457327255	1.4050442138
H	-1.1507532659	2.9123845065	2.4879182496
H	-1.1507532664	2.9123845075	-2.4879182483
C	0.0517358781	3.1574700766	-0.7265179497
C	0.0517358783	3.1574700762	0.7265179498
H	2.9123844936	1.1507532101	2.4879181764
C	2.9457327102	1.1639027477	1.4050441825
C	2.9457327080	1.1639027468	-1.4050441834
C	2.2693811490	2.1961564917	0.7265395696
C	2.2693811481	2.1961564912	-0.7265395707
H	2.9123844906	1.1507532088	-2.4879181766
C	3.1574700618	-0.0517359198	0.7265179372
C	3.1574700607	-0.0517359200	-0.7265179382
C	1.2597621370	2.9055938818	1.4050940432
C	1.2597621360	2.9055938812	-1.4050940434
H	1.2455816980	2.8725385807	-2.4879724631
H	1.2455816995	2.8725385811	2.4879724636

Table S42. Cartesian coordinates (in Å) for the TAO-DFT T_1 state of [9]cyclacene.

H	-3.5426245489	-0.0166319796	2.4877242856
C	-3.5749952595	-0.0167447931	1.4046092542
C	-3.5749952594	-0.0167447928	-1.4046092545
C	-3.3435280435	-1.2281208038	0.7265857154

C	-3.3435280431	-1.2281208036	-0.7265857159
H	-3.5426245487	-0.0166319789	-2.4877242858
C	-2.7209742877	-2.2927357578	1.4047656263
C	-2.7209742869	-2.2927357573	-1.4047656270
H	-2.6964328947	-2.2716493946	-2.4877529530
H	-2.6964328959	-2.2716493954	2.4877529523
C	-1.7604239141	-3.0650726920	0.7264319366
C	-1.7604239137	-3.0650726917	-0.7264319371
H	1.7759351111	-3.0267253311	-2.4877940687
C	1.7932397928	-3.0568055807	-1.4048659507
C	1.7932397925	-3.0568055800	1.4048659513
C	0.6317899948	-3.4700819914	-0.7265499572
C	0.6317899946	-3.4700819909	0.7265499576
H	1.7759351105	-3.0267253302	2.4877940692
H	3.3231248865	1.2206530048	-2.4877068071
C	3.3516641087	1.2312767694	-1.4046060202
C	3.3516641094	1.2312767699	1.4046060198
H	3.3231248876	1.2206530057	2.4877068068
C	-0.6009607790	-3.4835698228	-1.4045607429
C	-0.6009607795	-3.4835698227	1.4045607429
H	-0.5958136687	-3.4507311413	2.4875251355
H	-0.5958136679	-3.4507311415	-2.4875251356
C	-3.3542480550	1.1966118115	-0.7265861403
C	-3.3542480547	1.1966118113	0.7265861404

C	-2.7412896832	2.2667819880	-1.4047629562
C	-2.7412896827	2.2667819874	1.4047629567
H	-2.7164498366	2.2458268910	2.4877461600
H	-2.7164498375	2.2458268921	-2.4877461596
C	-1.7879632872	3.0479921723	-0.7264304412
C	-1.7879632868	3.0479921719	0.7264304417
H	1.7487175676	3.0438327611	2.4877957851
C	1.7656373067	3.0738868099	1.4048580124
C	1.7656373061	3.0738868095	-1.4048580127
C	0.6002416873	3.4759313036	0.7265536123
C	0.6002416870	3.4759313034	-0.7265536123
H	1.7487175666	3.0438327604	-2.4877957854
C	2.7148889389	2.2875673871	0.7265759084
C	2.7148889385	2.2875673868	-0.7265759088
C	-0.6325669269	3.4775396138	1.4045657724
C	-0.6325669274	3.4775396142	-1.4045657721
H	-0.6271158266	3.4447802306	-2.4875273889
H	-0.6271158259	3.4447802300	2.4875273892
H	3.3336769437	-1.1893218167	2.4876966103
C	3.3623082614	-1.1996732811	1.4045985702
C	3.3623082613	-1.1996732815	-1.4045985700
C	3.5638646651	0.0167020485	0.7265727267
C	2.7350936929	-2.2616705557	0.7265742080
C	2.7350936928	-2.2616705559	-0.7265742075

C	3.5638646648	0.0167020483	-0.7265727267
H	3.3336769436	-1.1893218173	-2.4876966100

Table S43. Cartesian coordinates (in Å) for the TAO-DFT T_1 state of [10]cyclacene.

H	-3.8537538668	0.6392827016	2.4880672272
C	-3.8835077674	0.6437633789	1.4049665688
C	-3.8835077675	0.6437633830	-1.4049665697
C	-3.8868495160	-0.5874882152	0.7274497925
C	-3.8868495128	-0.5874882130	-0.7274497967
H	-3.8537538664	0.6392827088	-2.4880672283
C	-3.5253381624	-1.7644196082	1.4050006405
C	-3.5253381560	-1.7644196039	-1.4050006464
H	-3.4985865791	-1.7509829064	-2.4881469241
H	-3.4985865902	-1.7509829133	2.4881469184
C	-2.8042718146	-2.7626614789	0.7274908530
C	-2.8042718107	-2.7626614761	-0.7274908582
H	0.5814396003	-3.8640911402	-2.4880735386
C	0.5854892852	-3.8937596386	-1.4049596144
C	0.5854892827	-3.8937596359	1.4049596182
C	-0.6456799308	-3.8780051025	-0.7274382532
C	-0.6456799321	-3.8780051009	0.7274382543
H	0.5814395954	-3.8640911360	2.4880735424
H	3.4985864220	1.7509828588	-2.4881469762
C	3.5253380795	1.7644195857	-1.4050006616

C	3.5253380863	1.7644195903	1.4050006571
H	3.4985864332	1.7509828671	2.4881469719
C	-1.8168712569	-3.4983887722	-1.4050355071
C	-1.8168712615	-3.4983887741	1.4050355046
H	-1.8028646036	-3.4714017937	2.4881575755
H	-1.8028645948	-3.4714017906	-2.4881575780
C	-3.4869002198	1.8092513930	-0.7274461182
C	-3.4869002173	1.8092513898	0.7274461206
C	-2.7632346199	2.8053098998	-1.4050789205
C	-2.7632346153	2.8053098934	1.4050789250
H	-2.7437939837	2.7856009836	2.4882577992
H	-2.7437939910	2.7856009943	-2.4882577948
C	-1.7568537003	3.5147253745	-0.7274658174
C	-1.7568536975	3.5147253705	0.7274658219
H	1.8028646028	3.4714016283	2.4881576229
C	1.8168712741	3.4983886917	1.4050355185
C	1.8168712690	3.4983886886	-1.4050355217
C	0.6456799950	3.8780049838	0.7274382470
C	0.6456799927	3.8780049829	-0.7274382472
H	1.8028645943	3.4714016227	-2.4881576260
C	2.8042717199	2.7626613636	0.7274908439
C	2.8042717159	2.7626613605	-0.7274908486
C	-0.5854891763	3.8937596050	1.4049596259
C	-0.5854891802	3.8937596091	-1.4049596231

H	-0.5814394389	3.8640910889	-2.4880735648
H	-0.5814394324	3.8640910823	2.4880735674
H	2.7437940914	-2.7856008412	2.4882578076
C	2.7632346857	-2.8053098076	1.4050789280
C	2.7632346869	-2.8053098108	-1.4050789230
C	1.7568538174	-3.5147253653	0.7274658264
C	1.7568538180	-3.5147253663	-0.7274658209
H	2.7437940936	-2.7856008463	-2.4882578026
H	3.8537537747	-0.6392825618	2.4880672666
C	3.8835077206	-0.6437632894	1.4049665819
C	3.8835077186	-0.6437632924	-1.4049665813
C	3.8868493802	0.5874882484	0.7274497854
C	3.4869001901	-1.8092512631	0.7274461151
C	3.4869001888	-1.8092512640	-0.7274461114
C	3.8868493772	0.5874882466	-0.7274497876
H	3.8537537709	-0.6392825673	-2.4880672659

Table S44. Cartesian coordinates (in Å) for the TAO-DFT T_1 state of [11]cyclacene.

H	-3.9687160426	-1.6814935803	2.4877637485
C	-3.9916004096	-1.6913176986	1.4044404174
C	-3.9916004125	-1.6913176946	-1.4044404141
C	-3.3427248700	-2.7373873545	0.7272685741
C	-3.3427248694	-2.7373873509	-0.7272685752
H	-3.9687160484	-1.6814935731	-2.4877637452

C	-2.4361011472	-3.5690328821	1.4047637767
C	-2.4361011451	-3.5690328739	-1.4047637813
H	-2.4201077467	-3.5453508042	-2.4879069142
H	-2.4201077496	-3.5453508177	2.4879069097
C	-1.3266078382	-4.1016229557	0.7273459328
C	-1.3266078374	-4.1016229519	-0.7273459390
C	1.1072411412	-4.1659615850	-0.7272638262
C	1.1072411409	-4.1659615874	0.7272638209
C	-0.1144181208	-4.3150503509	-1.4044184648
C	-0.1144181215	-4.3150503555	1.4044184584
H	-0.1138136820	-4.2890370167	2.4877369918
H	-0.1138136807	-4.2890370075	-2.4877369981
C	-4.3015713826	-0.5002734984	-0.7273108655
C	-4.3015713774	-0.5002734994	0.7273108729
C	-4.2767680994	0.7302414329	-1.4046110436
C	-4.2767680869	0.7302414311	1.4046110523
H	-4.2500062131	0.7261447273	2.4878239153
H	-4.2500062329	0.7261447303	-2.4878239069
C	-3.8902497958	1.8986163441	-0.7273537449
C	-3.8902497902	1.8986163436	0.7273537518
H	-1.1029087930	4.1467173946	2.4878702279
C	-1.1091813879	4.1721361583	1.4046325745
C	-1.1091813883	4.1721361463	-1.4046325813
C	-2.2402168700	3.6874788965	0.7272658441

C	-2.2402168707	3.6874788922	-0.7272658466
H	-1.1029087940	4.1467173744	-2.4878702345
C	-3.2002392300	2.9179369991	1.4047180767
C	-3.2002392347	2.9179369957	-1.4047180742
H	-3.1816268091	2.9008700414	-2.4879824279
H	-3.1816268005	2.9008700472	2.4879824303
H	3.3280418490	2.7251735996	2.4879878949
C	3.3487769153	2.7423243104	1.4047811757
C	3.3487769191	2.7423243061	-1.4047811744
C	2.4316499621	3.5621722951	0.7272436532
C	2.4316499622	3.5621722906	-0.7272436567
H	3.3280418561	2.7251735923	-2.4879878935
C	1.3285331677	4.1076968365	1.4045193694
C	1.3285331668	4.1076968242	-1.4045193767
H	1.3212518410	4.0841326402	-2.4878302189
H	1.3212518422	4.0841326609	2.4878302118
C	0.1140209520	4.3060437938	0.7274156682
C	0.1140209517	4.3060437869	-0.7274156765
C	3.9851228999	1.6886149479	-0.7273978912
C	3.9851228948	1.6886149481	0.7273978972
C	4.3095236843	0.5013304807	-1.4045399307
C	4.3095236724	0.5013304803	1.4045399387
H	4.2832540308	0.4986164302	2.4878018093
H	4.2832540497	0.4986164310	-2.4878018015

C	4.2686375103	-0.7287744172	-0.7272449045
C	4.2686375052	-0.7287744176	0.7272449118
H	2.2295175441	-3.6705343509	2.4878341938
C	2.2440662304	-3.6947658452	1.4046732990
C	2.2440662303	-3.6947658389	-1.4046733022
C	3.1944855260	-2.9130834724	0.7272998063
C	3.1944855260	-2.9130834696	-0.7272998061
H	2.2295175443	-3.6705343405	-2.4878341968
C	3.8964862159	-1.9018426309	1.4044849207
C	3.8964862193	-1.9018426279	-1.4044849166
H	3.8728415922	-1.8901435702	-2.4877474955
H	3.8728415856	-1.8901435752	2.4877474995

Table S45. Cartesian coordinates (in Å) for the TAO-DFT T_1 state of [12]cyclacene.

H	-0.4011254473	4.6737270832	-2.4881082163
C	-0.4029122980	4.6977521911	-1.4047622963
C	-0.4029122635	4.6977521738	1.4047622981
C	0.8253003561	4.6342693266	-0.7277957484
C	0.8253003736	4.6342693392	0.7277957228
H	-0.4011253864	4.6737270597	2.4881082140
C	1.9998457630	4.2696294783	-1.4046759359
C	1.9998457974	4.2696295121	1.4046758876
H	1.9898782805	4.2484137011	2.4880254191
H	1.9898782186	4.2484136410	-2.4880254706

C	3.0321357046	3.6006755529	-0.7278166495
C	3.0321357270	3.6006755805	0.7278165947
C	4.4271971091	1.6027634790	0.7279382884
C	4.4271971123	1.6027634641	-0.7279383119
C	3.8658293570	2.6970510133	1.4050314200
C	3.8658293301	2.6970509718	-1.4050314667
H	3.8443284780	2.6820379848	-2.4882548008
H	3.8443285233	2.6820380571	2.4882547509
C	-1.6027633351	4.4271978822	0.7279383655
C	-1.6027633579	4.4271979043	-0.7279383354
C	-2.6970507727	3.8658297773	1.4050315145
C	-2.6970508217	3.8658298208	-1.4050314663
H	-2.6820378352	3.8443289848	-2.4882548682
H	-2.6820377512	3.8443289123	2.4882549114
C	-3.6006751127	3.0321358084	0.7278166653
C	-3.6006751445	3.0321358353	-0.7278166090
H	-4.6737263123	-0.4011254947	-2.4881081919
C	-4.6977513452	-0.4029123558	-1.4047622841
C	-4.6977513197	-0.4029123850	1.4047622871
C	-4.6342684884	0.8253002580	-0.7277957057
C	-4.6342684732	0.8253002416	0.7277957356
H	-4.6737262741	-0.4011255474	2.4881081908
C	-4.2696287966	1.9998457104	-1.4046758934
C	-4.2696287420	1.9998456678	1.4046759416

H	-4.2484129378	1.9898781515	2.4880254780
H	-4.2484130281	1.9898782247	-2.4880254348
H	0.4011256455	-4.6737271854	-2.4881082979
C	0.4029124871	-4.6977523049	-1.4047623306
C	0.4029124527	-4.6977522876	1.4047623324
C	-0.8253002394	-4.6342694996	-0.7277957811
C	-0.8253002569	-4.6342695122	0.7277957555
H	0.4011255846	-4.6737271618	2.4881082956
C	-1.9998457352	-4.2696296865	-1.4046759958
C	-1.9998457696	-4.2696297204	1.4046759476
H	-1.9898782382	-4.2484138922	2.4880255623
H	-1.9898781763	-4.2484138321	-2.4880256138
C	-3.0321357650	-3.6006757157	-0.7278166953
C	-3.0321357875	-3.6006757434	0.7278166405
C	-3.8658294994	-2.6970511140	1.4050314894
C	-3.8658294725	-2.6970510725	-1.4050315361
H	-3.8443286034	-2.6820380722	-2.4882549671
C	1.6027635300	-4.4271978916	0.7279383771
C	1.6027635528	-4.4271979137	-0.7279383470
C	2.6970509370	-3.8658296792	1.4050315141
C	2.6970509859	-3.8658297226	-1.4050314659
H	2.6820380079	-3.8443288799	-2.4882548671
H	2.6820379240	-3.8443288075	2.4882549103
C	3.6006751791	-3.0321356394	0.7278166530

C	3.6006752108	-3.0321356662	-0.7278165967
H	4.6737260993	0.4011255907	-2.4881080504
C	4.6977511237	0.4029124466	-1.4047622269
C	4.6977510981	0.4029124758	1.4047622299
C	4.6342683364	-0.8253000920	-0.7277956723
C	4.6342683212	-0.8253000756	0.7277957022
H	4.6737260611	0.4011256434	2.4881080493
C	4.2696287431	-1.9998455007	-1.4046758581
C	4.2696286886	-1.9998454581	1.4046759063
H	4.2484128985	-1.9898779378	2.4880253941
H	4.2484129888	-1.9898780110	-2.4880253508
C	-4.4271972741	-1.6027634786	0.7279383320
C	-4.4271972773	-1.6027634637	-0.7279383556
H	-3.8443286487	-2.6820381445	2.4882549172

Table S46. Cartesian coordinates (in Å) for the TAO-DFT T_1 state of [13]cyclacene.

H	-4.8887883034	1.4123686084	2.4880371917
C	-4.9100365559	1.4180901057	1.4046411089
C	-4.9100364084	1.4180904232	-1.4046423879
C	-5.0954904195	0.2027456209	0.7279055356
C	-5.0954903054	0.2027457535	-0.7279070373
H	-4.8887879195	1.4123691406	-2.4880385674
C	-4.9970994914	-1.0227202717	1.4046332034
C	-4.9970993239	-1.0227200717	-1.4046349486

H	-4.9749551393	-1.0185291440	-2.4880120189
H	-4.9749555448	-1.0185295284	2.4880101749
C	-4.5966195576	-2.1851072826	0.7279371489
C	-4.5966194120	-2.1851071903	-0.7279389390
C	-3.0464127565	-4.0683823258	-0.7277548028
C	-3.0464129264	-4.0683823747	0.7277531381
C	-3.9398466655	-3.2246200363	-1.4048241590
C	-3.9398469226	-3.2246201446	1.4048223303
H	-3.9222149552	-3.2101735715	2.4881664706
H	-3.9222144277	-3.2101733068	-2.4881683915
C	3.6918110928	-3.5345960990	0.7277845915
C	3.6918113657	-3.5345959156	-0.7277838382
C	4.4323767862	-2.5530317418	1.4045511308
C	4.4323773819	-2.5530314259	-1.4045498661
C	2.7433887093	-4.3168056288	-1.4046782296
C	2.7433881898	-4.3168059444	1.4046784324
H	2.7307182097	-4.2969160913	-2.4879898533
C	1.6292975757	-4.8366827358	-0.7278431563
C	1.6292973088	-4.8366828777	0.7278428657
H	-1.9769679016	-4.6697478212	2.4878457403
C	-1.9850734946	-4.6890646173	1.4044267224
C	-1.9850731210	-4.6890645287	-1.4044282108
C	-0.8027345156	-5.0259612491	0.7277866016
C	-0.8027342792	-5.0259611481	-0.7277877394

H	-1.9769672052	-4.6697475649	-2.4878473087
C	0.4252688622	-5.0856836697	1.4045155297
C	0.4252693467	-5.0856834803	-1.4045163067
H	0.4237721466	-5.0637610391	-2.4879193830
H	0.4237713059	-5.0637614475	2.4879185427
H	0.8016514120	5.0131744535	-2.4879627399
C	0.8048462650	5.0352837898	-1.4045763277
C	0.8048457150	5.0352836265	1.4045775657
H	0.8016504477	5.0131741900	2.4879639230
C	-0.4238275192	5.0795164815	0.7278788953
C	-0.4238272428	5.0795165508	-0.7278780956
C	-1.6299731415	4.8414834961	1.4045840313
C	-1.6299726648	4.8414837363	-1.4045837287
H	-1.6234927821	4.8210931834	-2.4879993893
H	-1.6234936465	4.8210928228	2.4879996209
C	-2.7378524788	4.3085108384	0.7278125875
C	-2.7378522484	4.3085109643	-0.7278127018
C	-4.4257182725	2.5481007987	-0.7278745765
C	-4.4257183968	2.5481006245	0.7278736797
C	-3.6945880918	3.5366375882	-1.4048129106
C	-3.6945884036	3.5366372592	1.4048123428
H	-3.6790162769	3.5217349088	2.4882039724
H	-3.6790156626	3.5217354227	-2.4882046265
H	3.0369597733	4.0572458748	2.4881157407

C	3.0511048686	4.0760635913	1.4048024171
C	3.0511055144	4.0760638627	-1.4048003875
C	1.9837727719	4.6864014420	0.7279245371
C	1.9837730706	4.6864015275	-0.7279228523
H	3.0369608865	4.0572463302	-2.4881137569
C	3.9340524770	3.2212640180	-0.7277597145
C	3.9340521020	3.2212638498	0.7277619978
C	4.5999964764	2.1875898054	-1.4044039810
C	4.5999957318	2.1875894748	1.4044063550
H	4.5807590462	2.1784262500	2.4878064851
H	4.5807603392	2.1784268366	-2.4878041497
C	4.9872434083	1.0207614475	-0.7277556571
C	4.9872430071	1.0207612795	0.7277579670
C	4.9027184170	-1.4171325186	0.7277933884
C	4.9027187477	-1.4171323544	-0.7277916551
C	5.0996137222	-0.2035130425	1.4045132836
C	5.0996144515	-0.2035127335	-1.4045111958
H	5.0778186492	-0.2028294102	-2.4879163904
H	5.0778173855	-0.2028299593	2.4879184420
H	4.4125418650	-2.5415794463	-2.4878895687
H	4.4125408384	-2.5415800113	2.4878907950
H	2.7307173147	-4.2969166630	2.4879900075

Table S47. Cartesian coordinates (in Å) for the TAO-DFT T_1 state of [14]cyclacene.

H	-3.4943790497	4.2117969362	-2.4883577285
C	-3.5079786833	4.2280831270	-1.4049688451
C	-3.5079785816	4.2280829690	1.4049689882
C	-2.4812399713	4.9038915496	-0.7281910470
C	-2.4812399264	4.9038914780	0.7281911887
H	-3.4943788842	4.2117966803	2.4883578716
C	-1.3333643499	5.3431035724	-1.4047794634
C	-1.3333642729	5.3431034611	1.4047795713
H	-1.3286553031	5.3228386226	2.4882087066
H	-1.3286554438	5.3228388036	-2.4882086000
C	-0.1146519775	5.5015010468	-0.7281470653
C	-0.1146519479	5.5015010400	0.7281471157
C	2.2751276671	5.0040881559	0.7281847890
C	2.2751276470	5.0040881552	-0.7281848406
C	1.1096733611	5.3943110748	1.4047433727
C	1.1096733006	5.3943110422	-1.4047433764
H	1.1057913239	5.3737808182	-2.4881691777
H	1.1057914328	5.3737808464	2.4881691744
H	4.9646529922	-2.2713691752	-2.4879426187
C	4.9826424884	-2.2793864272	-1.4045059241
C	4.9826424409	-2.2793863363	1.4045059354
C	4.3490167608	-3.3326105105	-0.7280071701
C	4.3490167292	-3.3326104546	0.7280072313
H	4.9646529192	-2.2713690142	2.4879426309

C	3.5079787815	-4.2280820130	-1.4049687084
C	3.5079786909	-4.2280818601	1.4049688147
C	5.3550899271	-1.1081604370	0.7280369546
C	5.3550899238	-1.1081604671	-0.7280370063
C	5.4730923897	0.1151859798	1.4046096973
C	5.4730923247	0.1151859391	-1.4046097882
H	5.4529473196	0.1148309533	-2.4880428303
H	5.4529474121	0.1148310246	2.4880427401
C	5.3055854567	1.3327496767	0.7280361875
C	5.3055854382	1.3327496641	-0.7280363057
H	3.3161342020	4.3549738100	-2.4882888861
C	3.3294346801	4.3722127176	-1.4049234947
C	3.3294346967	4.3722126955	1.4049234147
C	4.2081942758	3.5136658492	-0.7280069537
C	4.2081942870	3.5136658486	0.7280068469
H	3.3161342446	4.3549737795	2.4882888083
C	4.8851658830	2.4876461068	-1.4044971666
C	4.8851658925	2.4876461076	1.4044970515
H	4.8673104688	2.4787042640	2.4879201002
H	4.8673104396	2.4787042602	-2.4879202149
H	-4.8673115380	-2.4787054369	2.4879206130
C	-4.8851670697	-2.4876473639	1.4044972083
C	-4.8851669070	-2.4876472851	-1.4044973340
H	-4.8673112761	-2.4787053055	-2.4879207379

C	-5.3055862028	-1.3327504190	-0.7280364464
C	-5.3055862786	-1.3327504549	0.7280363379
C	-5.4730927256	-0.1151863301	-1.4046099935
C	-5.4730928041	-0.1151863886	1.4046099257
H	-5.4529475521	-0.1148314357	2.4880433978
H	-5.4529474047	-0.1148313312	-2.4880434640
C	-5.3550897634	1.1081603524	-0.7280371493
C	-5.3550897993	1.1081603230	0.7280371383
C	-4.3490164335	3.3326109796	0.7280073957
C	-4.3490164653	3.3326110420	-0.7280072867
C	-4.9826420861	2.2793864888	1.4045061886
C	-4.9826420623	2.2793865580	-1.4045061372
H	-4.9646522412	2.2713691448	-2.4879432492
H	-4.9646522670	2.2713690162	2.4879433007
H	-3.3161343996	-4.3549747719	-2.4882891957
C	-3.3294349171	-4.3722138172	-1.4049235948
C	-3.3294349847	-4.3722138125	1.4049235237
C	-4.2081949332	-3.5136670709	-0.7280070608
C	-4.2081949999	-3.5136671001	0.7280069506
H	-3.3161345244	-4.3549747793	2.4882891277
C	-2.2751274401	-5.0040886410	0.7281848471
C	-2.2751274106	-5.0040886573	-0.7281848704
C	-1.1096728397	-5.3943109128	1.4047433919
C	-1.1096727803	-5.3943109446	-1.4047433621

H	-1.1057908792	-5.3737807399	-2.4881691885
H	-1.1057909755	-5.3737806687	2.4881692180
C	0.1146524952	-5.5015001710	0.7281471016
C	0.1146525259	-5.5015002259	-0.7281470250
C	2.4812401806	-4.9038902171	-0.7281909668
C	2.4812401404	-4.9038901418	0.7281910865
C	1.3333647413	-5.3431022068	-1.4047793840
C	1.3333646692	-5.3431020505	1.4047794979
H	1.3286556761	-5.3228372996	2.4882084651
H	1.3286557935	-5.3228375601	-2.4882083532
H	3.4943790931	-4.2117957312	2.4883573721
H	3.4943792395	-4.2117959945	-2.4883572680

7.4 KS-DFT Triplet States

Table S48. Cartesian coordinates (in Å) for the KS-DFT T_1 state of [6]cyclacene.

H	2.1216305034	-1.0096912609	2.4890296313
C	2.1686150147	-1.0323830798	1.4056383347
C	2.1686150147	-1.0323830798	-1.4056383347
C	2.3855080166	0.1902074213	0.7262894619
C	2.3855080166	0.1902074213	-0.7262894619
H	2.1216305035	-1.0096912609	-2.4890296313
H	0.1864074316	-2.3488319464	2.4891828590
C	0.1901290864	-2.3977856220	1.4055190089
C	0.1901290864	-2.3977856220	-1.4055190089
C	1.3571647807	-1.9726388126	0.7263516274
C	1.3571647807	-1.9726388126	-0.7263516274
H	0.1864074316	-2.3488319464	-2.4891828590
C	1.9800016289	1.3641508002	1.4056406718
C	1.9800016289	1.3641508002	-1.4056406718
H	1.9403363945	1.3366105497	-2.4893170577
H	1.9403363945	1.3366105497	2.4893170577
C	-1.0294723971	-2.1633303888	0.7261823964

C	-1.0294723971	-2.1633303888	-0.7261823964
C	1.0294724728	2.1633304612	0.7261823267
C	1.0294724728	2.1633304612	-0.7261823267
H	-2.1216305537	1.0096912496	-2.4890296505
C	-2.1686149988	1.0323831904	-1.4056383509
C	-2.1686149988	1.0323831904	1.4056383509
C	-1.3571646523	1.9726389486	-0.7263516048
C	-1.3571646523	1.9726389487	0.7263516048
H	-2.1216305537	1.0096912496	2.4890296505
H	-1.9403365434	-1.3366106632	-2.4893171559
C	-1.9800017789	-1.3641508612	-1.4056407522
C	-1.9800017789	-1.3641508612	1.4056407522
C	-2.3855079604	-0.1902072692	-0.7262895074
C	-2.3855079604	-0.1902072692	0.7262895074
H	-1.9403365434	-1.3366106632	2.4893171559
C	-0.1901290058	2.3977854953	-1.4055189463
C	-0.1901290058	2.3977854954	1.4055189463
H	-0.1864074392	2.3488317879	2.4891827818
H	-0.1864074392	2.3488317878	-2.4891827818

Table S49. Cartesian coordinates (in Å) for the KS-DFT T_1 state of [7]cyclacene.

C	-0.3483393239	2.7470000595	-1.4065534276
C	-0.3483393125	2.7469997889	1.4065534658
C	0.8530070537	2.6164282620	-0.7279279112

C	0.8530070107	2.6164282130	0.7279280397
H	-0.3434357618	2.6922791807	2.4899787931
H	-2.3361178511	1.4290426952	-2.4877221624
C	-2.3913752307	1.4639215680	-1.4049494012
C	-2.3913748071	1.4639212983	1.4049493985
C	-1.5426186571	2.3452114309	-0.7237208577
C	-1.5426185712	2.3452112581	0.7237210370
H	-2.3361174862	1.4290424809	2.4877217598
C	1.9406712523	1.9637946843	-1.4097694309
C	1.9406711619	1.9637946526	1.4097695052
H	1.9188846621	1.9445536551	2.4940683456
H	1.9188847758	1.9445537657	-2.4940684005
C	-2.8021586253	0.2925620693	-0.7228206955
C	-2.8021583818	0.2925620429	0.7228208875
C	2.5843117658	0.9369350770	-0.7356060584
C	2.5843117651	0.9369350932	0.7356061446
C	-2.6216527055	-0.9365227413	1.4052174871
C	-2.6216531318	-0.9365229013	-1.4052174995
C	2.8315893215	-0.2948094060	1.4088961897
C	2.8315894371	-0.2948094224	-1.4088960951
C	-0.8843844730	-2.6125703097	-1.4066804484
C	-0.8843844265	-2.6125700744	1.4066804935
C	-1.9683694948	-1.9684789716	-0.7239807677
C	-1.9683693941	-1.9684788318	0.7239809359

H	-2.5575003926	-0.9126006877	2.4876907175
H	-2.5575007546	-0.9126008008	-2.4876911210
H	2.8432823265	-0.2953265584	2.4935961693
C	2.3524724605	-1.4533469971	-0.7363557602
C	2.3524724601	-1.4533470110	0.7363558501
C	1.5191644847	-2.3366950913	-1.4099197324
C	1.5191644047	-2.3366950400	1.4099198215
H	-0.8678572312	-2.5574459036	2.4898436114
H	-0.8678573125	-2.5574461247	-2.4898438268
H	-0.3434358196	2.6922794486	-2.4899790312
C	0.3164153732	-2.7423177081	-0.7284511416
C	0.3164153396	-2.7423176575	0.7284512797
H	2.8432825763	-0.2953265888	-2.4935961335
H	1.5046880397	-2.3189457035	2.4944754114
H	1.5046881277	-2.3189458465	-2.4944754411

Table S50. Cartesian coordinates (in Å) for the KS-DFT T_1 state of [8]cyclacene.

H	-2.8674208479	1.2433445022	2.4920847597
C	-2.9034037570	1.2587709514	1.4079720127
C	-2.9034037570	1.2587709514	-1.4079720127
C	-3.1575986311	0.0517145528	0.7295984230
C	-3.1575986311	0.0517145528	-0.7295984230
H	-2.8674208480	1.2433445022	-2.4920847597
C	-2.9434513031	-1.1629846323	1.4079244559

C	-2.9434513032	-1.1629846323	-1.4079244559
H	-2.9070334999	-1.1485397203	-2.4920245601
H	-2.9070334999	-1.1485397203	2.4920245600
C	-2.2692288936	-2.1960509194	0.7296363224
C	-2.2692288936	-2.1960509194	-0.7296363224
H	1.1485398458	-2.9070331705	-2.4920244440
C	1.1629845536	-2.9434508791	-1.4079244014
C	1.1629845536	-2.9434508790	1.4079244014
C	-0.0517145441	-3.1575980875	-0.7295983257
C	-0.0517145441	-3.1575980875	0.7295983257
H	1.1485398458	-2.9070331705	2.4920244439
H	2.8674208263	-1.2433445156	-2.4920847335
C	2.9034037319	-1.2587709590	-1.4079719987
C	2.9034037319	-1.2587709590	1.4079719987
C	2.1960510438	-2.2692288475	-0.7296363298
C	2.1960510438	-2.2692288475	0.7296363298
H	2.8674208263	-1.2433445156	2.4920847335
C	-1.2587708160	-2.9034032391	-1.4079719390
C	-1.2587708160	-2.9034032390	1.4079719390
H	-1.2433445653	-2.8674204631	2.4920846036
H	-1.2433445653	-2.8674204631	-2.4920846037
C	-2.1960510593	2.2692288349	-0.7296363442
C	-2.1960510593	2.2692288349	0.7296363442
C	-1.1629845723	2.9434509111	-1.4079244309

C	-1.1629845723	2.9434509111	1.4079244309
H	-1.1485398613	2.9070331911	2.4920244990
H	-1.1485398613	2.9070331911	-2.4920244989
C	0.0517145473	3.1575980909	-0.7295983447
C	0.0517145473	3.1575980910	0.7295983447
H	2.9070335164	1.1485397041	2.4920245771
C	2.9434513212	1.1629846212	1.4079244649
C	2.9434513212	1.1629846212	-1.4079244649
C	2.2692289120	2.1960509055	0.7296363345
C	2.2692289120	2.1960509055	-0.7296363345
H	2.9070335164	1.1485397041	-2.4920245770
C	3.1575986290	-0.0517145827	0.7295984212
C	3.1575986290	-0.0517145827	-0.7295984212
C	1.2587708385	2.9034032693	1.4079719661
C	1.2587708384	2.9034032693	-1.4079719661
H	1.2433445851	2.8674204814	-2.4920846544
H	1.2433445851	2.8674204814	2.4920846544

Table S51. Cartesian coordinates (in Å) for the KS-DFT T_1 state of [9]cyclacene.

H	-3.5366272723	-0.0171677832	2.4910517342
C	-3.5861548756	-0.0173403932	1.4073276909
C	-3.5861557460	-0.0173402886	-1.4073286354
C	-3.3715007765	-1.2283316907	0.7266614208
C	-3.3715008096	-1.2283318642	-0.7266619150

H	-3.5366272348	-0.0171676120	-2.4910534181
C	-2.7253347282	-2.2877509797	1.4075694183
C	-2.7253353774	-2.2877521090	-1.4075698132
H	-2.6888088622	-2.2559344447	-2.4912379772
H	-2.6888087653	-2.2559332732	2.4912368987
C	-1.7823164162	-3.0580248509	0.7271781976
C	-1.7823164909	-3.0580254717	-0.7271778046
H	1.7789662938	-3.0136487641	-2.4935545155
C	1.7959658946	-3.0405814414	-1.4091652721
C	1.7959658077	-3.0405802276	1.4091658961
C	0.6104689010	-3.4609129726	-0.7292355683
C	0.6104689055	-3.4609122512	0.7292364645
H	1.7789663503	-3.0136470230	2.4935548822
H	3.3692028372	1.2315978925	-2.4948396241
C	3.3750015425	1.2326541949	-1.4100083579
C	3.3750026071	1.2326544389	1.4100081505
H	3.3692047171	1.2315981785	2.4948393470
C	-0.5987962024	-3.4644305752	-1.4077654917
C	-0.5987960785	-3.4644287803	1.4077661033
H	-0.5921500803	-3.4196307678	2.4916165437
H	-0.5921501437	-3.4196330989	-2.4916164438
C	-3.3813348269	1.1943979410	-0.7266870734
C	-3.3813347495	1.1943976945	0.7266867099
C	-2.7438564733	2.2601303354	-1.4075785089

C	-2.7438557822	2.2601290180	1.4075783328
H	-2.7070546769	2.2286190705	2.4912481260
H	-2.7070548817	2.2286205494	-2.4912489775
C	-1.8085712323	3.0384524641	-0.7272656264
C	-1.8085710921	3.0384517902	0.7272662465
H	1.7546754879	3.0343974844	2.4937109979
C	1.7704825942	3.0597103447	1.4092597546
C	1.7704825860	3.0597111139	-1.4092591436
C	0.5800566880	3.4662244330	0.7294725259
C	0.5800566024	3.4662250800	-0.7294715559
H	1.7546751507	3.0343985744	-2.4937106185
C	2.7030804738	2.2879081155	0.7335333360
C	2.7030800529	2.2879082274	-0.7335331241
C	-0.6287720912	3.4576150393	1.4078335426
C	-0.6287721988	3.4576167777	-1.4078327563
H	-0.6218529561	3.4135341521	-2.4917107461
H	-0.6218528343	3.4135319124	2.4917110421
H	3.3767844188	-1.1975621707	2.4947851927
C	3.3836299367	-1.1988045859	1.4099589859
C	3.3836290489	-1.1988048454	-1.4099591344
C	3.5578113875	0.0165781922	0.7363072524
C	2.7218020077	-2.2618522424	0.7331766282
C	2.7218017238	-2.2618524798	-0.7331763576
C	3.5578106528	0.0165781575	-0.7363075436

H	3.3767828773	-1.1975624834	-2.4947854181

Table S52. Cartesian coordinates (in Å) for the KS-DFT T_1 state of [10]cyclacene.

H	-3.8445919245	0.6373397362	2.4930625838
C	-3.8784775107	0.6425064274	1.4087520887
C	-3.8784775107	0.6425064274	-1.4087520887
C	-3.8857518247	-0.5878485293	0.7311593986
C	-3.8857518247	-0.5878485293	-0.7311593986
H	-3.8445919245	0.6373397362	-2.4930625839
C	-3.5225409928	-1.7634276245	1.4087466299
C	-3.5225409928	-1.7634276245	-1.4087466299
H	-3.4929200194	-1.7485138032	-2.4931395855
H	-3.4929200194	-1.7485138032	2.4931395854
C	-2.8047454486	-2.7630813908	0.7312398562
C	-2.8047454486	-2.7630813908	-0.7312398562
H	0.5797157755	-3.8552111457	-2.4930691550
C	0.5843677162	-3.8888326131	-1.4087370321
C	0.5843677162	-3.8888326131	1.4087370321
C	-0.6459713493	-3.8767329431	-0.7311471476
C	-0.6459713493	-3.8767329431	0.7311471476
H	0.5797157755	-3.8552111457	2.4930691550
H	3.4929199782	1.7485137871	-2.4931395742
C	3.5225408969	1.7634276288	-1.4087466260
C	3.5225408969	1.7634276288	1.4087466260

H	3.4929199783	1.7485137871	2.4931395742
C	-1.8157197573	-3.4953120801	-1.4087948748
C	-1.8157197573	-3.4953120801	1.4087948748
H	-1.8000709035	-3.4652422179	2.4931527930
H	-1.8000709035	-3.4652422179	-2.4931527930
C	-3.4854652201	1.8083133256	-0.7311549186
C	-3.4854652201	1.8083133256	0.7311549185
C	-2.7602284831	2.8022685290	-1.4087816004
C	-2.7602284831	2.8022685290	1.4087816004
H	-2.7392432054	2.7809927277	2.4932555803
H	-2.7392432053	2.7809927277	-2.4932555803
C	-1.7560275461	3.5134339067	-0.7311799148
C	-1.7560275462	3.5134339067	0.7311799148
H	1.8000708860	3.4652422498	2.4931527288
C	1.8157196667	3.4953120098	1.4087948466
C	1.8157196667	3.4953120098	-1.4087948465
C	0.6459713219	3.8767330159	0.7311471252
C	0.6459713219	3.8767330159	-0.7311471252
H	1.8000708860	3.4652422498	-2.4931527288
C	2.8047453899	2.7630814607	0.7312398399
C	2.8047453899	2.7630814607	-0.7312398399
C	-0.5843677257	3.8888325204	1.4087369967
C	-0.5843677257	3.8888325204	-1.4087369966
H	-0.5797158147	3.8552111807	-2.4930690702

H	-0.5797158147	3.8552111807	2.4930690702
H	2.7392432177	-2.7809927928	2.4932556665
C	2.7602285836	-2.8022686345	1.4087816346
C	2.7602285836	-2.8022686345	-1.4087816346
C	1.7560275589	-3.5134338671	0.7311799370
C	1.7560275589	-3.5134338672	-0.7311799370
H	2.7392432177	-2.7809927928	-2.4932556665
H	3.8445919773	-0.6373397765	2.4930626377
C	3.8784776029	-0.6425064221	1.4087521105
C	3.8784776029	-0.6425064221	-1.4087521105
C	3.8857518359	0.5878486283	0.7311594022
C	3.4854653182	-1.8083132932	0.7311549415
C	3.4854653182	-1.8083132932	-0.7311549416
C	3.8857518358	0.5878486283	-0.7311594023
H	3.8445919772	-0.6373397765	-2.4930626378

Table S53. Cartesian coordinates (in Å) for the KS-DFT T_1 state of [11]cyclacene.

H	-3.9673158966	-1.6773832975	2.4890188289
C	-3.9911116317	-1.6876853658	1.4056142354
C	-3.9886116451	-1.6939853663	-1.4028142307
C	-3.3393805470	-2.7382485754	0.7298978633
C	-3.3407805490	-2.7349485740	-0.7265978613
H	-3.9612159144	-1.6800832954	-2.4853188292
C	-2.4380344019	-3.5722540737	1.4068371459

C	-2.4312344061	-3.5674540746	-1.4107371498
H	-2.4155883094	-3.5424995329	-2.4929594956
H	-2.4190883051	-3.5452995363	2.4904594870
C	-1.3239894949	-4.1011603025	0.7282757530
C	-1.3263894952	-4.0985603020	-0.7292757566
C	1.1050664476	-4.1657979108	-0.7277917772
C	1.1081664464	-4.1645979100	0.7286917743
C	-0.1104951327	-4.3127153522	-1.4114912344
C	-0.1170951331	-4.3146153458	1.4056912284
H	-0.1166780328	-4.2884636354	2.4890905648
H	-0.1094780319	-4.2859636385	-2.4937905756
C	-4.3004643266	-0.4976996707	-0.7261400550
C	-4.3000643179	-0.5019996713	0.7290400657
C	-4.2760386039	0.7245015246	-1.4056851025
C	-4.2755385799	0.7347015210	1.4037851125
H	-4.2521366671	0.7286276233	2.4861783263
H	-4.2591366998	0.7196276283	-2.4890783214
C	-3.8877007809	1.9009937658	-0.7285859052
C	-3.8936007724	1.8949937640	0.7268859154
H	-1.0476892450	4.1312151622	2.4858255951
C	-1.0988706845	4.1701505268	1.4038065996
C	-1.1120706876	4.1691505252	-1.4043066057
C	-2.2444498842	3.6857749751	0.7251980336
C	-2.2348498866	3.6902749735	-0.7274980333

H	-1.1033892490	4.1485151534	-2.4858256058
C	-3.2034100629	2.9255708647	1.4032908968
C	-3.2024100757	2.9133708686	-1.4043908934
H	-3.1718870207	2.8940184878	-2.4871371251
H	-3.1799870033	2.9089184856	2.4872371236
H	3.3331600941	2.7246734171	2.4923418644
C	3.3455262263	2.7350977032	1.4073542771
C	3.3440262387	2.7447977060	-1.4034542747
C	2.4270533518	3.5625879948	0.7281749522
C	2.4334533538	3.5582879929	-0.7270749528
H	3.3149601105	2.7189734175	-2.4873418668
C	1.3350738661	4.1061070577	1.4056932182
C	1.3212738686	4.1085070558	-1.4017932247
H	1.3181602902	4.0727306443	-2.4840860092
H	1.3285602877	4.0857306535	2.4896859979
C	0.1096030520	4.3067390596	0.7271487941
C	0.1138030518	4.3051390559	-0.7272487998
C	3.9861591661	1.6853249076	-0.7281301097
C	3.9933591578	1.6923249065	0.7285301189
C	4.3060790310	0.5037320532	-1.4057135061
C	4.3092790074	0.4961320514	1.4032135155
H	4.2757664094	0.4896636493	2.4854559057
H	4.2686664414	0.4970636516	-2.4890559014
C	4.2677437167	-0.7315663579	-0.7298724078

C	4.2668437081	-0.7272663576	0.7242724183
H	2.2252500007	-3.6709391655	2.4896866658
C	2.2405345621	-3.6954607207	1.4065467408
C	2.2464345678	-3.6935607238	-1.4036467433
C	3.1946662347	-2.9116160897	0.7306292449
C	3.1933662373	-2.9149160892	-0.7258292418
H	2.2319500075	-3.6708391656	-2.4872866731
C	3.8911213449	-1.8994726093	1.4047586744
C	3.8948213586	-1.9002726114	-1.4053586692
H	3.8664695533	-1.8922714460	-2.4894013897
H	3.8446695349	-1.8297714453	2.4868013900

Table S54. Cartesian coordinates (in Å) for the KS-DFT T_1 state of [12]cyclacene.

H	-0.4004480590	4.6655103738	-2.4936906381
C	-0.4024358820	4.6927148853	-1.4090879453
C	-0.4024345545	4.6927153156	1.4090881168
C	0.8249753619	4.6322618616	-0.7319237006
C	0.8249753500	4.6322622168	0.7319241632
H	-0.4004468754	4.6655110238	2.4936908157
C	1.9977793777	4.2653048949	-1.4090016961
C	1.9977809183	4.2653056543	1.4090024773
H	1.9865330060	4.2413350522	2.4936096801
H	1.9865311518	4.2413333691	-2.4936088711
C	3.0308754798	3.5992529714	-0.7319643326

C	3.0308758908	3.5992535368	0.7319652966
C	4.4252988469	1.6020374112	0.7320849373
C	4.4252980404	1.6020371108	-0.7320837320
C	3.8610897797	2.6937698795	1.4093806907
C	3.8610878562	2.6937691579	-1.4093794962
H	3.8358168441	2.6760404407	-2.4938104692
H	3.8358198942	2.6760420155	2.4938117092
C	-1.6020368773	4.4252977936	0.7320844464
C	-1.6020368407	4.4252979214	-0.7320845940
C	-2.6937687395	3.8610884877	1.4093802006
C	-2.6937702536	3.8610884289	-1.4093806755
H	-2.6760419211	3.8358180350	-2.4938120151
H	-2.6760401633	3.8358175454	2.4938115202
C	-3.5992534741	3.0308751613	0.7319646635
C	-3.5992538415	3.0308755544	-0.7319653769
H	-4.6655136970	-0.4004479004	-2.4936919248
C	-4.6927175220	-0.4024356162	-1.4090889594
C	-4.6927154041	-0.4024352471	1.4090876855
C	-4.6322635384	0.8249752183	-0.7319246291
C	-4.6322627471	0.8249750428	0.7319235112
H	-4.6655100491	-0.4004474132	2.4936906065
C	-4.2653070799	1.9977799075	-1.4090029263
C	-4.2653051660	1.9977799361	1.4090019241
H	-4.2413333193	1.9865314960	2.4936094022

H	-4.2413363199	1.9865320841	-2.4936104393
H	0.4004473495	-4.6655113447	-2.4936918722
C	0.4024351344	-4.6927160673	-1.4090886316
C	0.4024364622	-4.6927156376	1.4090884595
C	-0.8249752699	-4.6322630399	-0.7319245264
C	-0.8249752816	-4.6322626849	0.7319240636
H	0.4004485337	-4.6655106959	2.4936916939
C	-1.9977813073	-4.2653065488	-1.4090031603
C	-1.9977797661	-4.2653057893	1.4090023792
H	-1.9865313249	-4.2413337747	2.4936102430
H	-1.9865331800	-4.2413354577	-2.4936110521
C	-3.0308765671	-3.5992540398	-0.7319656814
C	-3.0308761557	-3.5992534742	0.7319647179
C	-3.8610888007	-2.6937693502	1.4093801541
C	-3.8610907253	-2.6937700723	-1.4093813478
H	-3.8358204281	-2.6760419602	-2.4938129923
C	1.6020375915	-4.4252982211	0.7320848081
C	1.6020376279	-4.4252980929	-0.7320846612
C	2.6937711317	-3.8610882725	1.4093808590
C	2.6937696175	-3.8610883308	-1.4093803847
H	2.6760408994	-3.8358173462	-2.4938119130
H	2.6760426574	-3.8358178368	2.4938124071
C	3.5992543651	-3.0308750718	0.7319653603
C	3.5992539976	-3.0308746784	-0.7319646472

H	4.6655097496	0.4004479521	-2.4936897131
C	4.6927147750	0.4024358092	-1.4090872209
C	4.6927168919	0.4024361779	1.4090884955
C	4.6322625620	-0.8249743943	-0.7319233316
C	4.6322633529	-0.8249745699	0.7319244500
H	4.6655133959	0.4004484387	2.4936910318
C	4.2653053804	-1.9977791981	-1.4090017433
C	4.2653072939	-1.9977791698	1.4090027455
H	4.2413366771	-1.9865314897	2.4936101111
H	4.2413336772	-1.9865309011	-2.4936090741
C	-4.4252988031	-1.6020368942	0.7320840389
C	-4.4252996103	-1.6020371948	-0.7320852433
H	-3.8358173765	-2.6760403848	2.4938117529

Table S55. Cartesian coordinates (in Å) for the KS-DFT T_1 state of [13]cyclacene.

H	-4.8943575515	1.4157918821	2.4891371192
C	-4.9171120073	1.4222709932	1.4043849092
C	-4.9171152600	1.4222693915	-1.4043829690
C	-5.1063742525	0.2024086837	0.7260177183
C	-5.1063769796	0.2024082959	-0.7260136458
H	-4.8943631270	1.4157882680	-2.4891354944
C	-5.0013413749	-1.0229507795	1.4041769725
C	-5.0013476564	-1.0229518901	-1.4041726078
H	-4.9756676199	-1.0179804923	-2.4888608879

H	-4.9756577233	-1.0179782609	2.4888644489
C	-4.6062216209	-2.1863193036	0.7261915077
C	-4.6062246611	-2.1863197906	-0.7261870004
C	-3.0554197684	-4.0717784209	-0.7271554135
C	-3.0554184193	-4.0717790071	0.7271558256
C	-3.9438015042	-3.2294228884	-1.4046799477
C	-3.9437966563	-3.2294225235	1.4046820900
H	-3.9258754378	-3.2141711720	2.4894128235
H	-3.9258830930	-3.2141710592	-2.4894115438
C	3.6904876166	-3.5401663910	0.7264181824
C	3.6904870956	-3.5401641532	-0.7264207153
C	4.4404285570	-2.5528398696	1.4019919423
C	4.4404313363	-2.5528363825	-1.4019913301
C	2.7463275504	-4.3177711616	-1.4024143603
C	2.7463285749	-4.3177786130	1.4024100613
H	2.7351805884	-4.3000859795	-2.4873216282
C	1.6259949762	-4.8415299654	-0.7268463606
C	1.6259957990	-4.8415341038	0.7268401664
H	-1.9813172968	-4.6819883942	2.4893161644
C	-1.9877722455	-4.6978994701	1.4044194773
C	-1.9877738158	-4.6978958895	-1.4044221086
C	-0.8088396080	-5.0310528729	0.7277201959
C	-0.8088397463	-5.0310497310	-0.7277247943
H	-1.9813205801	-4.6819814851	-2.4893191605

C	0.4260205481	-5.0922990594	1.4036618014
C	0.4260198274	-5.0922910770	-1.4036674533
H	0.4253014680	-5.0743931383	-2.4886105076
H	0.4253038499	-5.0744062915	2.4886052993
H	0.8025394487	5.0164739429	-2.4875446730
C	0.8048900412	5.0358676800	-1.4025888435
C	0.8048866258	5.0358735750	1.4025864824
H	0.8025341613	5.0164835114	2.4875423904
C	-0.4305580398	5.0824781834	0.7271446563
C	-0.4305571860	5.0824745596	-0.7271494018
C	-1.6341367808	4.8494116647	1.4040066982
C	-1.6341334985	4.8494041053	-1.4040117543
H	-1.6287332722	4.8332025693	-2.4889523143
H	-1.6287378866	4.8332151318	2.4889476321
C	-2.7482735762	4.3127716037	0.7277754216
C	-2.7482724132	4.3127684045	-0.7277803271
C	-4.4381638152	2.5500456067	-0.7270045291
C	-4.4381633560	2.5500468733	0.7270040303
C	-3.7030238749	3.5457071542	-1.4048678089
C	-3.7030252630	3.5457117886	1.4048649830
H	-3.6897833368	3.5327401876	2.4897254557
H	-3.6897819022	3.5327318472	-2.4897281098
H	3.0442870195	4.0612087812	2.4871969392
C	3.0552890911	4.0748983978	1.4021931760

C	3.0552951606	4.0748975968	-1.4021900896
C	1.9783958710	4.6880250489	0.7264418457
C	1.9783969463	4.6880238910	-0.7264416558
H	3.0442966836	4.0612078955	-2.4871941818
C	3.9317749417	3.2242950489	-0.7278459099
C	3.9317715077	3.2242937499	0.7278516210
C	4.6136675117	2.1897542558	-1.4023445063
C	4.6136575113	2.1897521429	1.4023514324
H	4.6075474412	2.1877631756	2.4875097417
H	4.6075640268	2.1877674569	-2.4875031351
C	4.9889494008	1.0207615717	-0.7298730460
C	4.9889445433	1.0207602622	0.7298802290
C	4.9044254018	-1.4220228019	0.7284810105
C	4.9044280168	-1.4220221136	-0.7284774342
C	5.1164200020	-0.2026873271	1.4026129105
C	5.1164289160	-0.2026854807	-1.4026071333
H	5.1110070050	-0.2030998521	-2.4877936994
H	5.1109919982	-0.2031030413	2.4877992663
H	4.4268480543	-2.5457559371	-2.4870317118
H	4.4268433021	-2.5457616668	2.4870324840
H	2.7351823940	-4.3000977772	2.4873180268

Table S56. Cartesian coordinates (in Å) for the KS-DFT T_1 state of [14]cyclacene.

H	-3.4898719016	4.2037327798	-2.4943341846

C	-3.5058359525	4.2227172010	-1.4097012337
C	-3.5058362148	4.2227175164	1.4097013188
C	-2.4809814702	4.8995936392	-0.7325775535
C	-2.4809815382	4.8995937872	0.7325775462
H	-3.4898722143	4.2037331520	2.4943344510
C	-1.3325501122	5.3344794921	-1.4095228506
C	-1.3325501885	5.3344798303	1.4095229229
H	-1.3268773320	5.3105149286	2.4941869676
H	-1.3268772294	5.3105145307	-2.4941867415
C	-0.1151834737	5.4954236365	-0.7325281535
C	-0.1151834626	5.4954237578	0.7325281482
C	2.2734146428	4.9980287144	0.7325662985
C	2.2734146035	4.9980286720	-0.7325663010
C	1.1077781029	5.3844558419	1.4094878551
C	1.1077780459	5.3844556343	-1.4094878099
H	1.1030905749	5.3600573588	-2.4941441879
H	1.1030906244	5.3600576081	2.4941443288
H	4.9594513203	-2.2679621074	-2.4939308036
C	4.9804653456	-2.2773217638	-1.4092469926
C	4.9804649863	-2.2773216060	1.4092469106
C	4.3491286283	-3.3307594653	-0.7323831298
C	4.3491284891	-3.3307593618	0.7323831376
H	4.9594508910	-2.2679619091	2.4939305484
C	3.5058356344	-4.2227168714	-1.4097010386

C	3.5058353720	-4.2227165560	1.4097009536
C	5.3557490328	-1.1080567214	0.7323967722
C	5.3557491827	-1.1080567420	-0.7323967682
C	5.4710085708	0.1145597648	1.4093330049
C	5.4710088666	0.1145597818	-1.4093330687
H	5.4476688133	0.1141558632	-2.4940174848
H	5.4476684553	0.1141558583	2.4940172904
C	5.3057977044	1.3314577882	0.7323980240
C	5.3057977935	1.3314578267	-0.7323980230
H	3.3098664084	4.3443336843	-2.4942418507
C	3.3256644520	4.3646776684	-1.4096446437
C	3.3256644901	4.3646776911	1.4096446519
C	4.2069420819	3.5101642501	-0.7323827951
C	4.2069420766	3.5101642210	0.7323827947
H	3.3098664361	4.3443337234	2.4942418776
C	4.8818949267	2.4839996284	-1.4092365098
C	4.8818948073	2.4839995509	1.4092364785
H	4.8607728505	2.4734492749	2.4938975669
H	4.8607730028	2.4734493505	-2.4938976602
H	-4.8607728026	-2.4734500135	2.4938977907
C	-4.8818947513	-2.4840003911	1.4092365583
C	-4.8818946318	-2.4840003136	-1.4092365272
H	-4.8607726501	-2.4734499378	-2.4938976975
C	-5.3057978568	-1.3314585549	-0.7323980894

C	-5.3057979459	-1.3314585934	0.7323980883
C	-5.4710090649	-0.1145604138	-1.4093331682
C	-5.4710093607	-0.1145604308	1.4093332319
H	-5.4476691053	-0.1141564440	2.4940179323
H	-5.4476687474	-0.1141564390	-2.4940177381
C	-5.3557496874	1.1080563344	-0.7323968978
C	-5.3557498373	1.1080563550	0.7323968937
C	-4.3491293191	3.3307597396	0.7323832904
C	-4.3491291799	3.3307596361	-0.7323832980
C	-4.9804661612	2.2773217264	1.4092472381
C	-4.9804658019	2.2773215687	-1.4092471561
H	-4.9594514139	2.2679617798	-2.4939312228
H	-4.9594518433	2.2679619779	2.4939314780
H	-3.3098658320	-4.3443340021	-2.4942416629
C	-3.3256637711	-4.3646779447	-1.4096445732
C	-3.3256637332	-4.3646779220	1.4096445649
C	-4.2069415972	-3.5101647917	-0.7323827850
C	-4.2069416025	-3.5101648208	0.7323827852
H	-3.3098658044	-4.3443339630	2.4942416359
C	-2.2734138530	-4.9980285812	0.7325662182
C	-2.2734138922	-4.9980286235	-0.7325662158
C	-1.1077773721	-5.3844551431	1.4094876227
C	-1.1077774291	-5.3844553506	-1.4094876679
H	-1.1030900559	-5.3600573302	-2.4941438158

H	-1.1030900065	-5.3600570811	2.4941436750
C	0.1151838919	-5.4954229537	0.7325280174
C	0.1151838808	-5.4954230749	-0.7325280120
C	2.4809813053	-4.8995930281	-0.7325773820
C	2.4809812372	-4.8995928800	0.7325773893
C	1.3325502721	-5.3344789430	-1.4095226649
C	1.3325501958	-5.3344786049	1.4095225927
H	1.3268773589	-5.3105139546	2.4941860331
H	1.3268774615	-5.3105143525	-2.4941862590
H	3.4898715352	-4.2037323855	2.4943334176
H	3.4898718479	-4.2037327578	-2.4943336839
